# Causal associations between the insulin-like growth factor family and sarcopenia: a bidirectional Mendelian randomization study

**DOI:** 10.3389/fendo.2024.1422472

**Published:** 2024-10-23

**Authors:** Jili Liu, Meng Chen, Xin Xia, Zhaolin Wang, Yanqin Wang, Ling Xi

**Affiliations:** ^1^ Department of Geriatrics, The First Hospital, Shanxi Medical University, Taiyuan, Shanxi, China; ^2^ Department of Geriatrics and Special Needs Medicine, General Hospital of Ningxia Medical University, Yinchuan, Ningxia, China; ^3^ The Center of Gerontology and Geriatrics and National Clinical Research Center for Geriatrics, West China Hospital, Sichuan University, Chengdu, Sichuan, China; ^4^ Department of Traditional Chinese Medicine, The Second Hospital, Shanxi Medical University, Taiyuan, Shanxi, China; ^5^ Department of Hematology, Shanxi Hospital of Traditional Chinese Medicine, Taiyuan, Shanxi, China

**Keywords:** sarcopenia, insulin-like growth factor family, insulin-like growth factor-1, Mendelian randomization, bidirectional

## Abstract

**Objective:**

Insulin-like growth factor (IGF) is closely associated with sarcopenia, yet the causal relationship of this association remains unclear. This study aims to explore the potential causal relationship between members of the IGF family and sarcopenia from a genetic perspective through bidirectional Mendelian randomization (MR) analysis using two-sample datasets.

**Methods:**

Five genetically predicted factors of the IGF family (IGF-1, IGF-1R, IGF-2R, IGFBP-3, IGFBP-7) as one sample, while four relevant features of sarcopenia (low hand grip strength, appendicular lean mass, whole body fat-free mass, and walking pace) as another sample, in conducting a two-sample MR analysis.

**Results:**

The forward MR results of the relationship between IGF and sarcopenia showed that elevated levels of IGF-1 reduced the risk of low hand grip strength (OR = 0.936, 95% CI=0.892-0.983, P = 0.008) and increased appendicular lean mass of the extremities and whole body fat-free mass (OR = 1.125, 95% CI=1.070-1.182,P = 0.000; OR =1.076, 95% CI=1.047-1.106, P=0.000), reduced the risk of sarcopenia. Elevated IGF-1R also favored an increase in whole body fat-free mass (OR=1.023, 95% CI=1.008-1.038, P =0.002), and the appendicular lean mass trait was more pronounced with elevated IGFBP-3 and IGFBP-7 (OR=1.034, 95% CI=1.024-1.044, P =0.000; OR=1.020, 95% CI=1.010-1.030, P=0.000). Inverse MR results of the effect of sarcopenia on IGF showed that decreased hand grip strength may elevate IGF-1 levels (OR=1.243, 95% CI=1.026-1.505,P =0.027), whereas improvements in appendicular lean mass, whole body fat-free mass traits, and increased walking pace decreased IGF-1 levels (OR=0.902, 95% CI: 0.877-0.927, P = 0.000; OR=0.903, 95% CI=0.859-0.949,P = 0.000; OR=0.209, 95% CI=0.051-0.862,P = 0.045). Also decreased hand grip strength may elevate IGF-1R levels (OR=1.454, 95% CI=1.108-1.909, P =0.007), and appendicular lean mass stimulated high expression of IGFBP-1 (OR=1.314, 95% CI=1.003-1.722, P =0.047). Heterogeneity and pleiotropy were not detected in all results, and the results were stable and reliable.

**Conclusion:**

There is a bi-directional causal association between IGF family members and the risk of sarcopenia, which provides a more adequate basis for early biological monitoring of sarcopenia and may provide new targets for early intervention and treatment of sarcopenia.

## 1 Introduction

Sarcopenia is a degenerative condition of skeletal muscle that is intricately associated to the aging process, characterized by a decline in muscle mass, diminished muscle strength, and compromised somatic function. The definition of sarcopenia has been revised by 2018 European Working Group on Sarcopenia in Older People 2 (EWGSOP2) to underscore the significance of reduced muscle strength as the key criterion for diagnosing sarcopenia, with reductions in muscle quantity and mass serving as foundational indicators, and impaired physical function serving as a hallmark of advanced sarcopenia (1).Sarcopenia, a common geriatric syndrome characterized by a high prevalence, insidious onset, and difficulty in early recognition, is more prevalent in the elderly population due to its close association with age. A comprehensive assessment of sarcopenia’s global prevalence among individuals aged 60 years and older yielded estimates ranging from 10% to 27% (2). Additionally, sarcopenia is associated with cardiovascular and metabolic diseases (3), respiratory diseases (4), and cognitive dysfunction (5). Moreover, it has been shown to elevate the likelihood of falls, fractures, and disability (6, 7), restrict mobility and impair the ability to engage in daily activities (8), and potentially necessitate long-term nursing care or a loss of independence in older individuals (9–11). These outcomes have a profound effect on quality of life (12), increase the risk of mortality (13), and place a significant strain on individuals, society, and the healthcare system (14). The etiology and pathogenesis of sarcopenia remain poorly understood, with a dearth of efficacious pharmacological interventions (15). Therefore, it is imperative to investigate the precise pathogenesis of sarcopenia, identify individuals with sarcopenia in its early stages, and explore viable therapeutic strategies to mitigate the potential for adverse outcomes.

The insulin-like growth factor (IGF) is a multifaceted system comprising two ligands (IGF-1 and IGF-2), two receptors (IGF-1R and IGF-2R), six high-affinity binding proteins (IGFBP1-6), and a cohort of low-affinity IGFBP-associated proteins (IGFBP-7, etc.), along with IGFBP proteases (16). This intricate system engages in a complex web of interactions both internally and with other growth factor families and their associated signaling pathways, ultimately regulating crucial cellular processes such as proliferation, differentiation, glucose and lipid metabolism, and cell survival (17). IGF-1 stands out as a pivotal growth factor that modulates both anabolic and catabolic processes in skeletal muscle, potentially serving as an essential factor in sarcopenia by promoting skeletal muscle protein synthesis, impeding muscle atrophy, and bolstering muscle regeneration via multiple pathways (18, 19). Despite numerous foundational researches corroborating a relationship between IGF and sarcopenia, the causality of this association remains ambiguous. Mendelian randomization (MR) study using instrumental variables (IV) from the Genome-Wide Association Study (GWAS) dataset, employing a genetic variation approach that mitigate potential confounding factors and reverse causation. Mendel’s law of independent distribution states that the intermediate genes are randomly assigned to the progametes during gametic formation. And outcome measures are not affected by confounding variables, therefore, this study utilized two-sample bidirectional MR to explore the potential causal relationship between IGF family members and sarcopenia.

## 2 Methods

### 2.1 Study design

**Figure 1:**
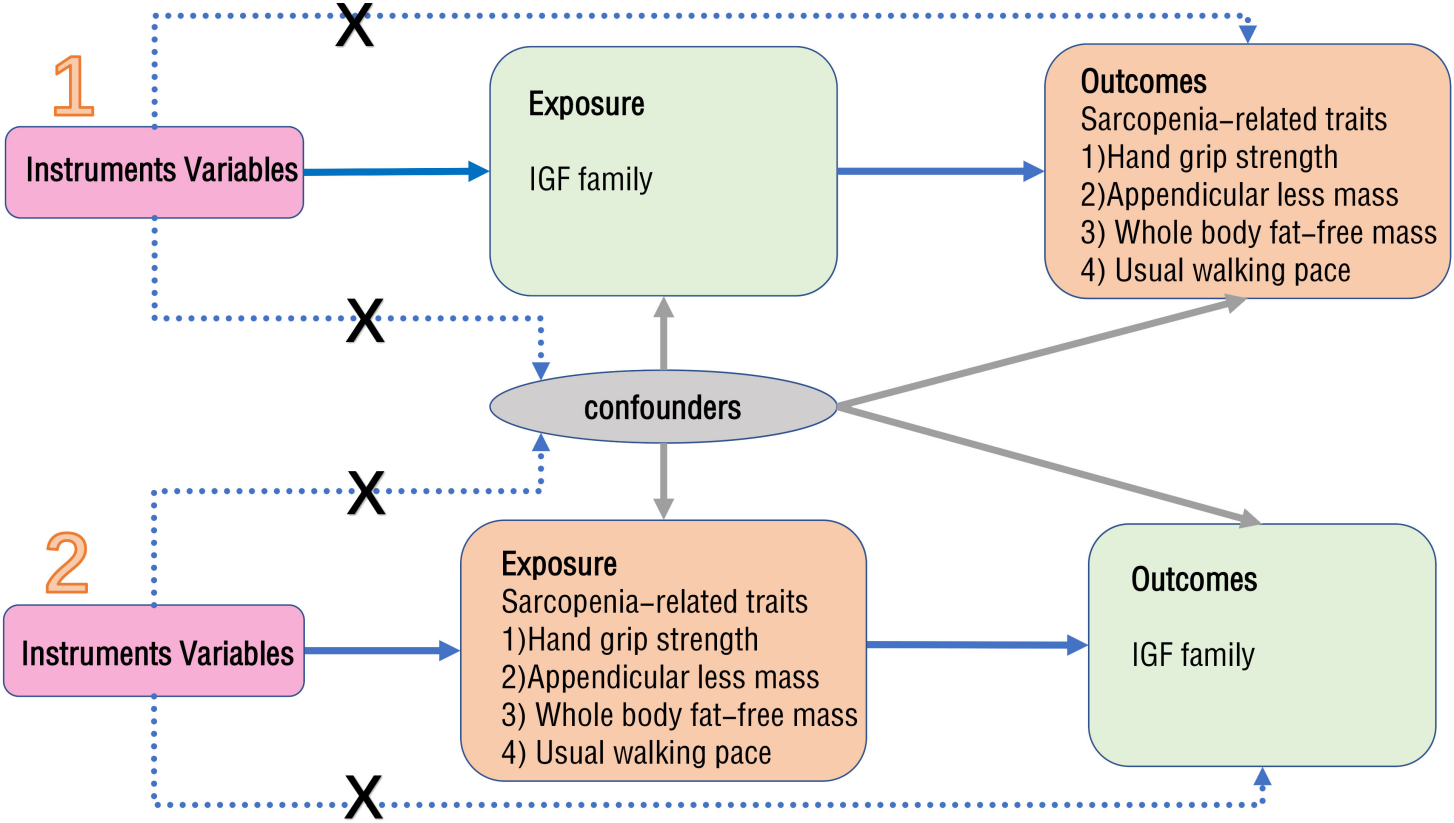
The flowchart of the bidirectional MR study.

MR is a method that utilizes exposure-related genetic variants as instrumental variables (IVs) to evaluate the causal effect of exposure on clinical outcomes of interest (20). The design flowchart is presented in **Figure 1**. When conducting MR analysis, it is essential that the single nucleotide polymorphisms (SNPs) chosen as IVs meet three assumptions: they must exhibit a strong association with the exposure factor, not be linked to any other confounding factors, and solely influence the outcome through the exposure factor without affecting it through alternative pathways. The validity of the experimental data hinges on the simultaneous satisfaction of three assumptions.

A two-sample MR approach was employed to identify robust factors from the 10 principal components of the IGF family, consisting of IGF-1, IGF-2, IGF-1R, IGF-2R, IGFBP-1, IGFBP-2, IGFBP-3, IGFBP-4, IGFBP-5, IGFBP-6, and IGFBP-7, for incorporation in this investigation. In order to ensure the reliability of the results, SNPs strongly associated with exposure were selected based on genome-wide significance threshold criteria (p<5×10^-8^) to serve as IVs. Ultimately, five major members (IGF-1, IGF-1R, IGF-2R, IGFBP-3, and IGFBP-7) were screened as potential SNPs as a sample for analysis. Additionally, potential SNPs were identified as another sample based on characteristics of sarcopenia, including low hand grip strength, appendicular lean mass, whole body fat-free mass, and usual walking pace. In the framework of forward MR analysis, the members of IGF family were utilized as exposures to investigate their impact on sarcopenia. Conversely, in the reverse MR study, sarcopenia-related traits were employed as exposures to explore their potential causal association with IGF family members.

### 2.2 Data sources

#### 2.2.1 IGF family data sources

SNPs for IGF family members were extracted from publicly available genome-wide association studies (GWAS) conducted by from UK Biobank. The analysis encompassed three distinct cohort studies: the UK Biobank cohort (21) involving 49,960 cases and 385,556 controls, the KORA cohort study (22) with 1,000 subjects, and the INTERVAL study (23) with 3,301 healthy participants. In total, the study enrolled 439,817 participants of European ancestry, encompassing 15,267,522 SNPs.

#### 2.2.2 Data sources for sarcopenia-related traits

Sarcopenia-associated traits include low hand grip strength, appendicular lean mass, whole body fat-free mass, and usual walking pace. Pooled data on low hand grip strength were obtained from a meta-analysis of comprehensive GWAS involving 256,523 individuals, including 48,596 cases of low hand grip strength and 207,927 controls of European descent aged 60 years or older from the CHARGE consortium. The analysis incorporated data from 22 different cohorts, such as the American Health and Retirement Study, the UK Biobank, and the Framingham Heart Study (FHS). The study recorded the maximum hand grip strength, establishing a low-strength threshold of less than 20 kg for women and 30 kg for men (24). Summary statistics for appendicular lean mass (ALM) were derived from a GWAS involving 450,243 UK Biobank participants, comprising 244,730 women and 205,513 men (25). Additionally, summary statistics for usual walking pace and whole body fat-free mass were extracted from GWAS datasets of 335,349 and 454,850 individuals of European ancestry in the UK Biobank, respectively, which were accessible at https://gwas.mrcieu.ac.uk/datasets/ukb-a-513/, and https://gwas.mrcieu.ac.uk/datasets/ukb-b-13354/. Information on the data sources for IGF identified members and the characteristic traits of sarcopenia is shown in **Table 1**.

**Table 1:** Details of the exposure and outcome data.

Traid	GWAS id	Age Median(25^th^-75th percentile) or Mean (± SD)	Sex Women	Sample size	Number of SNPs
IGF					
IGF-1	ebi-a-GCST90025989	58 (45–71)	54.5%	435,516	4,231,359
IGF-1 R	prot-a-1444	43.7(± 14.3)	48.9%	3,301	10,534,735
IGF-2 R	prot-c-3676_15_3	59 (53-67)	52.9%	1,000	501,428
IGFBP-1	prot-c-2771_35_2	59 (53-67)	52.9%	1,000	501,428
IGFBP-2	prot-c-2570_72_5	59 (53-67)	52.9%	1,000	501,428
IGFBP-3	prot-c-2571_12_3	59 (53-67)	52.9%	1,000	501,428
IGFBP-4	prot-c-2950_57_2	59 (53-67)	52.9%	1,000	501,428
IGFBP-5	prot-c-2685_21_2	59 (53-67)	52.9%	1,000	501,428
IGFBP-6	prot-c-2686_67_2	59 (53-67)	52.9%	1,000	501,428
IGFBP-7	prot-c-3320_49_2	59 (53-67)	52.9%	1,000	501,428
Sarcopenia					
Low hand grip strength (60 years and older)	ebi-a-GCST90007526	Age>=60	52.8%	256,523	9,336,415
Appendicular lean mass	ebi-a-GCST90000025	56.7(± 8.0)	54.4%	450,243	18,071,518
Whole body fat-free mass	ukb-b-13354	not described	not described	454,850	9,851,867
Usual walking pace	ukb-a-513	not described	not described	335,349	10,894,596

The data were all collected from a European population.

### 2.3 Instrumental variables

The IV selection process adhered to the three assumptions of MR. SNPs that exhibited a strong association with the exposure variable at the genome-wide significance threshold (p < 5×10^-8^) were extracted as IVs. Additionally, all SNPs were required to satisfy linkage disequilibrium at a distance of 10,000 kb (r^2^ < 0.001) in order to ignore the superimposition effect of the associated SNPs to determine their independence. In order to mitigate the impact of confounding variables, SNPs linked to the outcome were omitted through a search conducted in PhenoScanner V2 (26), subsequently employing Mendelian Randomization Pleiotropy RESidual Sum and Outlier (MR-PRESSO) to eliminate any potentially outlying SNPs. The R^2^ and F statistic were calculated to evaluate the robustness of IVs, with IVs exhibiting an F statistic below 10 being excluded to mitigate the bias introduced by weak IVs (27).

### 2.4 MR analysis

Two-sample MR analysis was performed to assess the relationship between exposure and outcome, utilizing the TwoSampleMR package (version 0.5.6) in R software (version 4.3.2). Following the selection of valid SNPs, MR analysis was primarily carried out using inverse variance weighting (IVW) (28, 29). Meanwhile, weighted median estimator (WME) and MR-Egger regression were employed as supplementary analysis methods to IVW (30). When utilizing a single SNP as IVs for an exposure factor, the Walt ratio (WR) was employed to evaluate the causal impact of the exposure variable on the outcome variable. Estimates from MR analysis were presented as beta (β) coefficients, odds ratios (ORs), and their associated 95% confidence intervals (CI), with statistical significance determined at a threshold of P<0.05. A Benjamini & Hochberg (BH) correction was considered to adjust the P value.

### 2.5 Reliability assessment

In order to ensure the reliability and robustness of the results, a series of sensitivity analyses were performed, encompassing Cochran’s Q test, MR-Egger intercept test, funnel plot, and leave-one-out analysis. The P value of Cochran’s Q test ≥ 0.05 indicated the absence of heterogeneity (31). Horizontal pleiotropy was assessed using MR-Egger’s intercept analysis, with P ≥ 0.05 suggesting the absence of horizontal pleiotropy in SNPs (30). Funnel plots were employed for visual inspection of effects and estimation of distribution symmetry. Any observed asymmetry in the funnel plot may suggest the presence of heterogeneity. Sensitivity analyses were performed utilizing the leave-one-out method to determine the reliability and robustness of the results by assessing whether each overall estimate was driven by a single SNP.

## 3 Results

### 3.1 Forward MR

**Figure 2:**
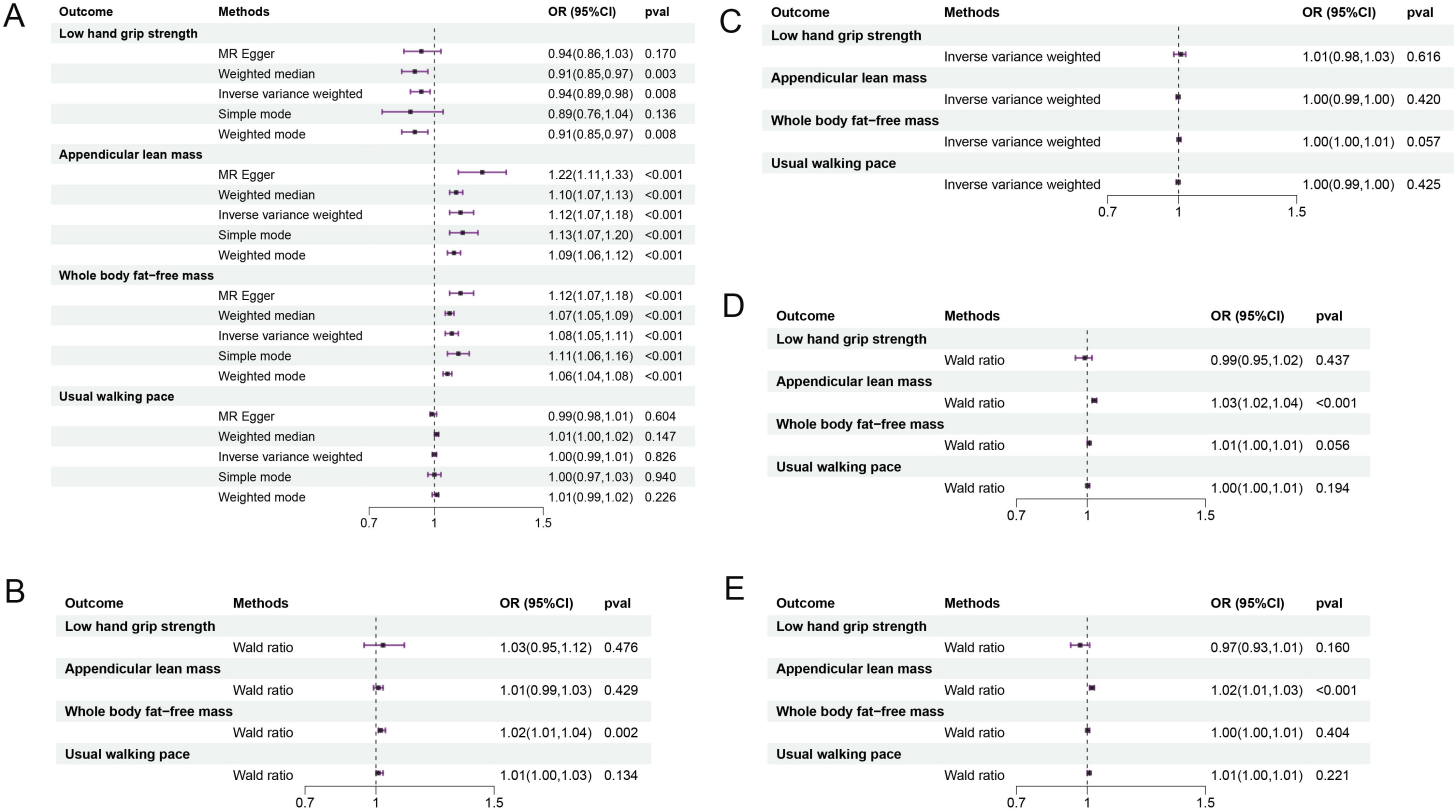
The results of forward MR analysis. The IGF family members were used as exposure factors. **(A)** IGF-1 and sarcopenia; **(B)** IGF-1R and sarcopenia; **(C)** IGF-2R and sarcopenia; **(D)** IGFBP-3 and sarcopenia; **(E)** IGFBP-7 and sarcopenia. IGFBP, IGF-binding protein; OR, odds ratio; CI, confidence interval.

Five components of the IGF family, namely IGF-1, IGF-1R, IGF-2R, IGFBP-3, and IGFBP-7, were incorporated for the identification of potential SNPs. The pertinent basic information is shown in **Supplementary Table S1**. As shown in **Figure 2**, the results of IVW analysis revealed that elevated levels of IGF-1 reduced the risk of hand grip strength loss (OR = 0.936, 95% CI: 0.892- 0.983, P = 0.008), and improve the traits of Appendicular lean mass in the extremities and whole body fat-free mass (OR = 1.125, 95% CI: 1.070-1.182,P = 0.000; OR = 1.076, 95% CI=1.047-1.106, P = 0.000), but the association with usual walking pace was not significant (OR=1.001, 95% CI=0.991-1.012, P=0.826). The Wald ratio showed that elevated IGF-1R levels significantly improved whole body fat-free mass traits (OR=1.023, 95% CI=1.008-1.038, P =0.002), and IGFBP-3, IGFBP-7 were positively correlated with appendicular lean mass of the extremities (OR=1.034, 95% CI=1.024-1.044, P = 0.000; OR = 1.020, 95% CI=1.010-1.030, P = 0.000), improving muscle mass and reducing the risk of sarcopenia. No correlation was found between IGF-2R and the various sarcopenia-related traits. Detailed results are shown in **Supplementary Table S2**. In subsequent sensitivity analyses, there was no heterogeneity among IGF-1, IGF-1R, IGFBP3, and IGFBP7, nor was potential horizontal pleiotropy found between IGF-1 and hand grip strength, appendicular lean mass of the extremities, and whole body fat-free mass (intercept = -0.000, P = 0.734; intercept = -0.002, P = 0.058; intercept = -0.001, and P = 0.055). The pvalue of Cochran’s Q test were greater than 0.1, and leave-one-out analyses showed that the causal effects were not influenced by a particular instrumental variable, showing good stability of the results (**Supplementary Figures S1**, **S2**).

### 3.2 Reverse MR

**Figure 3:**
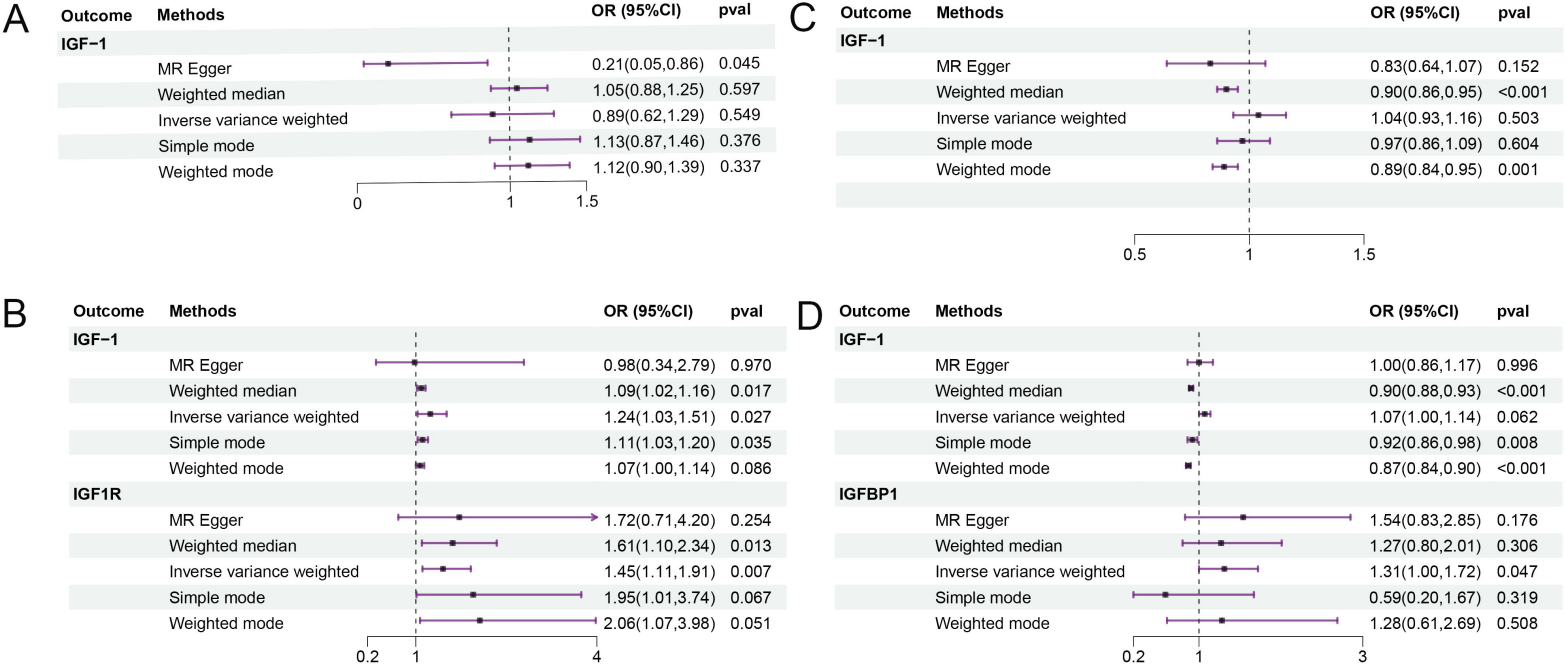
The results of reverse MR analysis. Sarcopenia-related traits were used as exposure factors. **(A)** usual walking pace and IGF; **(B)** low hand grip strength and IGF; **(C)** whole body fat-free mass and IGF; **(D)** appendicular lean mass and IGF. OR, odds ratio; CI, confidence interval.

Sarcopenia-related traits were used as exposure to identify potential SNPs, and the relevant basic information is shown in **Supplementary Table S3**. As shown in **Figure 3**, the results of IVW analysis showed that decreased hand grip strength significantly elevated IGF-1 levels (OR=1.243, 95% CI=1.026-1.505, P =0.027) and IGF-1R levels (OR=1.454, 95% CI=1.108- 1.909, P =0.007), and appendicular lean mass in the extremities may also elevate IGFBP-1 levels (OR=1.314, 95% CI=1.003-1.722, P =0.047). The weighted median method showed that lean limb weight and whole body fat-free mass were negatively associated with IGF-1 (OR=0.902, 95% CI=0.877-0.927, P =0.000; OR=0.903, 95% CI=0.859-0.949, P =0.000), which significantly lowered IGF-1 levels.MR Egger’s analysis of usual walking pace lowered IGF-1 levels (OR=0.209, 95% CI=0.051-0.862, P =0.045). Detailed results are shown in **Supplementary Table S4**. In subsequent sensitivity analyses, no heterogeneity was found for low hand grip strength, appendicular lean mass in extremities, whole body fat-free mass, and walking pace, while there was no pleiotropy between low hand grip strength, appendicular lean mass in extremities, whole body fat-free mass, and walking pace and IGF-1 (intercept = 0.006, P = 0.801; intercept = 0.002, P = 0.319; intercept = 0.004, P = 0.057; intercept = 0.015, P = 0.054), and no horizontal pleiotropy was seen for low hand grip strength with IGF-1R (intercept = -0.017, P = 0.493), or for appendicular lean mass in extremities with IGFBP-1 (intercept = -0.005, P = 0.479). Leave-one-out analyses showed that the results have good stability (**Supplementary Figures S3**-**S6**).

## 4 Discussion

The present two-sample MR study was the comprehensive assessment of the causal relationship between IGF family members and sarcopenia from a genetic perspective using extensive genome-wide association studies (GWAS) summary statistics. As a result, we successfully identified a causal effect between IGF family members and sarcopenia, with higher levels of IGF-1, IGF-1R, IGFBP-3, and IGFBP-7 being associated with a reduced risk of sarcopenia. Specifically, IGF-1 was found to significantly decrease the likelihood of low hand grip strength and increase lean mass of and whole body fat-free mass, with no significant correlation observed with walking pace. Additionally, no significant association was found between IGF-2R and the risk of sarcopenia. Furthermore, the controversial results were yielded in the analysis of the relationship between sarcopenia and IGF family, demonstrating that despite decreases in hand grip strength, limbs lean mass, and whole body fat-free mass, as well as walking pace, the levels of IGF-1 were elevated, indicating a potential causal relationship between sarcopenia and IGF-1.

Previous studies on the mechanisms of sarcopenia have already revealed that changes in hormone levels play an essential role and that components of the endocrine system, such as IGF-1, growth hormone (GH), as well as androgens, are the most important regulators of muscle metabolism and muscle mass (32–34). With increasing age, the levels of these hormones may decline, leading to decreased muscle synthesis and increased catabolism, thereby promoting the development of sarcopenia. The IGF family consists of polypeptide growth factors that share structural similarities with insulin. Their biological functions are predominantly mediated by the formation of complexes with IGF-binding proteins (IGFBPs), which protects them from premature degradation, thereby maintaining their serum levels. Simultaneously, IGF ligands are released and bind to the corresponding IGF receptors at the appropriate time and location, initiating signaling cascades that facilitate cellular growth and differentiation (35).

Several observational studies conducted in diverse populations have contributed valuable insights to our research. For example, a survey of middle-aged and elderly Japanese adults demonstrated a positive association between IGF-1 levels and skeletal muscle mass index, hand grip strength, and walking pace (36). Similarly, a European study found that lower levels of IGF-1 were correlated with slower walking pace in men aged 70 and above (37). Additionally, a study from Taiwan, China indicated that elevated serum IGF-1 levels were independently correlated with increased muscle mass, higher bone mineral density, and stronger hand grip strength in men as opposed to women (38). Furthermore, a study in the Netherlands demonstrated that decreased serum levels of IGF-1 are associated with diminished physical performance and lower hand grip strength in elderly adults (39). In the present study, we mitigated the potential confounding factors inherent in epidemiological research and derived causal inferences from a genetic standpoint. Elevated levels of IGF-1 were found to significantly reduced the risk of low hand grip strength and promote enhancements in muscle strength (40). Additionally, heightened IGF-1 levels were correlated with a substantial rise in appendicular lean mass and whole body fat-free mass, ultimately enhancing muscle quality and acting as a robust protective factor against sarcopenia. Recent mechanistic investigations have demonstrated the significance of IGF-1 in promoting skeletal muscle mass through the stimulation of satellite cell proliferation and fusion, facilitating muscle tissue regeneration, myoblast proliferation, and differentiation in the context of normal growth or skeletal muscle regeneration after injury (41). Furthermore, IGF-1 has been shown to enhance protein synthesis and reduce protein hydrolysis (18). Local IGF-1, acting as a paracrine/autocrine growth factor, also contributes significantly to the maintenance of muscle mass (42). However, our study could not establish a causal relationship between IGF-1 levels and walking pace. Walking pace, serving as an indicator of diminished physical function, is a marker of the severity of sarcopenia. The initial phases of the disease are characterized by a decline in muscle strength and muscle mass, with hand grip strength being a trait manifestations of muscle strength, and appendicular lean mass and whole body fat-free mass being a trait manifestations of muscle number and muscle mass (43). The observed positive correlation between IGF-1 levels and reductions in hand grip strength, appendicular lean mass, as well as whole body fat-free mass suggests that IGF-1 may serve as a potential biomarker for early detection of sarcopenia and monitoring disease progression.

In addition to IGF-1, within the IGF family, we found that IGF-1R increased whole body fat-free mass, and IGFBP-3 and IGFBP-7 increased appendicular lean mass, and improved muscle mass and muscle bulk, demonstrating that high IGF-1R, IGFBP-3, and IGFBP-7 levels may reduce the risk of sarcopenia. Furthermore, IGF-1 primarily exerts its effects through IGF-1R, which is a transmembrane receptor with tyrosinase activity. Upon binding, it stimulates protein synthesis in skeletal muscle through the PI3K/Akt/mTOR and PI3K/Akt/GSK3β pathways, ultimately inducing skeletal muscle hypertrophy (44–46). Consistent with the direction of action of IGF-1, it increases muscle mass and reduces the risk of sarcopenia, as verified by our findings. The activity of IGF-1 is intricately controlled by the family of plasma transport proteins of IGFBP (47, 48). IGFBP-3, primarily synthesized in the liver, is the principal transporter of IGF-1 in plasma. Studies have indicated that IGFBP-3 can inhibit cell growth and promote apoptosis through a non-IGF-dependent mechanism (48). Furthermore, a cross-sectional analysis involving 131 elderly individuals revealed a noteworthy decrease in IGFBP-3 levels among patients with sarcopenia (49), implying a contrasting function of IGFBP-3 compared to IGF-1 in skeletal muscle. However, conflicting findings exist regarding the clinical significance of IGFBP-3 in sarcopenia, as one study suggests that approximately 95% of circulating IGF-1 binds to IGFBP-3 and acid labile subunit (ALS) to form a ternary complex, hindering the translocation of IGF-1 into the intracellular compartment and consequently extending its serum half-life and regulating its availability (50).The data regarding the clinical role of IGFBP-3 in sarcopenia are characterized by inconsistency and contradiction. Our findings demonstrate a positive correlation between IGFBP-3 and appendicular lean mass, suggesting a protective role of IGFBP-3 in sarcopenia by promoting muscle mass and reducing the likelihood of sarcopenia development. Additionally, IGFBP-7 is expressed in multiple tissues, such as the pancreas, brain, skeletal muscle and liver. As research advances, IGFBP-7 is emerging as a potential biomarker for various diseases such as acute kidney injury, heart failure, and cancer (51–53). Despite this, its association with sarcopenia remains understudied. This study reveals that IGFBP-7 may have a positive impact on appendicular lean mass in limbs and could potentially reduce the risk of sarcopenia. Further investigation is necessary to determine whether IGFBP-7 can serve as a biomarker for sarcopenia in the future. These findings also provide novel insights and potential therapeutic targets for the development of specific treatments for sarcopenia.

In the reverse Mendelian randomization study, the findings indicate that the decreased hand grip strength led to increased IGF-1 and IGF-1R levels, whereas appendicular lean mass and whole body fat-free mass decreased IGF-1 levels, suggesting a potential causal association between sarcopenia and elevated IGF-1 levels. However, a comprehensive review of the literature reveals notable discrepancies in the impact of muscle strength and muscle mass on IGF-1 levels across various studies. For instance, a cross-sectional analysis involving 3,276 elderly participants demonstrated that serum IGF-1 levels were lower in individuals with sarcopenia compared to those without this condition (54).Another study involving 27 healthy students demonstrated that after 12 weeks of high-intensity resistance training and moderate-intensity endurance training, there was a notable increase in muscle strength and endurance, while IGF-1 levels decreased significantly in all participant groups post-intervention (55). Furthermore, an exercise intervention trial involving elderly obese women with sarcopenia revealed a significant elevation in IGF-1 levels following a 12-week circuit training program (56). In a recent study involving 36 sedentary middle-aged female workers who underwent 8 weeks of exercise training, it was found that muscle mass and muscle strength were effectively improved, but there was no significant change in IGF-1 levels (57). However, these studies presented us with discrepant results, and lacked deep mechanistic analyses and explanations. We predicted for the increase in IGF-1 levels due to sarcopenia by genetic variation, and analyzed the possible reasons. The production of IGF-1 is influenced by various factors, including changes in circulating hormone levels and metabolic demands of local muscle tissue. In the early stages of sarcopenia, there may exist some mechanism that stimulates the production of IGF-1, contributing to a temporary increase in IGF levels in an effort to maintain normal muscle mass and strength. However, this stimulation may gradually diminish as the disease progresses, eventually resulting in a decrease in IGF-1 levels. However, it is important to note that the aforementioned statement is merely a hypothesis, and further rigorous research is required to explore the impact of varying stages of sarcopenia, diverse muscle strengths, differing muscle masses, and physical function statuses on IGF levels. Furthermore, our investigation revealed a positive correlation between appendicular lean mass in the extremities and IGFBP-1, indicating a potential association between sarcopenia and IGFBP-1 levels. A cross-sectional analysis involving 4,908 women aged between 55 and 85 years demonstrated that individuals with elevated IGFBP-1 levels were more likely to exhibit reduced muscle mass, suggesting that high IGFBP-1 levels may be a marker of catabolic metabolism (58). These findings appear to be incongruent with our research, prompting the need for additional investigation to reconcile these seemingly contradictory results.

The innovation of this study lies in the MR-derived design, which systematically evaluates the causal association between IGF family members and sarcopenia from a genetic perspective. This design effectively minimizes the biases caused by confounding factors, and provides a valuable framework for foundational investigations. The dataset of this study exhibits no pleiotropy or heterogeneity, ensuring the reliability and stability of the results. It may contribute to providing novel insights and targets for the biological monitoring and effective treatment of sarcopenia in the future. We acknowledge several limitations. First, the sample was drawn from a European population and may not be generalizable to other populations. Therefore, further research involving diverse racial populations is necessary to confirm the validity of our results. Second, MR studies are constrained by limitations pertaining to variable selection and data source quality, sarcopenia may also be associated with habitual physical activity, health status and sex of the participants (59, 60). These data were not available due to a lack of detailed records. Furthermore, the discrepancy between the association between sarcopenia and IGF in reverse MR and the results of several previous studies raises novel ideas that need to be justified and explained by more relevant studies.

## 5 Conclusion

Genetic evidence obtained through Mendelian randomization analyses supports a causal relationship between IGF family members and sarcopenia. Elevated levels of IGF-1, IGF-1R, IGFBP-3, and IGFBP-7 within the IGF family are associated with a decreased risk of sarcopenia, indicating a causal associated with sarcopenia. These factors may be involved in the future as potential predictive biomarkers for screening and monitoring sarcopenia progression in its early stages. Furthermore, they may offer novel targets for the development of targeted treatments for sarcopenia. The reverse study demonstrates a positive correlation between sarcopenia and IGF-1, as well as IGFBP-1. Further research is needed to investigate the dynamic changes in IGF levels across various stages of sarcopenia.

## Data Availability

No original, unprocessed data was used in present study. The summary datasets used in our study were derived from the following resources available in the public domain, which can be accessed at: https://gwas.mrcieu.ac.uk/. Further inquiries can be directed to the corresponding author.

## Supplementary material

Supplementary Table 1 List of SNPS in the IGF family.

Supplementary Table 2 The results of forward MR analysis.

Supplementary Table 3 List of SNPS associated with sarcopenia.

Supplementary Table 4 The results of reverse MR analysis.

Supplementary Figure 1 The SNPs effect estimate based on the forward MR analysis. **(A)** IGF-1 on appendicular lean mass; **(B)** IGF-1 on low hand grip strength; **(C)** IGF-1 on usual walking pace; **(D)** IGF-1 on whole body fat-free mass. SNPs, single nucleotide polymorphisms.

Supplementary Figure 2 The funnel plot performed by the forward MR analysis. **(A)** IGF-1 on appendicular lean mass; **(B)** IGF-1 on low hand grip strength; **(C)** IGF-1 on usual walking pace; **(D)** IGF-1 on whole body fat-free mass.

Supplementary Figure 3 The SNPs effect estimate based on the reverse MR analysis. **(A)** usual walking pace on IGF-1; **(B)** low hand grip strength on IGF-1; **(C)** low hand grip strength on IGF-1R; **(D)** whole body fat-free mass on IGF-1; **(E)** appendicular lean mass on IGF-1; **(F)** appendicular lean mass on IGFBP-1. SNPs, single nucleotide polymorphisms.

Supplementary Figure 4 The forest plot performed by the reverse MR analysis. **(A)** usual walking pace on IGF-1; **(B)** low hand grip strength on IGF-1; **(C)** low hand grip strength on IGF-1R; **(D)** appendicular lean mass on IGF-1.

Supplementary Figure 5 The sensitivity analyses performed by the reverse MR analysis. **(A)** usual walking pace on IGF-1; **(B)** low hand grip strength on IGF-1; **(C)** low hand grip strength on IGF-1R.

Supplementary Figure 6 The funnel plot performed by the reverse MR analysis. **(A)** usual walking pace on IGF-1; **(B)** low hand grip strength on IGF-1; **(C)** low hand grip strength on IGF-1R; **(D)** whole body fat-free mass on IGF-1; **(E)** appendicular lean mass on IGF-1; **(F)** appendicular lean mass on IGFBP-1.
