# Supplementary material for: Causal associations between the insulin-like growth factor family and sarcopenia: a bidirectional Mendelian randomization study
Source: Front Endocrinol (Lausanne). 2024 Oct 23;15:1422472. doi: 10.3389/fendo.2024.1422472 (PMC11537870; doi:10.3389/fendo.2024.1422472)
Supplement: Supplementary Table 1 — List of SNPS in the IGF family. [file Table1.docx]

|  | SNP | Beta | Se | R | Fscore | Pvalue |
| --- | --- | --- | --- | --- | --- | --- |
| IGF-1 |  |  |  |  |  |  |
|  | **rs1497406** | **-0.018** | **0.002** | **-0.014** | **84.005** | **9.3004E-21** |
|  | **rs1978903** | **-0.014** | **0.002** | **-0.010** | **39.998** | **3.8001E-11** |
|  | **rs75524443** | **0.026** | **0.004** | **0.009** | **34.217** | **1.4E-09** |
|  | **rs10913189** | **-0.027** | **0.004** | **-0.010** | **46.463** | **9.4995E-13** |
|  | **rs12118034** | **0.066** | **0.003** | **0.040** | **686.765** | **2.897E-154** |
|  | **rs17012555** | **0.032** | **0.005** | **0.009** | **35.492** | **4.7E-11** |
|  | **rs12141189** | **-0.044** | **0.002** | **-0.029** | **369.334** | **1.9002E-87** |
|  | **rs16824937** | **0.030** | **0.004** | **0.012** | **63.370** | **4.4999E-16** |
|  | **rs10493008** | **-0.014** | **0.002** | **-0.010** | **47.262** | **9.3004E-12** |
|  | **rs1171265** | **-0.018** | **0.002** | **-0.014** | **81.400** | **8.6E-19** |
|  | **rs9427104** | **-0.022** | **0.002** | **-0.017** | **123.425** | **1.9002E-29** |
|  | **rs2298083** | **-0.027** | **0.003** | **-0.014** | **83.279** | **1.5999E-20** |
|  | **rs823094** | **-0.015** | **0.002** | **-0.011** | **55.660** | **3.2999E-14** |
|  | **rs116509476** | **0.048** | **0.006** | **0.012** | **61.313** | **2.0999E-15** |
|  | **rs2168812** | **-0.033** | **0.003** | **-0.020** | **165.826** | **5.1004E-40** |
|  | **rs12042441** | **0.015** | **0.003** | **0.008** | **29.385** | **3.4E-08** |
|  | **rs2311528** | **0.014** | **0.002** | **0.010** | **40.362** | **1.5E-10** |
|  | **rs165316** | **0.073** | **0.002** | **0.045** | **890.218** | **9.594E-198** |
|  | **rs55843942** | **-0.022** | **0.003** | **-0.011** | **55.847** | **2.1999E-15** |
|  | **rs655555** | **0.012** | **0.002** | **0.009** | **33.812** | **1.5E-09** |
|  | **rs12375** | **0.017** | **0.002** | **0.013** | **70.124** | **1.1E-15** |
|  | **rs11809207** | **0.024** | **0.002** | **0.015** | **91.737** | **8.9991E-24** |
|  | **rs66699565** | **-0.014** | **0.002** | **-0.010** | **40.362** | **1.8001E-11** |
|  | **rs943513** | **0.025** | **0.002** | **0.019** | **158.490** | **9.3994E-39** |
|  | **rs6659633** | **0.015** | **0.003** | **0.009** | **32.009** | **3.1E-08** |
|  | **rs56145419** | **0.018** | **0.003** | **0.008** | **30.935** | **5.3001E-09** |
|  | **rs2488249** | **-0.018** | **0.002** | **-0.014** | **79.688** | **5.3003E-19** |
|  | **rs114165349** | **-0.111** | **0.006** | **-0.026** | **293.337** | **2.9999E-67** |
|  | **rs1740610** | **0.023** | **0.002** | **0.015** | **92.031** | **1E-21** |
|  | **rs2230587** | **0.027** | **0.003** | **0.013** | **76.666** | **4.0004E-18** |
|  | **rs10874746** | **-0.020** | **0.002** | **-0.014** | **90.630** | **2.9E-22** |
|  | **rs910633** | **-0.012** | **0.002** | **-0.010** | **40.406** | **1.7E-10** |
|  | **rs2232016** | **0.014** | **0.002** | **0.009** | **36.286** | **3.5E-10** |
|  | **rs599839** | **-0.031** | **0.002** | **-0.020** | **180.630** | **3.2999E-40** |
|  | **rs6431786** | **-0.020** | **0.002** | **-0.013** | **71.113** | **2.7002E-18** |
|  | **rs1260326** | **0.067** | **0.002** | **0.050** | **1081.917** | **1E-200** |
|  | **rs113017476** | **-0.031** | **0.005** | **-0.009** | **38.430** | **2.1E-10** |
|  | **rs11691852** | **-0.014** | **0.002** | **-0.010** | **44.520** | **3.4002E-11** |
|  | **rs3755304** | **-0.024** | **0.003** | **-0.012** | **64.919** | **8.1003E-18** |
|  | **rs343964** | **0.014** | **0.003** | **0.008** | **29.451** | **3.3E-09** |
|  | **rs3791679** | **0.018** | **0.002** | **0.012** | **60.521** | **1.6998E-15** |
|  | **rs7607369** | **0.017** | **0.002** | **0.013** | **70.959** | **8.1003E-18** |
|  | **rs77666550** | **0.067** | **0.008** | **0.012** | **65.858** | **2.9999E-16** |
|  | **rs62127750** | **0.030** | **0.005** | **0.010** | **42.852** | **6.7999E-12** |
|  | **rs11545482** | **-0.089** | **0.007** | **-0.019** | **158.146** | **1.1E-37** |
|  | **rs72885919** | **0.012** | **0.002** | **0.009** | **35.957** | **2.2E-09** |
|  | **rs7572505** | **-0.019** | **0.002** | **-0.013** | **71.782** | **5.9007E-18** |
|  | **rs34967399** | **0.041** | **0.007** | **0.009** | **34.565** | **5.3001E-09** |
|  | **rs72799666** | **0.056** | **0.005** | **0.016** | **112.998** | **4.7E-27** |
|  | **rs10192766** | **0.014** | **0.002** | **0.010** | **42.803** | **1.5E-10** |
|  | **rs13411546** | **-0.012** | **0.002** | **-0.009** | **36.007** | **2.6E-09** |
|  | **rs10497015** | **-0.011** | **0.002** | **-0.009** | **31.550** | **4.8E-08** |
|  | **rs17400325** | **0.061** | **0.005** | **0.018** | **147.110** | **1.5999E-35** |
|  | **rs4675814** | **0.015** | **0.002** | **0.009** | **38.460** | **4.2E-10** |
|  | **rs1861402** | **0.020** | **0.003** | **0.009** | **33.464** | **1.4E-09** |
|  | **rs72841131** | **0.032** | **0.004** | **0.012** | **65.931** | **5.6002E-17** |
|  | **rs3761706** | **0.028** | **0.004** | **0.011** | **48.132** | **4.0004E-13** |
|  | **rs17050272** | **-0.021** | **0.002** | **-0.016** | **114.047** | **1.1E-28** |
|  | **rs3738951** | **0.014** | **0.002** | **0.010** | **47.922** | **7.7002E-12** |
|  | **rs17323117** | **0.029** | **0.004** | **0.012** | **62.166** | **1.6998E-14** |
|  | **rs2443728** | **-0.014** | **0.003** | **-0.008** | **29.661** | **3.3E-08** |
|  | **rs5398** | **0.023** | **0.002** | **0.016** | **111.107** | **1E-25** |
|  | **rs2193587** | **-0.019** | **0.002** | **-0.012** | **59.302** | **5.6002E-15** |
|  | **rs34631447** | **0.012** | **0.002** | **0.009** | **35.734** | **1.1E-09** |
|  | **rs4678732** | **0.015** | **0.002** | **0.012** | **58.229** | **1.6998E-15** |
|  | **rs4687612** | **0.019** | **0.003** | **0.010** | **45.990** | **2.2999E-12** |
|  | **rs9813894** | **0.030** | **0.003** | **0.017** | **120.657** | **3.7E-29** |
|  | **rs828617** | **-0.017** | **0.002** | **-0.013** | **72.387** | **8.9002E-19** |
|  | **rs4678144** | **0.018** | **0.002** | **0.011** | **52.094** | **1E-13** |
|  | **rs73238159** | **-0.028** | **0.003** | **-0.014** | **88.071** | **4.7995E-21** |
|  | **rs572169** | **0.054** | **0.002** | **0.038** | **639.679** | **1E-144** |
|  | **rs4683324** | **0.020** | **0.002** | **0.015** | **103.335** | **6.2994E-26** |
|  | **rs9859077** | **0.027** | **0.002** | **0.020** | **167.392** | **3.7E-41** |
|  | **rs9833810** | **-0.013** | **0.002** | **-0.010** | **41.150** | **5.7996E-11** |
|  | **rs1042445** | **-0.019** | **0.002** | **-0.012** | **62.495** | **1.5E-16** |
|  | **rs2607775** | **0.016** | **0.002** | **0.012** | **62.720** | **6.0996E-15** |
|  | **rs35041525** | **-0.017** | **0.003** | **-0.009** | **31.538** | **2.8E-08** |
|  | **rs11928797** | **0.027** | **0.003** | **0.014** | **79.979** | **1.2001E-20** |
|  | **rs17074673** | **0.019** | **0.003** | **0.010** | **40.572** | **3.2999E-11** |
|  | **rs2878298** | **0.013** | **0.002** | **0.010** | **39.425** | **3.4002E-11** |
|  | **rs684773** | **0.042** | **0.002** | **0.027** | **329.231** | **7.2996E-78** |
|  | **rs1344672** | **-0.035** | **0.002** | **-0.027** | **313.142** | **3.5003E-71** |
|  | **rs17461749** | **-0.039** | **0.007** | **-0.008** | **28.505** | **3.6E-08** |
|  | **rs6805889** | **-0.013** | **0.002** | **-0.010** | **46.915** | **1.1E-11** |
|  | **rs10935299** | **-0.020** | **0.002** | **-0.014** | **83.816** | **1.2001E-19** |
|  | **rs6798156** | **-0.014** | **0.002** | **-0.010** | **42.011** | **2E-10** |
|  | **rs4234798** | **0.038** | **0.002** | **0.028** | **349.461** | **3.1996E-80** |
|  | **rs1512135** | **-0.023** | **0.003** | **-0.011** | **54.871** | **4.7E-13** |
|  | **rs78659622** | **0.029** | **0.004** | **0.010** | **44.955** | **5.0004E-12** |
|  | **rs4833676** | **0.014** | **0.002** | **0.010** | **43.721** | **2.1999E-11** |
|  | **rs61740705** | **0.019** | **0.002** | **0.012** | **58.449** | **8.1003E-16** |
|  | **rs36023504** | **-0.015** | **0.002** | **-0.011** | **54.560** | **4.1002E-14** |
|  | **rs28551714** | **0.029** | **0.002** | **0.020** | **172.478** | **6.4998E-42** |
|  | **rs6535413** | **-0.014** | **0.002** | **-0.011** | **51.764** | **4.6005E-13** |
|  | **rs813139** | **0.015** | **0.002** | **0.012** | **60.596** | **1.6998E-16** |
|  | **rs7697204** | **0.024** | **0.002** | **0.016** | **114.778** | **8.4996E-29** |
|  | **rs146101385** | **-0.022** | **0.004** | **-0.009** | **33.215** | **4.1E-08** |
|  | **rs3912391** | **-0.029** | **0.002** | **-0.022** | **219.115** | **8.6996E-50** |
|  | **rs1126671** | **0.037** | **0.002** | **0.026** | **295.250** | **3.6E-72** |
|  | **rs41514145** | **0.012** | **0.002** | **0.008** | **27.928** | **4.6E-08** |
|  | **rs362285** | **-0.057** | **0.008** | **-0.011** | **51.565** | **7.1007E-15** |
|  | **rs6447962** | **0.016** | **0.002** | **0.011** | **49.550** | **5.6002E-12** |
|  | **rs2280099** | **0.026** | **0.003** | **0.016** | **105.808** | **1E-26** |
|  | **rs424272** | **0.013** | **0.002** | **0.009** | **32.122** | **2.8E-10** |
|  | **rs6534673** | **0.017** | **0.002** | **0.011** | **57.601** | **7.1007E-15** |
|  | **rs56293839** | **-0.016** | **0.003** | **-0.009** | **34.835** | **4.9E-10** |
|  | **rs2292423** | **-0.012** | **0.002** | **-0.009** | **32.779** | **9.2999E-10** |
|  | **rs976002** | **-0.036** | **0.002** | **-0.024** | **246.705** | **1.5999E-55** |
|  | **rs1902023** | **-0.027** | **0.002** | **-0.020** | **181.454** | **6.7004E-44** |
|  | **rs79880058** | **-0.025** | **0.004** | **-0.009** | **35.173** | **5.1E-10** |
|  | **rs357269** | **-0.014** | **0.003** | **-0.008** | **27.602** | **2.9E-08** |
|  | **rs329120** | **0.017** | **0.002** | **0.013** | **72.793** | **2.3999E-18** |
|  | **rs6879346** | **0.016** | **0.003** | **0.008** | **28.603** | **1.8E-08** |
|  | **rs62372052** | **0.059** | **0.003** | **0.028** | **343.831** | **3.2999E-81** |
|  | **rs985296** | **-0.022** | **0.002** | **-0.016** | **117.987** | **8.6E-30** |
|  | **rs17714046** | **0.038** | **0.005** | **0.011** | **50.189** | **5.9007E-13** |
|  | **rs7723160** | **0.020** | **0.002** | **0.014** | **81.487** | **1.3999E-20** |
|  | **rs9687846** | **0.014** | **0.002** | **0.009** | **33.248** | **6.5999E-10** |
|  | **rs80170948** | **0.036** | **0.005** | **0.011** | **54.356** | **1.5E-14** |
|  | **rs609385** | **-0.021** | **0.002** | **-0.016** | **110.212** | **3.7E-27** |
|  | **rs757647** | **0.039** | **0.002** | **0.024** | **253.411** | **4.1995E-57** |
|  | **rs2895168** | **0.016** | **0.002** | **0.011** | **51.159** | **1.5999E-13** |
|  | **rs76026733** | **0.038** | **0.004** | **0.013** | **75.787** | **5.3003E-19** |
|  | **rs11738977** | **-0.038** | **0.002** | **-0.027** | **325.662** | **5.2E-73** |
|  | **rs4705873** | **-0.038** | **0.003** | **-0.022** | **208.941** | **3.5003E-51** |
|  | **rs12652907** | **0.024** | **0.004** | **0.009** | **38.583** | **4.9E-09** |
|  | **rs532964** | **0.015** | **0.002** | **0.012** | **59.190** | **6.4998E-15** |
|  | **rs6452873** | **0.012** | **0.002** | **0.009** | **32.794** | **3.1E-09** |
|  | **rs34767** | **0.015** | **0.002** | **0.011** | **49.182** | **5.4001E-13** |
|  | **rs4282339** | **-0.051** | **0.002** | **-0.032** | **445.652** | **2E-105** |
|  | **rs9379084** | **0.021** | **0.003** | **0.010** | **46.020** | **3.2999E-12** |
|  | **rs9465733** | **0.012** | **0.002** | **0.008** | **31.451** | **1.1E-08** |
|  | **rs16873583** | **-0.016** | **0.003** | **-0.009** | **35.459** | **8.9E-10** |
|  | **rs6934603** | **-0.029** | **0.002** | **-0.022** | **212.982** | **5.7003E-50** |
|  | **rs7759938** | **0.019** | **0.002** | **0.014** | **81.102** | **6.2001E-21** |
|  | **rs2153960** | **0.052** | **0.002** | **0.036** | **567.356** | **5.297E-129** |
|  | **rs4895842** | **-0.014** | **0.002** | **-0.010** | **44.365** | **1.2999E-11** |
|  | **rs12210951** | **0.016** | **0.002** | **0.011** | **55.815** | **5.1004E-14** |
|  | **rs28399993** | **-0.088** | **0.013** | **-0.010** | **44.197** | **5.4001E-11** |
|  | **rs2235711** | **0.020** | **0.002** | **0.014** | **79.516** | **8.1997E-20** |
|  | **rs1051775** | **-0.015** | **0.002** | **-0.011** | **56.624** | **3.7E-15** |
|  | **rs2023569** | **-0.010** | **0.002** | **-0.007** | **23.331** | **3.7E-08** |
|  | **rs41288985** | **-0.021** | **0.004** | **-0.009** | **31.732** | **2.6E-09** |
|  | **rs2786189** | **-0.012** | **0.002** | **-0.009** | **38.842** | **3.1E-09** |
|  | **rs7742369** | **-0.026** | **0.003** | **-0.015** | **104.521** | **2.1999E-26** |
|  | **rs2395943** | **-0.012** | **0.002** | **-0.009** | **37.003** | **2.6E-10** |
|  | **rs2323034** | **0.036** | **0.002** | **0.027** | **324.525** | **1.1E-74** |
|  | **rs1536241** | **-0.014** | **0.002** | **-0.011** | **49.799** | **1.2001E-12** |
|  | **rs2296198** | **0.014** | **0.002** | **0.009** | **37.088** | **1.8E-09** |
|  | **rs3117234** | **0.014** | **0.003** | **0.009** | **32.275** | **5.1E-09** |
|  | **rs763157** | **-0.022** | **0.003** | **-0.011** | **57.013** | **7.3995E-14** |
|  | **rs13210597** | **0.017** | **0.003** | **0.010** | **47.709** | **1.3999E-11** |
|  | **rs4897242** | **0.016** | **0.003** | **0.009** | **38.642** | **5.6002E-11** |
|  | **rs12208357** | **0.046** | **0.004** | **0.018** | **140.112** | **1.6998E-32** |
|  | **rs668871** | **-0.033** | **0.002** | **-0.026** | **284.681** | **2.0999E-68** |
|  | **rs3008050** | **-0.012** | **0.002** | **-0.009** | **33.264** | **3.5E-09** |
|  | **rs3752416** | **-0.026** | **0.002** | **-0.017** | **129.110** | **1.1E-29** |
|  | **rs1361108** | **0.064** | **0.002** | **0.049** | **1062.094** | **1E-200** |
|  | **rs2504063** | **-0.011** | **0.002** | **-0.009** | **32.132** | **4.5E-08** |
|  | **rs7797661** | **0.013** | **0.002** | **0.010** | **43.021** | **3.6E-11** |
|  | **rs2893449** | **-0.025** | **0.004** | **-0.009** | **34.104** | **6.1E-09** |
|  | **rs700752** | **0.119** | **0.002** | **0.087** | **3327.867** | **1E-200** |
|  | **rs3812316** | **0.037** | **0.003** | **0.019** | **157.235** | **1.9999E-37** |
|  | **rs157934** | **0.058** | **0.002** | **0.041** | **735.615** | **6.501E-166** |
|  | **rs34348385** | **-0.047** | **0.003** | **-0.025** | **276.201** | **2.4998E-64** |
|  | **rs74400046** | **0.064** | **0.008** | **0.012** | **60.335** | **2.3999E-15** |
|  | **rs794364** | **-0.013** | **0.002** | **-0.010** | **42.387** | **1.1E-11** |
|  | **rs2888877** | **0.015** | **0.002** | **0.009** | **37.881** | **7E-10** |
|  | **rs2074684** | **0.022** | **0.003** | **0.011** | **55.224** | **1.5999E-13** |
|  | **rs10229964** | **0.017** | **0.002** | **0.013** | **74.043** | **1.2001E-17** |
|  | **rs798502** | **0.020** | **0.002** | **0.014** | **86.221** | **9.3004E-21** |
|  | **rs2174460** | **-0.016** | **0.002** | **-0.011** | **48.257** | **5.7003E-13** |
|  | **rs34312198** | **0.018** | **0.003** | **0.009** | **31.996** | **6.4E-09** |
|  | **rs13232767** | **-0.013** | **0.002** | **-0.010** | **39.324** | **1.2E-09** |
|  | **rs7789908** | **0.020** | **0.002** | **0.012** | **66.976** | **1.8001E-16** |
|  | **rs10486064** | **0.020** | **0.003** | **0.011** | **49.301** | **6.4998E-12** |
|  | **rs6461071** | **-0.018** | **0.002** | **-0.012** | **67.607** | **3.7E-18** |
|  | **rs13246732** | **0.015** | **0.002** | **0.011** | **53.165** | **1.1E-13** |
|  | **rs17151639** | **0.012** | **0.002** | **0.008** | **29.951** | **3.7E-08** |
|  | **rs4988501** | **-0.021** | **0.002** | **-0.014** | **80.953** | **2.6002E-21** |
|  | **rs1079866** | **0.020** | **0.003** | **0.010** | **46.312** | **6.5993E-13** |
|  | **rs2854746** | **-0.046** | **0.002** | **-0.035** | **533.613** | **1.901E-121** |
|  | **rs6964426** | **0.036** | **0.006** | **0.009** | **34.745** | **3.3E-09** |
|  | **rs1155397** | **0.014** | **0.002** | **0.010** | **44.042** | **2.2999E-11** |
|  | **rs273957** | **-0.023** | **0.002** | **-0.017** | **126.691** | **1.2999E-30** |
|  | **rs445036** | **-0.018** | **0.002** | **-0.012** | **67.716** | **2.2999E-17** |
|  | **rs12550809** | **0.011** | **0.002** | **0.008** | **27.594** | **1.5E-08** |
|  | **rs4073455** | **0.013** | **0.002** | **0.010** | **44.782** | **2.0999E-11** |
|  | **rs1495743** | **-0.028** | **0.002** | **-0.018** | **139.794** | **5.6002E-34** |
|  | **rs4872203** | **0.015** | **0.002** | **0.011** | **48.343** | **1.2999E-12** |
|  | **rs12679822** | **-0.021** | **0.003** | **-0.010** | **45.799** | **2.0999E-11** |
|  | **rs68013747** | **0.020** | **0.002** | **0.014** | **91.124** | **5.5005E-21** |
|  | **rs78357199** | **0.034** | **0.005** | **0.011** | **48.111** | **1.8001E-12** |
|  | **rs2293889** | **-0.026** | **0.002** | **-0.020** | **174.662** | **1.2001E-42** |
|  | **rs9657541** | **-0.018** | **0.003** | **-0.011** | **51.686** | **5.9007E-13** |
|  | **rs1693551** | **-0.014** | **0.002** | **-0.011** | **48.595** | **2.0999E-12** |
|  | **rs16877774** | **-0.017** | **0.002** | **-0.012** | **58.693** | **1.2999E-14** |
|  | **rs2081687** | **-0.027** | **0.002** | **-0.019** | **163.675** | **2.3999E-39** |
|  | **rs1583164** | **0.020** | **0.002** | **0.015** | **100.411** | **3.5003E-24** |
|  | **rs6471133** | **0.028** | **0.002** | **0.017** | **128.484** | **1.1E-29** |
|  | **rs1367628** | **0.018** | **0.003** | **0.011** | **51.599** | **3.9003E-13** |
|  | **rs5899258** | **-0.018** | **0.002** | **-0.013** | **76.546** | **1.6998E-19** |
|  | **rs1270231** | **0.047** | **0.003** | **0.023** | **232.602** | **1.5999E-54** |
|  | **rs10992828** | **0.023** | **0.002** | **0.015** | **102.971** | **1.1E-24** |
|  | **rs56324928** | **0.022** | **0.002** | **0.014** | **82.664** | **7E-21** |
|  | **rs1888223** | **-0.012** | **0.002** | **-0.009** | **33.291** | **1.7E-09** |
|  | **rs28410315** | **0.028** | **0.003** | **0.015** | **104.392** | **1.2999E-26** |
|  | **rs505922** | **0.014** | **0.002** | **0.010** | **40.963** | **3.7E-11** |
|  | **rs466037** | **-0.015** | **0.002** | **-0.011** | **48.944** | **5.7003E-12** |
|  | **rs2274649** | **0.015** | **0.002** | **0.010** | **47.088** | **1.8001E-12** |
|  | **rs11792865** | **0.015** | **0.003** | **0.008** | **28.661** | **4.1E-08** |
|  | **rs10122243** | **-0.015** | **0.002** | **-0.012** | **59.141** | **5.0004E-15** |
|  | **rs4743034** | **0.020** | **0.002** | **0.013** | **72.508** | **4.4005E-18** |
|  | **rs3122934** | **-0.016** | **0.002** | **-0.012** | **61.733** | **9.7994E-16** |
|  | **rs7846925** | **0.022** | **0.003** | **0.011** | **55.182** | **2.4998E-14** |
|  | **rs11794448** | **-0.019** | **0.003** | **-0.010** | **43.720** | **2.3999E-11** |
|  | **rs12253847** | **-0.013** | **0.002** | **-0.010** | **39.593** | **1.7E-10** |
|  | **rs36023044** | **0.022** | **0.003** | **0.012** | **64.815** | **3.1003E-15** |
|  | **rs1832007** | **0.059** | **0.003** | **0.033** | **465.116** | **1.4E-109** |
|  | **rs293281** | **0.012** | **0.002** | **0.008** | **30.609** | **1.7E-09** |
|  | **rs2277222** | **0.016** | **0.002** | **0.011** | **53.003** | **2.4998E-15** |
|  | **rs1171619** | **0.014** | **0.002** | **0.009** | **35.398** | **4.1E-10** |
|  | **rs10786156** | **-0.024** | **0.002** | **-0.018** | **148.854** | **4.4999E-36** |
|  | **rs11197593** | **0.017** | **0.002** | **0.013** | **77.024** | **1.2001E-20** |
|  | **rs12769257** | **-0.020** | **0.003** | **-0.012** | **63.901** | **1.3999E-17** |
|  | **rs2738208** | **0.013** | **0.002** | **0.008** | **30.530** | **1.9E-08** |
|  | **rs10509746** | **0.027** | **0.002** | **0.021** | **188.969** | **1.6998E-45** |
|  | **rs11599690** | **-0.013** | **0.002** | **-0.009** | **37.288** | **1.5E-09** |
|  | **rs7072243** | **0.012** | **0.002** | **0.009** | **35.399** | **3.8E-08** |
|  | **rs1471246** | **0.016** | **0.002** | **0.012** | **66.459** | **3.6E-15** |
|  | **rs55922628** | **-0.012** | **0.002** | **-0.009** | **36.651** | **2E-09** |
|  | **rs3832685** | **-0.016** | **0.002** | **-0.012** | **58.055** | **4.7995E-14** |
|  | **rs78089241** | **0.037** | **0.006** | **0.010** | **45.502** | **1.8001E-11** |
|  | **rs11020842** | **-0.037** | **0.005** | **-0.010** | **47.931** | **1.5999E-12** |
|  | **rs11603496** | **-0.016** | **0.002** | **-0.012** | **63.203** | **2.1999E-16** |
|  | **rs12294104** | **0.022** | **0.003** | **0.013** | **74.136** | **1.2001E-17** |
|  | **rs901105** | **-0.024** | **0.003** | **-0.014** | **82.940** | **1.3999E-20** |
|  | **rs10892919** | **0.015** | **0.002** | **0.012** | **59.893** | **6.2001E-15** |
|  | **rs10835211** | **-0.012** | **0.002** | **-0.008** | **30.193** | **1.9E-08** |
|  | **rs35243581** | **-0.012** | **0.002** | **-0.008** | **30.719** | **1.6E-08** |
|  | **rs3136520** | **-0.040** | **0.007** | **-0.009** | **38.313** | **6.6999E-10** |
|  | **rs174550** | **-0.020** | **0.002** | **-0.015** | **93.255** | **7.7002E-23** |
|  | **rs7952436** | **0.044** | **0.004** | **0.018** | **147.307** | **3.6E-35** |
|  | **rs4938799** | **0.017** | **0.002** | **0.013** | **68.762** | **3.1003E-16** |
|  | **rs6486122** | **0.021** | **0.002** | **0.015** | **99.369** | **3.1996E-23** |
|  | **rs1783826** | **0.011** | **0.002** | **0.008** | **31.192** | **6.5999E-10** |
|  | **rs4245150** | **-0.014** | **0.002** | **-0.011** | **48.146** | **5.1004E-12** |
|  | **rs12364884** | **0.013** | **0.002** | **0.009** | **31.638** | **1.2E-08** |
|  | **rs11042751** | **0.071** | **0.002** | **0.047** | **945.947** | **1E-200** |
|  | **rs10832918** | **-0.021** | **0.002** | **-0.015** | **102.363** | **2.3999E-26** |
|  | **rs2047812** | **-0.041** | **0.003** | **-0.023** | **235.766** | **4.4999E-58** |
|  | **rs2187642** | **-0.022** | **0.002** | **-0.016** | **114.333** | **2.7002E-27** |
|  | **rs66550728** | **0.053** | **0.003** | **0.029** | **368.084** | **1.2999E-83** |
|  | **rs68176600** | **-0.015** | **0.002** | **-0.011** | **49.609** | **5.9007E-13** |
|  | **rs10862960** | **-0.011** | **0.002** | **-0.009** | **32.278** | **8.4E-09** |
|  | **rs4144501** | **0.015** | **0.002** | **0.012** | **61.524** | **2.7002E-14** |
|  | **rs765250** | **0.013** | **0.002** | **0.009** | **36.047** | **2.1E-09** |
|  | **rs2855749** | **-0.011** | **0.002** | **-0.008** | **28.376** | **3.3E-08** |
|  | **rs117380693** | **-0.032** | **0.005** | **-0.009** | **35.699** | **1.3E-09** |
|  | **rs1351394** | **0.026** | **0.002** | **0.020** | **180.583** | **2.4998E-43** |
|  | **rs75218626** | **0.053** | **0.005** | **0.015** | **98.057** | **1.9002E-25** |
|  | **rs1800574** | **0.147** | **0.006** | **0.038** | **624.836** | **1.4E-141** |
|  | **rs2271976** | **-0.024** | **0.003** | **-0.010** | **48.008** | **1.2001E-12** |
|  | **rs861525** | **0.015** | **0.002** | **0.010** | **42.881** | **1.2001E-11** |
|  | **rs2059404** | **0.014** | **0.002** | **0.011** | **51.504** | **1.3999E-12** |
|  | **rs7315980** | **0.020** | **0.004** | **0.009** | **32.709** | **2.3E-08** |
|  | **rs1621686** | **0.020** | **0.002** | **0.014** | **82.775** | **2.6002E-21** |
|  | **rs7489210** | **0.018** | **0.003** | **0.009** | **38.842** | **7.8001E-11** |
|  | **rs10876864** | **0.015** | **0.002** | **0.012** | **57.936** | **1E-14** |
|  | **rs7306772** | **-0.014** | **0.002** | **-0.010** | **42.471** | **2.8E-10** |
|  | **rs249624** | **0.025** | **0.003** | **0.014** | **87.334** | **5.1004E-22** |
|  | **rs11111274** | **-0.079** | **0.002** | **-0.054** | **1278.770** | **1E-200** |
|  | **rs79218426** | **0.073** | **0.005** | **0.022** | **213.750** | **3.7E-51** |
|  | **rs10431392** | **-0.022** | **0.004** | **-0.009** | **33.509** | **0.00000002** |
|  | **rs1678960** | **0.016** | **0.003** | **0.009** | **36.764** | **3.3E-10** |
|  | **rs9533031** | **0.013** | **0.002** | **0.010** | **39.957** | **5.2E-11** |
|  | **rs77107022** | **0.030** | **0.006** | **0.008** | **26.983** | **2.9E-08** |
|  | **rs17428825** | **0.012** | **0.002** | **0.009** | **32.183** | **4.9E-09** |
|  | **rs9534448** | **0.019** | **0.002** | **0.013** | **70.368** | **3.1003E-17** |
|  | **rs76750172** | **0.066** | **0.006** | **0.016** | **104.714** | **1.1E-25** |
|  | **rs61957204** | **0.035** | **0.004** | **0.014** | **88.629** | **8.9002E-22** |
|  | **rs7985107** | **-0.013** | **0.002** | **-0.010** | **44.578** | **8.9002E-12** |
|  | **rs9578326** | **-0.019** | **0.002** | **-0.012** | **61.928** | **4.4999E-15** |
|  | **rs9549092** | **0.040** | **0.003** | **0.024** | **241.281** | **4.9E-58** |
|  | **rs11618980** | **-0.015** | **0.002** | **-0.009** | **39.288** | **4.1E-10** |
|  | **rs9604573** | **0.020** | **0.002** | **0.013** | **77.265** | **1.2001E-17** |
|  | **rs117633128** | **-0.022** | **0.003** | **-0.010** | **47.166** | **3.9003E-11** |
|  | **rs2025258** | **-0.013** | **0.002** | **-0.009** | **37.960** | **3.1E-10** |
|  | **rs747854** | **-0.014** | **0.002** | **-0.010** | **44.779** | **2.4998E-11** |
|  | **rs887506** | **-0.015** | **0.002** | **-0.012** | **57.775** | **1.6998E-14** |
|  | **rs175444** | **-0.018** | **0.002** | **-0.014** | **82.403** | **9.4995E-20** |
|  | **rs112635299** | **-0.075** | **0.007** | **-0.016** | **116.190** | **7.1995E-28** |
|  | **rs8015400** | **0.018** | **0.002** | **0.013** | **69.023** | **4.1995E-17** |
|  | **rs12432100** | **0.022** | **0.002** | **0.015** | **99.972** | **1E-25** |
|  | **rs17577779** | **0.022** | **0.002** | **0.016** | **117.741** | **4.3003E-28** |
|  | **rs33912345** | **-0.023** | **0.002** | **-0.017** | **128.336** | **2.1999E-30** |
|  | **rs60849954** | **-0.025** | **0.004** | **-0.009** | **33.300** | **1.2E-09** |
|  | **rs1956881** | **0.012** | **0.002** | **0.009** | **34.255** | **7.1E-09** |
|  | **rs8017377** | **-0.016** | **0.002** | **-0.012** | **65.768** | **1.5999E-17** |
|  | **rs10146997** | **0.023** | **0.002** | **0.015** | **95.737** | **2.7002E-23** |
|  | **rs10152546** | **0.015** | **0.002** | **0.011** | **53.856** | **2.0999E-13** |
|  | **rs62012820** | **-0.016** | **0.003** | **-0.009** | **38.924** | **2.6E-10** |
|  | **rs7174384** | **-0.039** | **0.004** | **-0.014** | **83.870** | **4.4999E-21** |
|  | **rs150296** | **0.011** | **0.002** | **0.008** | **30.956** | **1.4E-08** |
|  | **rs2414095** | **-0.014** | **0.002** | **-0.010** | **44.211** | **6.0996E-12** |
|  | **rs5742915** | **0.022** | **0.002** | **0.017** | **119.922** | **4.4999E-28** |
|  | **rs8024939** | **0.015** | **0.002** | **0.009** | **36.500** | **2.7E-10** |
|  | **rs2684789** | **-0.016** | **0.002** | **-0.012** | **65.299** | **4.0004E-15** |
|  | **rs11073477** | **0.014** | **0.002** | **0.010** | **43.720** | **3.3E-10** |
|  | **rs55707100** | **-0.146** | **0.006** | **-0.035** | **537.780** | **1.799E-123** |
|  | **rs7178424** | **-0.038** | **0.002** | **-0.029** | **359.117** | **1.2999E-85** |
|  | **rs12912439** | **0.018** | **0.002** | **0.013** | **70.120** | **1.5999E-17** |
|  | **rs8028620** | **0.019** | **0.002** | **0.015** | **93.709** | **3.6E-23** |
|  | **rs113439442** | **-0.043** | **0.004** | **-0.016** | **109.917** | **1.2999E-25** |
|  | **rs7197580** | **-0.012** | **0.002** | **-0.009** | **36.693** | **3.4E-10** |
|  | **rs72793809** | **0.017** | **0.002** | **0.013** | **74.944** | **1.9002E-18** |
|  | **rs66720010** | **-0.024** | **0.002** | **-0.018** | **142.365** | **4.7E-33** |
|  | **rs1369924** | **-0.028** | **0.003** | **-0.017** | **122.994** | **1.9002E-29** |
|  | **rs17616063** | **-0.027** | **0.004** | **-0.011** | **52.992** | **1.2999E-13** |
|  | **rs1984470** | **0.013** | **0.002** | **0.010** | **42.304** | **4.7E-11** |
|  | **rs898962** | **-0.013** | **0.002** | **-0.010** | **43.140** | **6.7004E-12** |
|  | **rs4888156** | **0.030** | **0.002** | **0.022** | **205.866** | **2.2999E-47** |
|  | **rs4988483** | **-0.159** | **0.004** | **-0.056** | **1345.715** | **1E-200** |
|  | **rs118073883** | **-0.030** | **0.005** | **-0.009** | **36.187** | **1E-09** |
|  | **rs61742747** | **-0.077** | **0.006** | **-0.019** | **152.134** | **1.6998E-34** |
|  | **rs6498697** | **-0.015** | **0.002** | **-0.011** | **55.535** | **1.9002E-14** |
|  | **rs62052820** | **-0.025** | **0.002** | **-0.016** | **108.861** | **1.9999E-24** |
|  | **rs11862755** | **0.089** | **0.004** | **0.035** | **539.128** | **5.998E-123** |
|  | **rs3809627** | **-0.016** | **0.002** | **-0.012** | **66.233** | **1.9002E-16** |
|  | **rs73530203** | **-0.031** | **0.002** | **-0.022** | **215.642** | **3.4002E-51** |
|  | **rs78842967** | **-0.023** | **0.003** | **-0.011** | **49.458** | **8.1003E-12** |
|  | **rs727428** | **0.018** | **0.002** | **0.014** | **84.814** | **2.1999E-20** |
|  | **rs8068844** | **-0.015** | **0.002** | **-0.011** | **48.869** | **2.2999E-13** |
|  | **rs1574525** | **-0.015** | **0.002** | **-0.010** | **46.147** | **1.6998E-11** |
|  | **rs397969** | **-0.012** | **0.002** | **-0.009** | **34.842** | **2.1E-09** |
|  | **rs8078723** | **-0.024** | **0.002** | **-0.018** | **138.767** | **4.1995E-34** |
|  | **rs17585974** | **0.019** | **0.003** | **0.011** | **51.079** | **1.2999E-13** |
|  | **rs55714927** | **0.020** | **0.003** | **0.012** | **60.902** | **5.4001E-16** |
|  | **rs8065258** | **-0.018** | **0.002** | **-0.011** | **50.155** | **2.1999E-13** |
|  | **rs2665799** | **-0.030** | **0.002** | **-0.021** | **194.498** | **6.7999E-46** |
|  | **rs77542162** | **0.044** | **0.007** | **0.010** | **41.917** | **8.1997E-12** |
|  | **rs8076897** | **0.014** | **0.002** | **0.010** | **46.734** | **5.6002E-11** |
|  | **rs56206519** | **0.011** | **0.002** | **0.008** | **30.233** | **2.4E-08** |
|  | **rs2230316** | **-0.020** | **0.002** | **-0.015** | **92.410** | **1.5E-22** |
|  | **rs72631343** | **-0.018** | **0.003** | **-0.009** | **37.390** | **1.8E-09** |
|  | **rs1047743** | **-0.017** | **0.002** | **-0.012** | **62.056** | **1.2001E-15** |
|  | **rs2189336** | **0.015** | **0.002** | **0.011** | **55.050** | **6.7004E-14** |
|  | **rs76708468** | **0.089** | **0.005** | **0.026** | **303.440** | **1.2001E-69** |
|  | **rs36000545** | **-0.018** | **0.002** | **-0.013** | **74.201** | **2.4998E-19** |
|  | **rs3850805** | **0.014** | **0.002** | **0.009** | **38.521** | **2.9E-11** |
|  | **rs1942583** | **0.016** | **0.002** | **0.012** | **61.814** | **5.2E-15** |
|  | **rs4468717** | **-0.028** | **0.004** | **-0.011** | **50.709** | **5.7996E-14** |
|  | **rs34233878** | **0.024** | **0.003** | **0.011** | **53.179** | **7.5998E-13** |
|  | **rs585187** | **0.015** | **0.002** | **0.012** | **59.509** | **1.5E-14** |
|  | **rs2198557** | **0.019** | **0.002** | **0.013** | **76.820** | **7.1995E-19** |
|  | **rs35740221** | **-0.012** | **0.002** | **-0.009** | **32.390** | **1.4E-08** |
|  | **rs77169818** | **-0.059** | **0.005** | **-0.018** | **139.627** | **1.3999E-32** |
|  | **rs16948305** | **-0.016** | **0.003** | **-0.009** | **36.529** | **3.5E-10** |
|  | **rs595657** | **-0.012** | **0.002** | **-0.008** | **31.458** | **1.1E-08** |
|  | **rs17487484** | **-0.014** | **0.002** | **-0.010** | **47.561** | **4.1995E-11** |
|  | **rs12454712** | **-0.016** | **0.002** | **-0.012** | **61.323** | **1.5999E-15** |
|  | **rs3745683** | **0.028** | **0.004** | **0.011** | **53.465** | **7.2996E-13** |
|  | **rs10426201** | **-0.024** | **0.003** | **-0.014** | **82.747** | **9.0991E-20** |
|  | **rs8105174** | **-0.049** | **0.003** | **-0.029** | **372.671** | **6.5993E-85** |
|  | **rs34536443** | **-0.054** | **0.005** | **-0.017** | **125.375** | **2.8003E-29** |
|  | **rs6510033** | **-0.018** | **0.002** | **-0.013** | **69.798** | **6.4998E-19** |
|  | **rs75403087** | **-0.023** | **0.003** | **-0.010** | **46.018** | **1.1E-12** |
|  | **rs12975366** | **-0.022** | **0.002** | **-0.016** | **106.374** | **1.1E-24** |
|  | **rs632616** | **-0.040** | **0.002** | **-0.028** | **347.471** | **2.8003E-84** |
|  | **rs6510959** | **-0.020** | **0.003** | **-0.011** | **54.357** | **7.5007E-12** |
|  | **rs2288842** | **-0.018** | **0.002** | **-0.012** | **67.409** | **2.8003E-18** |
|  | **rs8104929** | **0.012** | **0.002** | **0.009** | **32.615** | **7.9001E-09** |
|  | **rs838133** | **0.028** | **0.002** | **0.021** | **185.519** | **2.0999E-44** |
|  | **rs60789653** | **-0.026** | **0.003** | **-0.015** | **97.901** | **3.1003E-23** |
|  | **rs2738787** | **-0.032** | **0.004** | **-0.013** | **76.189** | **9.0991E-19** |
|  | **rs7261425** | **-0.051** | **0.002** | **-0.035** | **530.447** | **4.603E-125** |
|  | **rs6082354** | **-0.045** | **0.002** | **-0.032** | **454.969** | **1.799E-106** |
|  | **rs8121509** | **0.027** | **0.002** | **0.021** | **189.978** | **1.6998E-44** |
|  | **rs56344540** | **-0.045** | **0.004** | **-0.019** | **156.117** | **2.7002E-37** |
|  | **rs6037513** | **0.013** | **0.002** | **0.010** | **44.469** | **1.1E-11** |
|  | **rs224331** | **-0.015** | **0.002** | **-0.011** | **50.200** | **1.3999E-13** |
|  | **rs867264** | **0.013** | **0.002** | **0.010** | **43.594** | **5.4001E-11** |
|  | **rs6127684** | **0.018** | **0.003** | **0.010** | **41.212** | **4.0004E-11** |
|  | **rs11088472** | **0.017** | **0.002** | **0.013** | **70.578** | **2.1999E-18** |
|  | **rs364199** | **0.015** | **0.002** | **0.011** | **51.000** | **3.9003E-12** |
|  | **rs17274750** | **-0.019** | **0.003** | **-0.009** | **31.676** | **2.2E-08** |
|  | **rs762360** | **0.041** | **0.002** | **0.029** | **357.861** | **6.2994E-83** |
|  | **rs181362** | **-0.015** | **0.003** | **-0.009** | **35.955** | **7.7E-10** |
|  | **rs6519133** | **-0.029** | **0.002** | **-0.022** | **210.304** | **5.3003E-47** |
|  | **rs2234059** | **-0.032** | **0.002** | **-0.021** | **184.988** | **5.6002E-45** |
|  | **rs5758896** | **0.011** | **0.002** | **0.008** | **28.803** | **1.5E-08** |
|  | **rs5751777** | **-0.018** | **0.002** | **-0.014** | **81.683** | **3.6E-20** |
|  | **rs5750202** | **0.027** | **0.003** | **0.014** | **84.829** | **9.7006E-22** |
|  | **rs2294239** | **-0.014** | **0.002** | **-0.011** | **49.100** | **2.2999E-12** |
| IGF-1R |  |  |  |  |  |  |
|  | **rs635634** | **0.2179** | **0.032** | **-0.117** | **46.079** | **1.14815E-11** |
| IGF-2R |  |  |  |  |  |  |
|  | **rs12213092** | **0.751** | **0.134** | **0.175** | **31.181** | **3.03103E-08** |
|  | **rs600324** | **0.982** | **0.064** | **0.438** | **235.623** | **7.10886E-48** |
| IGFBP-3 |  |  |  |  |  |  |
|  | **rs2949833** | **0.428** | **0.043** | **0.302** | **99.102** | **2.57988E-22** |
| IGFBP-7 |  |  |  |  |  |  |
|  | **rs2412776** | **-0.443** | **0.052** | **-0.261** | **72.710** | **5.5386E-17** |
