# Supplementary material for: Causal associations between the insulin-like growth factor family and sarcopenia: a bidirectional Mendelian randomization study
Source: Front Endocrinol (Lausanne). 2024 Oct 23;15:1422472. doi: 10.3389/fendo.2024.1422472 (PMC11537870; doi:10.3389/fendo.2024.1422472)
Supplement: Supplementary Table 3 — List of SNPS associated with sarcopenia. [file Table3.docx]

|  | SNP | Beta | Se | R | Fscore | Pvalue |
| --- | --- | --- | --- | --- | --- | --- |
| Low hand grip strength (60 years and older) |  |  |  |  |  |  |
|  | **rs12140813** | **0.051** | **0.009** | **0.011** | **29.552** | **4.763E-08** |
|  | **rs958685** | **-0.043** | **0.007** | **-0.011** | **33.452** | **6.5179E-09** |
|  | **rs7624084** | **-0.043** | **0.007** | **-0.011** | **33.452** | **8.5071E-09** |
|  | **rs13107325** | **0.090** | **0.014** | **0.013** | **42.250** | **7.4182E-11** |
|  | **rs34415150** | **0.083** | **0.010** | **0.017** | **70.798** | **4.4218E-17** |
|  | **rs185320691** | **0.091** | **0.015** | **0.012** | **39.105** | **3.844E-10** |
|  | **rs10952289** | **-0.044** | **0.008** | **-0.011** | **31.102** | **2.104E-08** |
|  | **rs11236213** | **-0.050** | **0.008** | **-0.012** | **39.690** | **3.011E-10** |
|  | **rs10846071** | **0.043** | **0.008** | **0.011** | **33.331** | **7.324E-09** |
|  | **rs34464763** | **0.054** | **0.009** | **0.012** | **40.013** | **3.152E-10** |
|  | **rs3118903** | **0.058** | **0.009** | **0.013** | **42.694** | **6.7143E-11** |
|  | **rs2899611** | **0.043** | **0.007** | **0.011** | **33.923** | **6.014E-09** |
|  | **rs8061064** | **0.041** | **0.007** | **0.011** | **30.250** | **3.546E-08** |
|  | **rs143459567** | **0.119** | **0.019** | **0.012** | **39.311** | **3.405E-10** |
|  | **rs62102286** | **-0.049** | **0.007** | **-0.013** | **43.311** | **5.4891E-11** |
|  | **rs79723785** | **0.167** | **0.029** | **0.011** | **32.642** | **1.159E-08** |
|  | **rs143384** | **-0.055** | **0.008** | **-0.014** | **52.804** | **4.473E-13** |
|  |  |  |  |  |  |  |
| Appendicular lean mass |  |  |  |  |  |  |
|  | **rs200439** | **-0.013** | **0.002** | **-0.008** | **30.972** | **1.51E-08** |
|  | **rs2807339** | **0.016** | **0.002** | **0.011** | **54.223** | **1.2399E-13** |
|  | **rs150188352** | **0.031** | **0.002** | **0.019** | **161.502** | **8.4275E-38** |
|  | **rs60804050** | **-0.022** | **0.002** | **-0.015** | **106.778** | **5.013E-24** |
|  | **rs2025808** | **0.012** | **0.002** | **0.008** | **30.752** | **1.716E-08** |
|  | **rs6693481** | **-0.014** | **0.002** | **-0.011** | **51.123** | **2.208E-12** |
|  | **rs61827272** | **0.014** | **0.002** | **0.010** | **47.020** | **7.8904E-12** |
|  | **rs12724708** | **0.024** | **0.002** | **0.018** | **147.623** | **1.6562E-35** |
|  | **rs3033487** | **0.015** | **0.002** | **0.010** | **40.641** | **1.934E-10** |
|  | **rs11260623** | **0.012** | **0.002** | **0.009** | **37.920** | **4.856E-10** |
|  | **rs301807** | **-0.014** | **0.002** | **-0.011** | **57.440** | **2.5258E-14** |
|  | **rs212526** | **0.021** | **0.002** | **0.017** | **126.859** | **3.8433E-29** |
|  | **rs7522400** | **0.013** | **0.002** | **0.009** | **34.382** | **5.625E-09** |
|  | **rs670318** | **0.041** | **0.004** | **0.014** | **88.104** | **2.52E-21** |
|  | **rs2025609** | **0.019** | **0.003** | **0.011** | **51.178** | **2.0022E-12** |
|  | **rs7543202** | **0.013** | **0.002** | **0.010** | **46.097** | **2.8648E-11** |
|  | **rs11590254** | **0.019** | **0.002** | **0.014** | **86.490** | **4.3361E-20** |
|  | **rs34654458** | **-0.019** | **0.002** | **-0.015** | **101.055** | **5.7174E-24** |
|  | **rs4644481** | **-0.011** | **0.002** | **-0.009** | **34.748** | **3.542E-09** |
|  | **rs7367519** | **0.016** | **0.002** | **0.012** | **67.240** | **4.6806E-16** |
|  | **rs951366** | **0.021** | **0.002** | **0.016** | **116.413** | **9.1538E-27** |
|  | **rs7418410** | **0.016** | **0.002** | **0.012** | **66.551** | **5.6364E-16** |
|  | **rs7543136** | **-0.021** | **0.002** | **-0.015** | **100.000** | **9.9586E-24** |
|  | **rs4360494** | **-0.020** | **0.002** | **-0.016** | **108.598** | **7.8832E-26** |
|  | **rs55717234** | **0.012** | **0.002** | **0.010** | **41.230** | **1.341E-10** |
|  | **rs11580040** | **0.033** | **0.004** | **0.014** | **86.224** | **6.7608E-21** |
|  | **rs2209098** | **0.024** | **0.002** | **0.018** | **144.000** | **1.7278E-32** |
|  | **rs17278379** | **0.023** | **0.003** | **0.012** | **60.732** | **2.3977E-15** |
|  | **rs66579625** | **0.018** | **0.002** | **0.014** | **87.767** | **8.9105E-21** |
|  | **rs4655345** | **-0.025** | **0.002** | **-0.019** | **167.634** | **5.8103E-38** |
|  | **rs1797070** | **0.022** | **0.002** | **0.016** | **108.755** | **4.9329E-25** |
|  | **rs12563442** | **0.012** | **0.002** | **0.009** | **33.751** | **9.933E-09** |
|  | **rs80295797** | **-0.020** | **0.002** | **-0.015** | **98.010** | **3.8256E-23** |
|  | **rs4274112** | **-0.022** | **0.002** | **-0.016** | **117.723** | **2.4712E-28** |
|  | **rs113107560** | **-0.019** | **0.002** | **-0.015** | **100.000** | **6.9855E-23** |
|  | **rs11210892** | **0.012** | **0.002** | **0.009** | **34.810** | **3.57E-09** |
|  | **rs12074850** | **0.039** | **0.003** | **0.018** | **141.826** | **2.7221E-33** |
|  | **rs1514134** | **-0.011** | **0.002** | **-0.009** | **36.000** | **3.574E-09** |
|  | **rs34517439** | **0.042** | **0.003** | **0.022** | **210.750** | **5.8023E-48** |
|  | **rs10922475** | **0.016** | **0.002** | **0.012** | **70.030** | **2.1742E-17** |
|  | **rs3768495** | **-0.018** | **0.002** | **-0.013** | **71.846** | **1.0661E-17** |
|  | **rs28736838** | **-0.012** | **0.002** | **-0.009** | **34.223** | **1.07E-08** |
|  | **rs200091076** | **-0.012** | **0.002** | **-0.009** | **34.223** | **6.9241E-09** |
|  | **rs905938** | **0.039** | **0.002** | **0.028** | **352.009** | **8.4256E-77** |
|  | **rs6675858** | **-0.014** | **0.002** | **-0.009** | **35.480** | **2.404E-09** |
|  | **rs2789365** | **-0.015** | **0.002** | **-0.011** | **58.241** | **1.1421E-14** |
|  | **rs377599** | **0.022** | **0.002** | **0.017** | **130.440** | **3.4987E-29** |
|  | **rs11121615** | **-0.020** | **0.002** | **-0.015** | **102.010** | **3.3189E-23** |
|  | **rs2791654** | **-0.024** | **0.002** | **-0.016** | **118.019** | **1.2059E-28** |
|  | **rs6425817** | **0.016** | **0.002** | **0.012** | **61.623** | **2.9847E-15** |
|  | **rs2885697** | **-0.032** | **0.002** | **-0.024** | **260.823** | **9.2066E-60** |
|  | **rs4847378** | **0.014** | **0.002** | **0.011** | **51.235** | **1.6429E-12** |
|  | **rs1405227** | **0.013** | **0.002** | **0.010** | **41.603** | **1.573E-10** |
|  | **rs1325596** | **0.029** | **0.002** | **0.023** | **228.169** | **2.7708E-52** |
|  | **rs200348453** | **0.018** | **0.002** | **0.014** | **89.751** | **7.8813E-21** |
|  | **rs234640** | **-0.013** | **0.002** | **-0.010** | **47.537** | **3.8681E-12** |
|  | **rs1005723** | **0.016** | **0.002** | **0.010** | **45.002** | **1.7869E-11** |
|  | **rs10171272** | **0.014** | **0.002** | **0.010** | **46.240** | **2.8721E-11** |
|  | **rs17681189** | **-0.013** | **0.002** | **-0.010** | **47.537** | **5.8197E-12** |
|  | **rs76517946** | **-0.037** | **0.004** | **-0.016** | **110.550** | **1.694E-26** |
|  | **rs867529** | **0.018** | **0.002** | **0.013** | **76.771** | **1.0009E-18** |
|  | **rs6543146** | **0.015** | **0.002** | **0.012** | **65.695** | **4.187E-16** |
|  | **rs6738207** | **0.013** | **0.002** | **0.010** | **44.679** | **4.1448E-11** |
|  | **rs71414738** | **0.015** | **0.003** | **0.009** | **36.000** | **1.002E-09** |
|  | **rs2390669** | **0.017** | **0.003** | **0.009** | **38.617** | **6.996E-10** |
|  | **rs13430869** | **0.027** | **0.002** | **0.019** | **167.764** | **6.3665E-37** |
|  | **rs17408561** | **0.012** | **0.002** | **0.009** | **37.823** | **6E-10** |
|  | **rs17246129** | **0.025** | **0.002** | **0.019** | **161.290** | **1.2691E-35** |
|  | **rs2305141** | **0.018** | **0.002** | **0.014** | **92.767** | **1.075E-21** |
|  | **rs2971857** | **-0.012** | **0.002** | **-0.009** | **39.227** | **3.7E-10** |
|  | **rs10203320** | **0.014** | **0.002** | **0.010** | **47.610** | **7.856E-12** |
|  | **rs144627572** | **0.044** | **0.005** | **0.012** | **68.608** | **1.302E-16** |
|  | **rs1260326** | **0.032** | **0.002** | **0.025** | **289.000** | **6.1589E-64** |
|  | **rs650508** | **-0.013** | **0.002** | **-0.010** | **42.250** | **1.861E-10** |
|  | **rs60142646** | **-0.024** | **0.004** | **-0.008** | **31.842** | **2.49E-08** |
|  | **rs75022676** | **-0.016** | **0.002** | **-0.011** | **50.225** | **2.8432E-12** |
|  | **rs199647708** | **0.011** | **0.002** | **0.009** | **36.000** | **3.433E-09** |
|  | **rs17400325** | **0.035** | **0.005** | **0.011** | **53.882** | **2.1009E-13** |
|  | **rs1035583** | **0.015** | **0.002** | **0.012** | **60.676** | **1.9921E-14** |
|  | **rs7598430** | **-0.016** | **0.002** | **-0.013** | **70.914** | **1.3709E-17** |
|  | **rs10202701** | **0.023** | **0.002** | **0.018** | **142.740** | **3.1067E-33** |
|  | **rs10205141** | **0.024** | **0.004** | **0.008** | **30.001** | **4.71E-08** |
|  | **rs10203386** | **-0.024** | **0.002** | **-0.019** | **156.909** | **1.7458E-36** |
|  | **rs10202845** | **-0.029** | **0.003** | **-0.014** | **92.160** | **5.3456E-22** |
|  | **rs67716382** | **0.023** | **0.002** | **0.015** | **96.552** | **1.6508E-23** |
|  | **rs2717008** | **-0.013** | **0.002** | **-0.010** | **44.679** | **4.9877E-11** |
|  | **rs4852257** | **-0.023** | **0.002** | **-0.018** | **147.814** | **6.2116E-34** |
|  | **rs201570119** | **0.019** | **0.002** | **0.013** | **71.146** | **5.4765E-17** |
|  | **rs35223841** | **0.011** | **0.002** | **0.008** | **31.125** | **4.247E-08** |
|  | **rs55852614** | **-0.039** | **0.002** | **-0.027** | **319.110** | **3.287E-73** |
|  | **rs3063063** | **-0.018** | **0.002** | **-0.014** | **82.810** | **2.5669E-19** |
|  | **rs144343497** | **-0.013** | **0.002** | **-0.009** | **33.943** | **5.059E-09** |
|  | **rs700677** | **0.017** | **0.002** | **0.013** | **74.823** | **1.1301E-18** |
|  | **rs12997625** | **-0.017** | **0.002** | **-0.013** | **80.055** | **1.5011E-19** |
|  | **rs3116194** | **-0.030** | **0.003** | **-0.014** | **84.985** | **8.2947E-21** |
|  | **rs7570235** | **-0.017** | **0.002** | **-0.013** | **78.183** | **2.0831E-18** |
|  | **rs7563362** | **0.035** | **0.003** | **0.019** | **169.964** | **3.2712E-39** |
|  | **rs6721191** | **-0.014** | **0.002** | **-0.011** | **57.440** | **3.1703E-14** |
|  | **rs12713004** | **0.037** | **0.002** | **0.026** | **305.417** | **2.3983E-68** |
|  | **rs6739278** | **-0.021** | **0.002** | **-0.013** | **76.563** | **1.2729E-18** |
|  | **rs2347603** | **-0.018** | **0.002** | **-0.012** | **67.688** | **5.6455E-17** |
|  | **rs702886** | **0.012** | **0.002** | **0.009** | **36.000** | **1.112E-09** |
|  | **rs55980611** | **0.016** | **0.003** | **0.009** | **34.306** | **4.217E-09** |
|  | **rs14976** | **0.014** | **0.002** | **0.011** | **51.840** | **1.4269E-12** |
|  | **rs72809820** | **-0.011** | **0.002** | **-0.008** | **30.803** | **3.133E-08** |
|  | **rs9636364** | **0.011** | **0.002** | **0.009** | **33.518** | **5.093E-09** |
|  | **rs10864899** | **-0.011** | **0.002** | **-0.009** | **34.748** | **3.845E-09** |
|  | **rs488621** | **0.019** | **0.002** | **0.015** | **101.055** | **2.8642E-24** |
|  | **rs2138374** | **-0.015** | **0.002** | **-0.011** | **55.503** | **2.79E-13** |
|  | **rs10221831** | **0.030** | **0.005** | **0.008** | **32.040** | **1.803E-08** |
|  | **rs17773965** | **-0.016** | **0.003** | **-0.009** | **36.446** | **1.507E-09** |
|  | **rs62106258** | **-0.050** | **0.004** | **-0.017** | **131.207** | **6.4476E-31** |
|  | **rs3769598** | **0.017** | **0.003** | **0.009** | **40.111** | **1.322E-10** |
|  | **rs202098543** | **0.013** | **0.002** | **0.010** | **43.560** | **1.5599E-11** |
|  | **rs59985551** | **-0.031** | **0.002** | **-0.021** | **202.415** | **2.4339E-44** |
|  | **rs62143873** | **-0.012** | **0.002** | **-0.009** | **36.634** | **1.193E-09** |
|  | **rs12616192** | **-0.026** | **0.004** | **-0.010** | **47.175** | **6.8297E-12** |
|  | **rs61397287** | **0.024** | **0.004** | **0.010** | **42.612** | **4.4535E-11** |
|  | **rs13391980** | **-0.023** | **0.003** | **-0.012** | **60.196** | **7.598E-15** |
|  | **rs34788019** | **-0.014** | **0.002** | **-0.011** | **53.521** | **2.8418E-13** |
|  | **rs1047891** | **0.023** | **0.002** | **0.017** | **135.723** | **5.7043E-31** |
|  | **rs1478575** | **0.031** | **0.002** | **0.023** | **243.360** | **5.2024E-54** |
|  | **rs11684531** | **-0.017** | **0.003** | **-0.009** | **37.735** | **4.167E-10** |
|  | **rs1899040** | **0.015** | **0.002** | **0.010** | **43.675** | **9.0407E-11** |
|  | **rs2270894** | **-0.033** | **0.002** | **-0.021** | **191.361** | **1.254E-42** |
|  | **rs113671109** | **-0.015** | **0.002** | **-0.010** | **42.533** | **4.2286E-11** |
|  | **rs6789000** | **0.012** | **0.002** | **0.009** | **36.603** | **1.611E-09** |
|  | **rs4504126** | **0.046** | **0.006** | **0.012** | **62.901** | **1.6159E-15** |
|  | **rs140440099** | **0.061** | **0.006** | **0.014** | **94.676** | **1.4461E-22** |
|  | **rs17718736** | **0.012** | **0.002** | **0.009** | **33.063** | **1.394E-08** |
|  | **rs4682483** | **-0.017** | **0.003** | **-0.009** | **40.274** | **2.651E-10** |
|  | **rs4683435** | **0.014** | **0.002** | **0.010** | **42.843** | **1.605E-10** |
|  | **rs900399** | **0.016** | **0.002** | **0.013** | **74.504** | **1.3539E-17** |
|  | **rs1290786** | **-0.014** | **0.002** | **-0.011** | **56.645** | **7.1417E-14** |
|  | **rs9647379** | **0.022** | **0.002** | **0.017** | **128.047** | **5.5475E-29** |
|  | **rs2194411** | **0.044** | **0.003** | **0.023** | **233.352** | **2.4339E-54** |
|  | **rs11720869** | **0.014** | **0.002** | **0.011** | **49.703** | **2.5381E-12** |
|  | **rs73052033** | **-0.015** | **0.002** | **-0.009** | **39.585** | **4.7901E-10** |
|  | **rs336630** | **-0.011** | **0.002** | **-0.008** | **31.125** | **2.897E-08** |
|  | **rs9838614** | **-0.019** | **0.002** | **-0.015** | **94.806** | **1.2109E-21** |
|  | **rs6762851** | **-0.022** | **0.002** | **-0.016** | **118.810** | **1.4969E-28** |
|  | **rs182798714** | **0.038** | **0.006** | **0.009** | **36.778** | **1.451E-09** |
|  | **rs591668** | **-0.017** | **0.002** | **-0.014** | **83.867** | **1.9962E-19** |
|  | **rs36012032** | **0.030** | **0.003** | **0.013** | **81.546** | **9.9312E-20** |
|  | **rs839255** | **-0.013** | **0.002** | **-0.009** | **36.000** | **8.3821E-10** |
|  | **rs9809116** | **-0.016** | **0.002** | **-0.013** | **70.914** | **1.3131E-16** |
|  | **rs7633464** | **0.018** | **0.002** | **0.014** | **84.834** | **1.275E-20** |
|  | **rs115010283** | **0.034** | **0.002** | **0.025** | **289.000** | **2.3518E-63** |
|  | **rs61732778** | **0.023** | **0.004** | **0.009** | **38.641** | **3.132E-10** |
|  | **rs4076108** | **0.017** | **0.002** | **0.012** | **62.554** | **2.2972E-15** |
|  | **rs34312629** | **-0.017** | **0.002** | **-0.012** | **65.533** | **2.1232E-15** |
|  | **rs200739311** | **-0.013** | **0.002** | **-0.010** | **40.960** | **1.553E-10** |
|  | **rs544136** | **0.012** | **0.002** | **0.008** | **30.250** | **2.501E-08** |
|  | **rs4073154** | **0.027** | **0.002** | **0.018** | **141.921** | **1.9249E-33** |
|  | **rs2871960** | **0.047** | **0.002** | **0.037** | **609.310** | **2.168E-135** |
|  | **rs1730028** | **0.013** | **0.002** | **0.010** | **47.537** | **7.3858E-12** |
|  | **rs71635721** | **0.032** | **0.004** | **0.012** | **65.652** | **3.4642E-16** |
|  | **rs34390533** | **-0.026** | **0.002** | **-0.017** | **136.465** | **6.6911E-32** |
|  | **rs7610055** | **-0.037** | **0.003** | **-0.019** | **165.433** | **3.553E-38** |
|  | **rs56239180** | **-0.046** | **0.006** | **-0.011** | **54.808** | **1.851E-13** |
|  | **rs9828525** | **0.012** | **0.002** | **0.009** | **40.557** | **2.508E-10** |
|  | **rs116493405** | **0.029** | **0.004** | **0.010** | **46.694** | **9.5214E-12** |
|  | **rs9832919** | **-0.018** | **0.002** | **-0.013** | **80.103** | **7.8959E-20** |
|  | **rs1823217** | **-0.018** | **0.002** | **-0.013** | **81.903** | **4.056E-20** |
|  | **rs11461979** | **0.014** | **0.002** | **0.010** | **42.250** | **9.7972E-11** |
|  | **rs113289555** | **-0.021** | **0.002** | **-0.013** | **80.219** | **7.3266E-20** |
|  | **rs13127468** | **-0.012** | **0.002** | **-0.010** | **41.909** | **9.8583E-11** |
|  | **rs10005035** | **-0.018** | **0.002** | **-0.012** | **69.444** | **6.5133E-17** |
|  | **rs1472852** | **-0.064** | **0.003** | **-0.037** | **602.136** | **8.222E-135** |
|  | **rs963317** | **-0.014** | **0.002** | **-0.010** | **46.240** | **1.0311E-11** |
|  | **rs781669** | **0.016** | **0.002** | **0.013** | **74.504** | **3.1412E-18** |
|  | **rs13103161** | **-0.028** | **0.002** | **-0.022** | **223.424** | **2.3768E-48** |
|  | **rs6849302** | **0.016** | **0.002** | **0.010** | **41.710** | **7.1072E-11** |
|  | **rs145126099** | **-0.013** | **0.002** | **-0.010** | **43.978** | **3.2893E-11** |
|  | **rs1443536** | **0.022** | **0.002** | **0.015** | **107.764** | **1.9121E-26** |
|  | **rs72657800** | **-0.022** | **0.004** | **-0.009** | **39.152** | **6.391E-10** |
|  | **rs11098677** | **-0.026** | **0.002** | **-0.017** | **130.754** | **3.9373E-30** |
|  | **rs12512942** | **-0.016** | **0.002** | **-0.012** | **65.610** | **1.5802E-16** |
|  | **rs59950280** | **-0.025** | **0.002** | **-0.019** | **161.290** | **7.3198E-36** |
|  | **rs10019221** | **-0.012** | **0.002** | **-0.010** | **42.593** | **1.221E-10** |
|  | **rs36052389** | **0.013** | **0.002** | **0.010** | **48.266** | **5.2674E-12** |
|  | **rs3103223** | **0.013** | **0.002** | **0.009** | **32.802** | **6.018E-09** |
|  | **rs139921635** | **0.039** | **0.006** | **0.009** | **38.560** | **6.156E-10** |
|  | **rs111612346** | **-0.014** | **0.002** | **-0.010** | **47.610** | **4.2511E-12** |
|  | **rs34548509** | **0.012** | **0.002** | **0.009** | **34.810** | **3.197E-09** |
|  | **rs13123591** | **0.019** | **0.002** | **0.014** | **85.563** | **2.3529E-20** |
|  | **rs6821305** | **0.020** | **0.002** | **0.016** | **115.280** | **3.1463E-26** |
|  | **rs72695791** | **-0.030** | **0.005** | **-0.009** | **33.913** | **4.576E-09** |
|  | **rs2324154** | **0.015** | **0.002** | **0.012** | **62.327** | **1.916E-15** |
|  | **rs116339650** | **-0.018** | **0.003** | **-0.009** | **36.415** | **1.052E-09** |
|  | **rs2303423** | **0.017** | **0.003** | **0.008** | **31.360** | **2.644E-08** |
|  | **rs13109280** | **0.013** | **0.002** | **0.010** | **42.903** | **9.1453E-11** |
|  | **rs116052377** | **0.023** | **0.004** | **0.010** | **41.327** | **7.8904E-11** |
|  | **rs7689420** | **0.047** | **0.003** | **0.028** | **347.450** | **1.4959E-76** |
|  | **rs11721522** | **0.011** | **0.002** | **0.008** | **31.125** | **4.0301E-08** |
|  | **rs73856768** | **-0.025** | **0.004** | **-0.011** | **49.803** | **1.5542E-12** |
|  | **rs111622870** | **-0.028** | **0.004** | **-0.010** | **41.076** | **1.86E-10** |
|  | **rs190823861** | **-0.035** | **0.005** | **-0.011** | **58.778** | **2.0888E-14** |
|  | **rs148617731** | **0.041** | **0.004** | **0.014** | **90.914** | **5.9156E-22** |
|  | **rs11727162** | **-0.017** | **0.002** | **-0.013** | **80.055** | **2.1548E-19** |
|  | **rs2035901** | **0.024** | **0.002** | **0.019** | **159.557** | **9.4319E-37** |
|  | **rs7679276** | **-0.033** | **0.005** | **-0.010** | **47.266** | **5.9265E-12** |
|  | **rs395980** | **-0.018** | **0.002** | **-0.013** | **76.771** | **1.0209E-17** |
|  | **rs2578565** | **-0.014** | **0.002** | **-0.011** | **49.703** | **1.374E-12** |
|  | **rs12655296** | **-0.011** | **0.002** | **-0.008** | **30.250** | **1.62E-08** |
|  | **rs1177765** | **-0.023** | **0.002** | **-0.018** | **149.097** | **1.3219E-34** |
|  | **rs11959466** | **0.038** | **0.004** | **0.013** | **81.859** | **2.2408E-19** |
|  | **rs62370472** | **-0.025** | **0.002** | **-0.016** | **121.000** | **1.4471E-27** |
|  | **rs10471339** | **-0.011** | **0.002** | **-0.009** | **33.518** | **1.448E-08** |
|  | **rs36048468** | **0.025** | **0.002** | **0.016** | **121.958** | **9.339E-28** |
|  | **rs7735891** | **0.026** | **0.002** | **0.020** | **185.820** | **1.14E-42** |
|  | **rs55758152** | **0.015** | **0.002** | **0.011** | **52.563** | **1.0529E-12** |
|  | **rs57059662** | **0.012** | **0.002** | **0.009** | **34.810** | **5.6311E-09** |
|  | **rs34313173** | **-0.028** | **0.002** | **-0.022** | **223.424** | **3.1688E-50** |
|  | **rs10461725** | **0.013** | **0.002** | **0.010** | **44.890** | **1.9829E-11** |
|  | **rs4865956** | **-0.026** | **0.002** | **-0.018** | **150.939** | **3.8477E-36** |
|  | **rs12517711** | **-0.015** | **0.002** | **-0.012** | **59.859** | **2.7887E-14** |
|  | **rs12188208** | **-0.020** | **0.002** | **-0.013** | **78.564** | **1.4009E-18** |
|  | **rs115912456** | **0.058** | **0.005** | **0.018** | **150.715** | **3.6923E-34** |
|  | **rs861674** | **0.013** | **0.002** | **0.010** | **45.385** | **1.3649E-11** |
|  | **rs10068640** | **0.011** | **0.002** | **0.008** | **31.360** | **1.282E-08** |
|  | **rs2545339** | **0.012** | **0.002** | **0.009** | **33.063** | **3.48E-09** |
|  | **rs144622623** | **0.015** | **0.002** | **0.010** | **43.440** | **4.6655E-11** |
|  | **rs31196** | **-0.011** | **0.002** | **-0.008** | **31.715** | **2.071E-08** |
|  | **rs111365325** | **-0.027** | **0.002** | **-0.018** | **151.738** | **1.1241E-33** |
|  | **rs6874142** | **0.029** | **0.003** | **0.014** | **86.310** | **5.1452E-20** |
|  | **rs10075249** | **0.014** | **0.002** | **0.011** | **56.645** | **4.5583E-14** |
|  | **rs10036789** | **0.016** | **0.002** | **0.013** | **73.598** | **1.1649E-17** |
|  | **rs33986149** | **-0.015** | **0.002** | **-0.012** | **65.695** | **2.23E-15** |
|  | **rs261223** | **0.018** | **0.002** | **0.014** | **84.834** | **2.2999E-19** |
|  | **rs12519407** | **0.018** | **0.002** | **0.012** | **67.688** | **3.3752E-17** |
|  | **rs249677** | **-0.011** | **0.002** | **-0.008** | **29.703** | **2.403E-08** |
|  | **rs13170063** | **-0.015** | **0.002** | **-0.012** | **64.000** | **4.1077E-15** |
|  | **rs447352** | **-0.018** | **0.003** | **-0.009** | **38.955** | **6.6229E-10** |
|  | **rs7731023** | **0.017** | **0.002** | **0.013** | **76.332** | **3.4818E-18** |
|  | **rs7448554** | **-0.013** | **0.002** | **-0.010** | **43.560** | **1.773E-11** |
|  | **rs3822742** | **0.016** | **0.002** | **0.012** | **65.610** | **1.0271E-16** |
|  | **rs4282339** | **-0.031** | **0.002** | **-0.020** | **182.837** | **6.1645E-41** |
|  | **rs244711** | **0.028** | **0.002** | **0.019** | **160.829** | **1.5438E-37** |
|  | **rs40270** | **0.015** | **0.002** | **0.010** | **47.110** | **1.898E-11** |
|  | **rs34287** | **0.019** | **0.002** | **0.014** | **87.423** | **1.1719E-20** |
|  | **rs331917** | **-0.013** | **0.002** | **-0.010** | **44.679** | **3.5416E-11** |
|  | **rs6860245** | **0.059** | **0.002** | **0.040** | **716.779** | **9.661E-160** |
|  | **rs4976262** | **-0.025** | **0.002** | **-0.018** | **150.063** | **4.3722E-33** |
|  | **rs258794** | **0.015** | **0.002** | **0.010** | **49.000** | **6.2345E-12** |
|  | **rs7701233** | **-0.018** | **0.002** | **-0.014** | **88.756** | **5.1204E-21** |
|  | **rs3792819** | **0.021** | **0.003** | **0.009** | **38.149** | **4.422E-10** |
|  | **rs11243202** | **0.030** | **0.002** | **0.024** | **252.643** | **2.8327E-57** |
|  | **rs13209685** | **0.028** | **0.003** | **0.016** | **113.504** | **7.4903E-27** |
|  | **rs2142644** | **-0.018** | **0.002** | **-0.013** | **81.903** | **3.3729E-19** |
|  | **rs78000963** | **0.017** | **0.003** | **0.008** | **29.720** | **4.387E-08** |
|  | **rs370927791** | **-0.021** | **0.002** | **-0.015** | **108.160** | **4.9923E-25** |
|  | **rs2268718** | **0.014** | **0.002** | **0.010** | **45.082** | **3.2359E-11** |
|  | **rs6931421** | **-0.028** | **0.002** | **-0.021** | **194.603** | **2.3052E-43** |
|  | **rs9375188** | **0.014** | **0.002** | **0.011** | **51.235** | **6.8014E-13** |
|  | **rs9391254** | **0.017** | **0.002** | **0.012** | **68.890** | **2.1018E-16** |
|  | **rs113898003** | **-0.036** | **0.002** | **-0.026** | **293.878** | **1.1921E-63** |
|  | **rs1933081** | **0.027** | **0.003** | **0.012** | **61.669** | **5.3284E-15** |
|  | **rs141641494** | **-0.012** | **0.002** | **-0.009** | **33.063** | **8.39E-09** |
|  | **rs9385002** | **-0.015** | **0.002** | **-0.010** | **44.647** | **3.2629E-11** |
|  | **rs7768382** | **-0.020** | **0.002** | **-0.016** | **111.914** | **1.5711E-26** |
|  | **rs372987459** | **0.015** | **0.002** | **0.011** | **54.023** | **6.8517E-14** |
|  | **rs2788213** | **0.012** | **0.002** | **0.009** | **34.306** | **3.796E-09** |
|  | **rs2569888** | **0.013** | **0.002** | **0.009** | **36.548** | **2.293E-09** |
|  | **rs876122** | **0.016** | **0.003** | **0.008** | **31.206** | **2.186E-08** |
|  | **rs41271299** | **0.062** | **0.004** | **0.021** | **205.222** | **3.1923E-47** |
|  | **rs188617336** | **0.014** | **0.002** | **0.010** | **43.184** | **6.6558E-11** |
|  | **rs4380799** | **-0.026** | **0.002** | **-0.018** | **147.449** | **6.4506E-33** |
|  | **rs1319012** | **-0.052** | **0.004** | **-0.021** | **197.516** | **3.2953E-45** |
|  | **rs1324538** | **0.024** | **0.002** | **0.019** | **155.593** | **1.733E-34** |
|  | **rs655113** | **0.019** | **0.002** | **0.013** | **80.145** | **7.4182E-20** |
|  | **rs9344126** | **-0.019** | **0.002** | **-0.015** | **94.806** | **2.206E-22** |
|  | **rs293517** | **-0.013** | **0.002** | **-0.009** | **38.322** | **2.804E-10** |
|  | **rs7768973** | **-0.024** | **0.002** | **-0.019** | **159.557** | **5.4375E-36** |
|  | **rs78051210** | **0.026** | **0.004** | **0.011** | **53.371** | **1.6349E-13** |
|  | **rs10807137** | **-0.046** | **0.003** | **-0.027** | **331.240** | **5.6781E-75** |
|  | **rs9343327** | **0.014** | **0.002** | **0.011** | **54.294** | **1.0909E-13** |
|  | **rs35166681** | **0.021** | **0.003** | **0.012** | **67.746** | **1.5499E-16** |
|  | **rs2754255** | **-0.015** | **0.002** | **-0.010** | **44.251** | **1.0469E-11** |
|  | **rs9388490** | **0.046** | **0.002** | **0.036** | **591.258** | **1.33E-130** |
|  | **rs6902109** | **-0.017** | **0.002** | **-0.013** | **77.255** | **1.034E-18** |
|  | **rs599004** | **-0.016** | **0.002** | **-0.011** | **55.893** | **6.5283E-14** |
|  | **rs2748501** | **-0.020** | **0.002** | **-0.015** | **105.332** | **1.311E-24** |
|  | **rs718603** | **0.013** | **0.002** | **0.009** | **38.914** | **6.679E-10** |
|  | **rs9266244** | **-0.043** | **0.002** | **-0.030** | **413.444** | **1.21E-94** |
|  | **rs72894003** | **-0.042** | **0.004** | **-0.017** | **123.912** | **1.898E-28** |
|  | **rs2764264** | **0.020** | **0.002** | **0.014** | **93.444** | **5.0734E-23** |
|  | **rs6570509** | **-0.024** | **0.002** | **-0.017** | **135.002** | **1.2659E-31** |
|  | **rs543650** | **0.025** | **0.002** | **0.019** | **156.250** | **1.4931E-37** |
|  | **rs3828729** | **-0.016** | **0.002** | **-0.012** | **64.000** | **4.6687E-15** |
|  | **rs2105333** | **-0.019** | **0.002** | **-0.014** | **90.250** | **1.701E-21** |
|  | **rs2763263** | **-0.017** | **0.002** | **-0.012** | **59.711** | **1.3709E-14** |
|  | **rs798548** | **-0.036** | **0.002** | **-0.025** | **292.247** | **2.8589E-68** |
|  | **rs12533452** | **0.024** | **0.003** | **0.014** | **83.090** | **1.314E-19** |
|  | **rs34776209** | **-0.032** | **0.002** | **-0.021** | **207.622** | **1.7779E-47** |
|  | **rs12536902** | **0.048** | **0.008** | **0.009** | **34.970** | **3.656E-09** |
|  | **rs1880318** | **0.015** | **0.002** | **0.009** | **37.516** | **6.859E-10** |
|  | **rs73696333** | **0.019** | **0.002** | **0.012** | **63.335** | **3.1967E-15** |
|  | **rs35732917** | **0.020** | **0.002** | **0.014** | **94.367** | **2.0802E-22** |
|  | **rs139163241** | **-0.016** | **0.003** | **-0.009** | **36.894** | **1.786E-09** |
|  | **rs62466110** | **-0.037** | **0.004** | **-0.013** | **81.880** | **5.7412E-20** |
|  | **rs143355941** | **0.019** | **0.002** | **0.015** | **98.950** | **4.6527E-23** |
|  | **rs987666** | **0.019** | **0.003** | **0.010** | **40.696** | **2.327E-10** |
|  | **rs62621812** | **0.074** | **0.007** | **0.016** | **115.952** | **3.163E-27** |
|  | **rs757834** | **0.026** | **0.002** | **0.016** | **113.778** | **1.248E-25** |
|  | **rs28529426** | **-0.017** | **0.003** | **-0.010** | **41.751** | **5.8237E-11** |
|  | **rs10242866** | **0.016** | **0.002** | **0.012** | **68.280** | **3.6627E-16** |
|  | **rs2529090** | **0.014** | **0.003** | **0.008** | **29.594** | **3.386E-08** |
|  | **rs2237485** | **0.019** | **0.002** | **0.012** | **68.962** | **3.7342E-17** |
|  | **rs10627558** | **-0.018** | **0.002** | **-0.012** | **67.688** | **6.9104E-16** |
|  | **rs12672217** | **0.014** | **0.002** | **0.010** | **48.303** | **1.3341E-12** |
|  | **rs177591** | **-0.019** | **0.003** | **-0.011** | **50.043** | **1.5129E-12** |
|  | **rs60408354** | **0.026** | **0.004** | **0.011** | **51.760** | **1.1521E-12** |
|  | **rs42039** | **0.048** | **0.002** | **0.033** | **478.019** | **3.532E-106** |
|  | **rs56363908** | **-0.038** | **0.005** | **-0.012** | **66.059** | **3.8779E-16** |
|  | **rs2140619** | **0.011** | **0.002** | **0.009** | **35.371** | **5.178E-09** |
|  | **rs3778858** | **0.011** | **0.002** | **0.008** | **29.160** | **4.262E-08** |
|  | **rs822530** | **0.026** | **0.002** | **0.016** | **112.891** | **2.361E-27** |
|  | **rs12702693** | **0.017** | **0.002** | **0.014** | **82.906** | **6.5615E-20** |
|  | **rs680882** | **0.013** | **0.002** | **0.009** | **36.548** | **1.984E-09** |
|  | **rs723149** | **-0.028** | **0.002** | **-0.022** | **211.014** | **1.4319E-47** |
|  | **rs1202186** | **-0.012** | **0.002** | **-0.009** | **36.000** | **1.653E-09** |
|  | **rs9640283** | **-0.012** | **0.002** | **-0.009** | **39.227** | **4.534E-10** |
|  | **rs6977416** | **0.046** | **0.002** | **0.034** | **522.123** | **1.429E-113** |
|  | **rs10225945** | **-0.015** | **0.003** | **-0.008** | **31.533** | **3.276E-08** |
|  | **rs12700901** | **-0.018** | **0.002** | **-0.014** | **93.784** | **1.6669E-21** |
|  | **rs6593210** | **0.015** | **0.002** | **0.009** | **37.007** | **4.882E-10** |
|  | **rs11562101** | **0.012** | **0.002** | **0.009** | **33.063** | **9.386E-09** |
|  | **rs2188805** | **0.011** | **0.002** | **0.008** | **32.490** | **2.003E-08** |
|  | **rs76364830** | **-0.047** | **0.004** | **-0.018** | **145.852** | **2.7027E-33** |
|  | **rs7826059** | **0.011** | **0.002** | **0.008** | **32.490** | **7.614E-09** |
|  | **rs1063582** | **-0.019** | **0.002** | **-0.013** | **70.713** | **1.1241E-16** |
|  | **rs117818446** | **0.042** | **0.007** | **0.009** | **38.696** | **5.289E-10** |
|  | **rs7014590** | **-0.023** | **0.002** | **-0.015** | **107.405** | **4.4771E-26** |
|  | **rs112537273** | **-0.021** | **0.002** | **-0.014** | **92.860** | **3.3443E-21** |
|  | **rs4602848** | **0.016** | **0.002** | **0.012** | **64.000** | **3.1449E-15** |
|  | **rs35086476** | **0.011** | **0.002** | **0.009** | **32.911** | **8.4269E-09** |
|  | **rs2142331** | **-0.017** | **0.002** | **-0.013** | **75.416** | **1.3778E-17** |
|  | **rs6470771** | **-0.027** | **0.003** | **-0.016** | **114.918** | **1.5449E-26** |
|  | **rs12334478** | **-0.016** | **0.002** | **-0.013** | **71.803** | **2.2662E-17** |
|  | **rs10112506** | **-0.012** | **0.002** | **-0.009** | **39.889** | **5.7571E-10** |
|  | **rs7816345** | **0.026** | **0.003** | **0.015** | **104.040** | **6.2244E-24** |
|  | **rs2923411** | **0.013** | **0.002** | **0.010** | **44.679** | **4.7698E-11** |
|  | **rs72656010** | **-0.067** | **0.003** | **-0.036** | **569.163** | **7.311E-126** |
|  | **rs7828086** | **0.014** | **0.002** | **0.009** | **37.655** | **1.111E-09** |
|  | **rs62501195** | **-0.020** | **0.003** | **-0.012** | **62.726** | **8.1134E-15** |
|  | **rs62515437** | **0.037** | **0.002** | **0.024** | **257.393** | **8.7882E-60** |
|  | **rs2925155** | **-0.015** | **0.002** | **-0.010** | **46.488** | **5.4664E-12** |
|  | **rs11778491** | **-0.025** | **0.002** | **-0.017** | **126.052** | **8.3869E-30** |
|  | **rs1340022** | **0.012** | **0.002** | **0.009** | **38.571** | **4.474E-10** |
|  | **rs72721979** | **-0.023** | **0.003** | **-0.013** | **71.936** | **2.1989E-17** |
|  | **rs7007389** | **-0.013** | **0.002** | **-0.010** | **44.890** | **2.9437E-11** |
|  | **rs115105539** | **0.023** | **0.003** | **0.014** | **85.378** | **7.8253E-20** |
|  | **rs4077103** | **-0.014** | **0.003** | **-0.008** | **30.250** | **4.175E-08** |
|  | **rs61729527** | **-0.035** | **0.004** | **-0.012** | **64.746** | **4.8585E-16** |
|  | **rs4735761** | **0.033** | **0.002** | **0.023** | **248.438** | **3.6619E-56** |
|  | **rs10283100** | **0.058** | **0.004** | **0.021** | **196.684** | **4.1068E-44** |
|  | **rs4870941** | **-0.030** | **0.002** | **-0.019** | **166.747** | **1.0919E-39** |
|  | **rs12541381** | **-0.032** | **0.002** | **-0.022** | **210.250** | **2.8093E-49** |
|  | **rs10107388** | **-0.016** | **0.002** | **-0.012** | **63.203** | **6.9534E-16** |
|  | **rs10815274** | **0.012** | **0.002** | **0.010** | **42.593** | **6.4565E-11** |
|  | **rs7858712** | **0.035** | **0.003** | **0.015** | **104.160** | **1.043E-24** |
|  | **rs34522021** | **0.013** | **0.002** | **0.010** | **43.978** | **3.376E-11** |
|  | **rs75508358** | **0.027** | **0.005** | **0.009** | **34.941** | **4.29E-09** |
|  | **rs12347137** | **-0.046** | **0.002** | **-0.029** | **367.361** | **9.7972E-85** |
|  | **rs10123619** | **-0.017** | **0.003** | **-0.010** | **43.256** | **4.0504E-11** |
|  | **rs10975935** | **-0.012** | **0.002** | **-0.008** | **30.250** | **4.15E-08** |
|  | **rs1056747** | **-0.016** | **0.002** | **-0.012** | **66.551** | **8.0482E-16** |
|  | **rs143554698** | **-0.026** | **0.003** | **-0.014** | **90.602** | **3.3939E-21** |
|  | **rs10982888** | **-0.033** | **0.003** | **-0.016** | **119.538** | **3.9664E-28** |
|  | **rs73384223** | **-0.021** | **0.002** | **-0.013** | **72.960** | **1.2659E-17** |
|  | **rs10962212** | **0.014** | **0.002** | **0.011** | **56.645** | **7.4748E-14** |
|  | **rs7863102** | **-0.011** | **0.002** | **-0.009** | **33.518** | **9.974E-09** |
|  | **rs3901421** | **0.022** | **0.002** | **0.017** | **128.047** | **7.5475E-30** |
|  | **rs2236406** | **0.039** | **0.002** | **0.029** | **388.090** | **1.2581E-87** |
|  | **rs373966865** | **0.039** | **0.003** | **0.023** | **242.114** | **1.8442E-53** |
|  | **rs3205136** | **-0.018** | **0.003** | **-0.008** | **31.089** | **1.85E-08** |
|  | **rs10858246** | **-0.019** | **0.002** | **-0.014** | **88.360** | **2.3491E-20** |
|  | **rs1330826** | **0.016** | **0.002** | **0.010** | **49.611** | **1.039E-12** |
|  | **rs7020491** | **-0.018** | **0.002** | **-0.014** | **87.767** | **1.219E-20** |
|  | **rs74458759** | **0.017** | **0.002** | **0.012** | **60.415** | **3.1203E-15** |
|  | **rs12340775** | **-0.029** | **0.004** | **-0.010** | **44.548** | **1.7318E-11** |
|  | **rs12351226** | **0.022** | **0.003** | **0.013** | **76.038** | **9.1348E-18** |
|  | **rs1341215** | **0.023** | **0.003** | **0.013** | **71.936** | **6.3183E-17** |
|  | **rs12344515** | **-0.016** | **0.002** | **-0.011** | **54.895** | **2.2772E-13** |
|  | **rs80280630** | **-0.017** | **0.003** | **-0.008** | **31.360** | **2.279E-08** |
|  | **rs112367251** | **0.018** | **0.002** | **0.013** | **72.655** | **1.2909E-17** |
|  | **rs10793931** | **-0.013** | **0.002** | **-0.010** | **43.560** | **3.2322E-11** |
|  | **rs7082659** | **0.016** | **0.003** | **0.008** | **31.041** | **2.273E-08** |
|  | **rs10829226** | **-0.011** | **0.002** | **-0.008** | **31.360** | **1.33E-08** |
|  | **rs10776560** | **-0.016** | **0.002** | **-0.012** | **68.280** | **7.8795E-17** |
|  | **rs68049170** | **-0.026** | **0.002** | **-0.018** | **152.111** | **2.6903E-34** |
|  | **rs2274351** | **0.017** | **0.002** | **0.013** | **80.055** | **3.0662E-19** |
|  | **rs72841270** | **0.029** | **0.003** | **0.016** | **110.250** | **2.2522E-26** |
|  | **rs11421589** | **0.012** | **0.002** | **0.009** | **39.227** | **5.9889E-10** |
|  | **rs11424084** | **0.023** | **0.003** | **0.013** | **71.309** | **7.241E-17** |
|  | **rs1556659** | **0.016** | **0.002** | **0.012** | **66.423** | **7.1895E-17** |
|  | **rs35288270** | **-0.033** | **0.003** | **-0.017** | **137.224** | **3.4348E-32** |
|  | **rs4748008** | **-0.013** | **0.002** | **-0.010** | **43.283** | **8.9495E-11** |
|  | **rs332116** | **-0.021** | **0.002** | **-0.015** | **96.227** | **2.886E-22** |
|  | **rs10822117** | **-0.018** | **0.002** | **-0.012** | **64.000** | **4.2403E-15** |
|  | **rs67527161** | **-0.018** | **0.002** | **-0.012** | **62.616** | **5.7903E-15** |
|  | **rs10128333** | **-0.015** | **0.003** | **-0.009** | **34.106** | **9.5131E-09** |
|  | **rs7095472** | **0.027** | **0.002** | **0.021** | **197.476** | **7.656E-45** |
|  | **rs117335233** | **-0.024** | **0.004** | **-0.008** | **31.574** | **2.561E-08** |
|  | **rs12773500** | **0.017** | **0.003** | **0.009** | **37.297** | **5.0541E-10** |
|  | **rs11187838** | **0.039** | **0.002** | **0.031** | **430.017** | **1.1601E-94** |
|  | **rs2181834** | **0.025** | **0.002** | **0.020** | **178.715** | **7.6949E-41** |
|  | **rs496783** | **-0.012** | **0.002** | **-0.010** | **42.593** | **8.1264E-11** |
|  | **rs11198591** | **0.015** | **0.002** | **0.011** | **54.760** | **4.908E-14** |
|  | **rs2362487** | **0.015** | **0.002** | **0.010** | **49.000** | **3.5703E-12** |
|  | **rs947099** | **0.012** | **0.002** | **0.009** | **34.223** | **2.707E-09** |
|  | **rs71463518** | **-0.013** | **0.002** | **-0.009** | **39.510** | **2.921E-10** |
|  | **rs10824307** | **-0.019** | **0.002** | **-0.014** | **94.090** | **1.789E-22** |
|  | **rs664317** | **-0.018** | **0.003** | **-0.010** | **46.345** | **4.4957E-12** |
|  | **rs2648725** | **0.017** | **0.002** | **0.011** | **51.465** | **8.2035E-13** |
|  | **rs7893378** | **0.018** | **0.003** | **0.008** | **31.868** | **2.36E-08** |
|  | **rs291979** | **0.024** | **0.002** | **0.016** | **110.707** | **6.7562E-27** |
|  | **rs11009928** | **-0.015** | **0.002** | **-0.010** | **45.256** | **1.038E-11** |
|  | **rs5786398** | **-0.018** | **0.002** | **-0.013** | **71.041** | **1.5389E-17** |
|  | **rs4752689** | **0.021** | **0.002** | **0.016** | **116.413** | **1.363E-26** |
|  | **rs11014285** | **0.034** | **0.003** | **0.020** | **173.024** | **2.888E-40** |
|  | **rs2490302** | **0.022** | **0.003** | **0.010** | **42.250** | **6.2374E-11** |
|  | **rs11191208** | **0.015** | **0.002** | **0.009** | **37.516** | **3.689E-10** |
|  | **rs10749157** | **0.011** | **0.002** | **0.008** | **31.923** | **1.239E-08** |
|  | **rs2283200** | **-0.028** | **0.004** | **-0.010** | **44.762** | **1.4812E-11** |
|  | **rs73413540** | **-0.012** | **0.002** | **-0.008** | **29.066** | **4.421E-08** |
|  | **rs985136** | **0.014** | **0.002** | **0.010** | **47.610** | **2.9621E-12** |
|  | **rs4752829** | **0.026** | **0.002** | **0.019** | **155.655** | **5.902E-36** |
|  | **rs10796828** | **0.015** | **0.002** | **0.011** | **59.290** | **5.7743E-15** |
|  | **rs7902** | **0.015** | **0.002** | **0.012** | **61.499** | **5.2192E-15** |
|  | **rs11221657** | **0.018** | **0.003** | **0.010** | **40.869** | **1.205E-10** |
|  | **rs112873218** | **0.022** | **0.003** | **0.010** | **48.549** | **4.0216E-12** |
|  | **rs10832963** | **-0.020** | **0.002** | **-0.014** | **85.143** | **9.2257E-21** |
|  | **rs704660** | **0.015** | **0.002** | **0.012** | **64.845** | **2.2772E-15** |
|  | **rs7107356** | **0.013** | **0.002** | **0.010** | **49.000** | **1.8599E-12** |
|  | **rs11233117** | **-0.018** | **0.002** | **-0.014** | **85.806** | **1.7418E-20** |
|  | **rs73006226** | **-0.018** | **0.003** | **-0.009** | **39.386** | **2.23E-10** |
|  | **rs545104** | **0.013** | **0.002** | **0.009** | **40.323** | **8.0835E-11** |
|  | **rs56207600** | **0.019** | **0.003** | **0.010** | **40.960** | **2.501E-10** |
|  | **rs61878760** | **0.019** | **0.003** | **0.008** | **31.228** | **3.736E-08** |
|  | **rs7952436** | **-0.045** | **0.003** | **-0.020** | **177.516** | **1.6181E-39** |
|  | **rs4244809** | **-0.026** | **0.002** | **-0.017** | **129.762** | **5.5539E-29** |
|  | **rs1584011** | **0.016** | **0.002** | **0.012** | **63.203** | **9.7769E-16** |
|  | **rs7941305** | **-0.013** | **0.002** | **-0.009** | **37.735** | **6.012E-10** |
|  | **rs11605297** | **0.015** | **0.002** | **0.010** | **44.041** | **8.0334E-11** |
|  | **rs7129320** | **-0.039** | **0.003** | **-0.023** | **242.114** | **7.2895E-53** |
|  | **rs34345560** | **0.022** | **0.002** | **0.014** | **83.266** | **7.0974E-20** |
|  | **rs604723** | **-0.017** | **0.002** | **-0.012** | **62.485** | **8.1602E-15** |
|  | **rs11042717** | **-0.029** | **0.002** | **-0.023** | **232.964** | **4.043E-53** |
|  | **rs10657263** | **-0.013** | **0.002** | **-0.010** | **46.814** | **7.98E-12** |
|  | **rs4938359** | **-0.016** | **0.002** | **-0.010** | **42.250** | **3.2397E-11** |
|  | **rs11217863** | **-0.027** | **0.003** | **-0.013** | **79.804** | **1.0629E-19** |
|  | **rs772222** | **0.012** | **0.002** | **0.009** | **33.200** | **1.552E-08** |
|  | **rs1168768** | **0.033** | **0.006** | **0.008** | **30.618** | **3.609E-08** |
|  | **rs2089111** | **-0.017** | **0.002** | **-0.012** | **61.124** | **1.7258E-15** |
|  | **rs4622329** | **0.015** | **0.002** | **0.011** | **55.503** | **8.6199E-14** |
|  | **rs3764002** | **0.028** | **0.002** | **0.020** | **177.778** | **4.471E-39** |
|  | **rs12423821** | **0.016** | **0.003** | **0.009** | **35.557** | **1.172E-09** |
|  | **rs7137546** | **0.014** | **0.002** | **0.011** | **55.856** | **9.7073E-14** |
|  | **rs67551338** | **0.058** | **0.004** | **0.021** | **207.360** | **1.044E-47** |
|  | **rs10845408** | **0.026** | **0.002** | **0.019** | **162.563** | **3.2539E-38** |
|  | **rs17478946** | **-0.019** | **0.002** | **-0.014** | **83.592** | **9.9839E-21** |
|  | **rs12230946** | **0.027** | **0.003** | **0.012** | **67.439** | **1.4501E-16** |
|  | **rs9669278** | **-0.050** | **0.002** | **-0.039** | **681.485** | **5.248E-151** |
|  | **rs2229840** | **0.034** | **0.003** | **0.020** | **172.013** | **3.0151E-40** |
|  | **rs7485647** | **-0.026** | **0.003** | **-0.015** | **100.771** | **1.045E-23** |
|  | **rs35756741** | **-0.038** | **0.003** | **-0.017** | **131.207** | **5.801E-31** |
|  | **rs6582398** | **0.014** | **0.002** | **0.010** | **49.000** | **1.135E-12** |
|  | **rs10748128** | **0.026** | **0.002** | **0.019** | **162.563** | **6.7717E-38** |
|  | **rs11178643** | **0.011** | **0.002** | **0.008** | **29.703** | **4.44E-08** |
|  | **rs310796** | **0.014** | **0.002** | **0.011** | **50.410** | **2.534E-12** |
|  | **rs9634212** | **0.047** | **0.002** | **0.031** | **419.359** | **8.5901E-95** |
|  | **rs7971536** | **-0.019** | **0.002** | **-0.015** | **104.255** | **1.0649E-24** |
|  | **rs2454390** | **-0.018** | **0.003** | **-0.010** | **45.822** | **1.7398E-11** |
|  | **rs11612462** | **0.015** | **0.003** | **0.009** | **36.000** | **2.547E-09** |
|  | **rs34338597** | **-0.011** | **0.002** | **-0.009** | **34.748** | **7.8801E-09** |
|  | **rs3184504** | **0.018** | **0.002** | **0.014** | **92.767** | **2.7133E-22** |
|  | **rs610694** | **0.014** | **0.002** | **0.011** | **51.235** | **4.2875E-13** |
|  | **rs76895963** | **0.164** | **0.007** | **0.033** | **504.095** | **8.222E-112** |
|  | **rs11175919** | **0.035** | **0.006** | **0.009** | **34.990** | **3.159E-09** |
|  | **rs11068230** | **0.024** | **0.003** | **0.013** | **72.250** | **9.6739E-18** |
|  | **rs2101017** | **-0.022** | **0.003** | **-0.012** | **63.430** | **1.4441E-15** |
|  | **rs11060942** | **-0.035** | **0.005** | **-0.010** | **46.345** | **7.4903E-12** |
|  | **rs28592876** | **0.030** | **0.002** | **0.019** | **170.132** | **9.0866E-38** |
|  | **rs3782811** | **-0.017** | **0.002** | **-0.011** | **56.250** | **3.9591E-14** |
|  | **rs61919240** | **0.014** | **0.002** | **0.010** | **46.923** | **9.7701E-12** |
|  | **rs1444628** | **0.024** | **0.002** | **0.018** | **144.000** | **6.8454E-32** |
|  | **rs11049704** | **-0.018** | **0.002** | **-0.013** | **75.939** | **1.1031E-18** |
|  | **rs12831751** | **0.017** | **0.002** | **0.012** | **67.084** | **1.574E-16** |
|  | **rs12099669** | **0.033** | **0.002** | **0.025** | **273.903** | **1.3919E-58** |
|  | **rs2071450** | **-0.017** | **0.002** | **-0.013** | **75.690** | **8.8471E-19** |
|  | **rs3782232** | **-0.034** | **0.004** | **-0.014** | **83.945** | **2.406E-20** |
|  | **rs7301341** | **-0.026** | **0.002** | **-0.019** | **162.563** | **9.2747E-37** |
|  | **rs7321635** | **-0.013** | **0.002** | **-0.010** | **43.560** | **2.5781E-11** |
|  | **rs3116602** | **-0.061** | **0.002** | **-0.040** | **708.023** | **9.528E-155** |
|  | **rs3818416** | **0.028** | **0.002** | **0.019** | **160.829** | **2.0082E-35** |
|  | **rs61944841** | **0.025** | **0.002** | **0.019** | **160.023** | **3.5408E-37** |
|  | **rs77013652** | **0.049** | **0.008** | **0.009** | **36.595** | **1.502E-09** |
|  | **rs7328187** | **0.012** | **0.002** | **0.009** | **37.274** | **1.185E-09** |
|  | **rs7320878** | **-0.015** | **0.002** | **-0.012** | **62.327** | **1.344E-14** |
|  | **rs144109601** | **-0.028** | **0.005** | **-0.009** | **33.543** | **5.15E-09** |
|  | **rs9590328** | **0.015** | **0.003** | **0.008** | **32.111** | **2.02E-08** |
|  | **rs78525785** | **-0.017** | **0.002** | **-0.013** | **71.403** | **9.813E-18** |
|  | **rs9594714** | **0.014** | **0.002** | **0.010** | **47.020** | **2.6479E-12** |
|  | **rs9568031** | **-0.012** | **0.002** | **-0.008** | **29.989** | **3.339E-08** |
|  | **rs2812208** | **0.116** | **0.007** | **0.026** | **306.781** | **5.5068E-68** |
|  | **rs8000973** | **0.013** | **0.002** | **0.011** | **49.740** | **2.0502E-12** |
|  | **rs9525326** | **-0.018** | **0.002** | **-0.011** | **58.778** | **3.6241E-14** |
|  | **rs532499** | **-0.013** | **0.002** | **-0.009** | **33.324** | **4.9E-09** |
|  | **rs9517483** | **-0.018** | **0.002** | **-0.013** | **74.288** | **2.26E-18** |
|  | **rs2296316** | **-0.019** | **0.002** | **-0.015** | **102.116** | **1.5889E-23** |
|  | **rs113827862** | **-0.024** | **0.004** | **-0.009** | **34.516** | **4.667E-09** |
|  | **rs4900578** | **-0.018** | **0.002** | **-0.013** | **78.323** | **1.7559E-19** |
|  | **rs56112295** | **0.015** | **0.002** | **0.010** | **41.174** | **1.118E-10** |
|  | **rs8019890** | **0.025** | **0.002** | **0.020** | **173.130** | **1.9588E-38** |
|  | **rs8017006** | **0.012** | **0.002** | **0.009** | **37.210** | **2.22E-09** |
|  | **rs10483727** | **-0.037** | **0.002** | **-0.029** | **375.136** | **6.7298E-80** |
|  | **rs8020095** | **-0.015** | **0.003** | **-0.008** | **28.841** | **4.534E-08** |
|  | **rs35230100** | **-0.026** | **0.002** | **-0.020** | **171.610** | **4.4157E-40** |
|  | **rs117068593** | **0.040** | **0.002** | **0.025** | **281.960** | **8.8267E-62** |
|  | **rs1190540** | **0.013** | **0.002** | **0.009** | **35.431** | **1.619E-09** |
|  | **rs17197114** | **0.018** | **0.003** | **0.011** | **50.126** | **1.5438E-12** |
|  | **rs45528934** | **0.026** | **0.003** | **0.015** | **101.544** | **1.9652E-24** |
|  | **rs28529055** | **-0.015** | **0.002** | **-0.012** | **59.859** | **1.8789E-14** |
|  | **rs36226649** | **0.049** | **0.004** | **0.019** | **162.898** | **3.0528E-37** |
|  | **rs28678024** | **-0.012** | **0.002** | **-0.008** | **32.111** | **1.899E-08** |
|  | **rs8904** | **-0.016** | **0.002** | **-0.012** | **61.623** | **1.523E-15** |
|  | **rs10637890** | **-0.012** | **0.002** | **-0.009** | **37.823** | **3.956E-10** |
|  | **rs2070598** | **0.020** | **0.002** | **0.016** | **115.280** | **6.3562E-27** |
|  | **rs909220** | **-0.015** | **0.002** | **-0.012** | **62.327** | **3.2449E-15** |
|  | **rs79066296** | **-0.017** | **0.002** | **-0.011** | **59.010** | **6.1066E-14** |
|  | **rs12882130** | **-0.020** | **0.002** | **-0.015** | **102.010** | **1.8772E-24** |
|  | **rs8018486** | **-0.014** | **0.002** | **-0.009** | **33.063** | **1.178E-08** |
|  | **rs7144307** | **-0.012** | **0.002** | **-0.009** | **37.210** | **6.0971E-10** |
|  | **rs13316** | **0.012** | **0.002** | **0.009** | **36.634** | **3.655E-09** |
|  | **rs147233090** | **-0.045** | **0.006** | **-0.011** | **53.458** | **3.9537E-13** |
|  | **rs5812543** | **0.014** | **0.002** | **0.010** | **41.327** | **2.647E-10** |
|  | **rs4383083** | **0.011** | **0.002** | **0.008** | **30.803** | **2.909E-08** |
|  | **rs8042578** | **0.029** | **0.002** | **0.019** | **170.184** | **2.2851E-38** |
|  | **rs990315** | **-0.012** | **0.002** | **-0.009** | **33.063** | **5.07E-09** |
|  | **rs74379684** | **-0.027** | **0.004** | **-0.011** | **57.086** | **4.3914E-14** |
|  | **rs2871865** | **-0.049** | **0.003** | **-0.024** | **270.054** | **3.4041E-62** |
|  | **rs17205463** | **-0.026** | **0.002** | **-0.021** | **191.604** | **4.2092E-43** |
|  | **rs36016415** | **-0.028** | **0.002** | **-0.022** | **209.488** | **3.2218E-47** |
|  | **rs4965298** | **-0.012** | **0.002** | **-0.008** | **32.111** | **1.81E-08** |
|  | **rs11070842** | **-0.015** | **0.003** | **-0.008** | **31.533** | **1.301E-08** |
|  | **rs12907139** | **-0.015** | **0.002** | **-0.012** | **61.499** | **4.8933E-15** |
|  | **rs5742915** | **0.025** | **0.002** | **0.019** | **170.371** | **9.3261E-39** |
|  | **rs140657345** | **-0.020** | **0.002** | **-0.014** | **82.645** | **5.5335E-20** |
|  | **rs373736365** | **0.019** | **0.002** | **0.013** | **73.804** | **4.5342E-18** |
|  | **rs11633371** | **0.022** | **0.002** | **0.017** | **129.241** | **7.4938E-30** |
|  | **rs4932439** | **-0.015** | **0.003** | **-0.009** | **36.482** | **1.433E-09** |
|  | **rs2174008** | **-0.019** | **0.002** | **-0.015** | **102.116** | **5.8925E-24** |
|  | **rs577289** | **-0.013** | **0.002** | **-0.009** | **35.431** | **5.394E-09** |
|  | **rs72726050** | **-0.019** | **0.003** | **-0.008** | **31.889** | **1.841E-08** |
|  | **rs12909863** | **0.019** | **0.002** | **0.013** | **73.804** | **6.0464E-18** |
|  | **rs713467** | **0.015** | **0.002** | **0.011** | **59.047** | **3.0939E-14** |
|  | **rs11629593** | **-0.011** | **0.002** | **-0.008** | **29.703** | **4.422E-08** |
|  | **rs2663126** | **-0.014** | **0.002** | **-0.010** | **43.812** | **1.3561E-11** |
|  | **rs116092985** | **-0.040** | **0.003** | **-0.018** | **147.659** | **1.1679E-34** |
|  | **rs35811052** | **-0.015** | **0.002** | **-0.010** | **45.256** | **8.841E-12** |
|  | **rs72771070** | **0.015** | **0.002** | **0.011** | **51.020** | **1.2969E-12** |
|  | **rs62033029** | **-0.014** | **0.002** | **-0.009** | **37.582** | **1.726E-09** |
|  | **rs72801843** | **0.031** | **0.002** | **0.022** | **222.152** | **8.8267E-52** |
|  | **rs55872725** | **0.022** | **0.002** | **0.017** | **136.521** | **1.4629E-30** |
|  | **rs4985445** | **-0.018** | **0.002** | **-0.014** | **84.834** | **3.2953E-20** |
|  | **rs17818592** | **-0.013** | **0.002** | **-0.010** | **46.097** | **1.2291E-11** |
|  | **rs8054549** | **-0.025** | **0.002** | **-0.020** | **174.518** | **3.3729E-39** |
|  | **rs7185244** | **-0.015** | **0.002** | **-0.010** | **41.406** | **1.396E-10** |
|  | **rs113478686** | **-0.024** | **0.002** | **-0.016** | **112.544** | **3.7636E-27** |
|  | **rs12051245** | **0.030** | **0.002** | **0.020** | **184.713** | **2.5639E-40** |
|  | **rs143076454** | **-0.050** | **0.007** | **-0.011** | **50.817** | **1.0551E-12** |
|  | **rs246177** | **0.021** | **0.002** | **0.016** | **114.490** | **2.0352E-27** |
|  | **rs116008080** | **-0.042** | **0.006** | **-0.010** | **43.393** | **4.057E-11** |
|  | **rs12926103** | **0.027** | **0.004** | **0.011** | **51.235** | **9.5984E-13** |
|  | **rs77364196** | **-0.033** | **0.004** | **-0.011** | **58.897** | **8.3483E-15** |
|  | **rs61528919** | **0.014** | **0.002** | **0.010** | **49.000** | **3.3289E-12** |
|  | **rs35816944** | **-0.109** | **0.012** | **-0.014** | **86.474** | **1.2659E-20** |
|  | **rs78457529** | **-0.090** | **0.009** | **-0.015** | **105.529** | **1.221E-24** |
|  | **rs4788218** | **0.028** | **0.002** | **0.022** | **209.488** | **5.5246E-46** |
|  | **rs2240735** | **0.019** | **0.002** | **0.013** | **73.804** | **3.9857E-18** |
|  | **rs77809369** | **0.024** | **0.004** | **0.009** | **36.929** | **9.8349E-10** |
|  | **rs35268848** | **0.074** | **0.010** | **0.011** | **53.247** | **2.832E-13** |
|  | **rs62070319** | **-0.018** | **0.002** | **-0.013** | **81.000** | **2.541E-20** |
|  | **rs6502935** | **-0.013** | **0.002** | **-0.008** | **32.283** | **6.315E-09** |
|  | **rs113146332** | **0.031** | **0.005** | **0.009** | **40.284** | **2.987E-10** |
|  | **rs9894577** | **-0.031** | **0.002** | **-0.023** | **240.250** | **1.3951E-52** |
|  | **rs2005172** | **0.048** | **0.002** | **0.036** | **576.000** | **2.35E-128** |
|  | **rs28485212** | **-0.019** | **0.003** | **-0.010** | **48.483** | **1.2419E-12** |
|  | **rs9890062** | **0.027** | **0.004** | **0.010** | **46.870** | **1.2001E-11** |
|  | **rs117972846** | **0.034** | **0.006** | **0.009** | **34.541** | **5.4729E-09** |
|  | **rs2289629** | **-0.015** | **0.002** | **-0.011** | **54.760** | **8.0205E-14** |
|  | **rs2019203** | **0.019** | **0.002** | **0.015** | **98.950** | **1.8412E-23** |
|  | **rs9905385** | **-0.034** | **0.002** | **-0.025** | **287.303** | **1.94E-63** |
|  | **rs78766798** | **0.032** | **0.004** | **0.014** | **83.070** | **2.6098E-20** |
|  | **rs78378222** | **0.138** | **0.009** | **0.024** | **251.605** | **4.5144E-56** |
|  | **rs2112617** | **-0.017** | **0.002** | **-0.013** | **77.255** | **1.073E-18** |
|  | **rs2676298** | **-0.027** | **0.003** | **-0.015** | **99.261** | **2.3851E-23** |
|  | **rs7220127** | **-0.011** | **0.002** | **-0.008** | **30.540** | **4.603E-08** |
|  | **rs36000545** | **-0.022** | **0.002** | **-0.016** | **121.000** | **2.558E-29** |
|  | **rs6505216** | **-0.050** | **0.002** | **-0.032** | **468.817** | **1.828E-101** |
|  | **rs57791149** | **-0.017** | **0.002** | **-0.014** | **82.906** | **3.2614E-19** |
|  | **rs2521349** | **0.016** | **0.002** | **0.012** | **66.551** | **2.0142E-15** |
|  | **rs173135** | **-0.034** | **0.003** | **-0.017** | **129.201** | **3.2456E-30** |
|  | **rs12943867** | **0.018** | **0.002** | **0.014** | **84.640** | **7.5683E-20** |
|  | **rs9898189** | **-0.016** | **0.002** | **-0.012** | **60.247** | **1.8802E-15** |
|  | **rs57513571** | **-0.019** | **0.002** | **-0.012** | **63.335** | **7.021E-16** |
|  | **rs11867855** | **-0.013** | **0.002** | **-0.009** | **36.000** | **2.931E-09** |
|  | **rs4640244** | **-0.020** | **0.002** | **-0.016** | **110.803** | **3.8098E-25** |
|  | **rs72829852** | **0.031** | **0.004** | **0.012** | **62.775** | **3.7402E-15** |
|  | **rs2592208** | **-0.012** | **0.002** | **-0.010** | **42.593** | **5.517E-11** |
|  | **rs113232639** | **0.033** | **0.002** | **0.026** | **296.202** | **4.7896E-64** |
|  | **rs8084413** | **-0.013** | **0.002** | **-0.010** | **44.679** | **3.2471E-11** |
|  | **rs2978362** | **0.011** | **0.002** | **0.008** | **31.125** | **2.848E-08** |
|  | **rs71336393** | **0.049** | **0.002** | **0.032** | **453.875** | **9.016E-105** |
|  | **rs74494415** | **-0.042** | **0.005** | **-0.013** | **72.424** | **1.8218E-17** |
|  | **rs2347808** | **-0.013** | **0.002** | **-0.010** | **43.283** | **5.7876E-11** |
|  | **rs33973388** | **0.025** | **0.002** | **0.020** | **171.748** | **1.4461E-38** |
|  | **rs4940874** | **0.015** | **0.002** | **0.009** | **38.028** | **1.244E-09** |
|  | **rs1786263** | **-0.019** | **0.002** | **-0.015** | **100.000** | **1.0259E-22** |
|  | **rs62103240** | **0.021** | **0.004** | **0.009** | **32.830** | **1.397E-08** |
|  | **rs4121583** | **0.012** | **0.002** | **0.009** | **34.810** | **4.624E-09** |
|  | **rs568267** | **0.012** | **0.002** | **0.008** | **30.752** | **2.226E-08** |
|  | **rs35073631** | **0.011** | **0.002** | **0.009** | **34.748** | **5.923E-09** |
|  | **rs12962050** | **0.015** | **0.002** | **0.011** | **58.523** | **1.5192E-14** |
|  | **rs7229520** | **-0.022** | **0.002** | **-0.017** | **125.440** | **9.2087E-29** |
|  | **rs7228151** | **-0.019** | **0.002** | **-0.012** | **64.698** | **3.1893E-15** |
|  | **rs9957318** | **0.019** | **0.002** | **0.014** | **87.423** | **1.02E-20** |
|  | **rs151123488** | **0.018** | **0.003** | **0.009** | **37.674** | **5.074E-10** |
|  | **rs60389750** | **-0.018** | **0.002** | **-0.012** | **69.444** | **1.0571E-16** |
|  | **rs79441499** | **-0.014** | **0.002** | **-0.011** | **52.753** | **9.6694E-13** |
|  | **rs11373507** | **-0.011** | **0.002** | **-0.008** | **31.923** | **1.122E-08** |
|  | **rs45474992** | **-0.062** | **0.005** | **-0.018** | **146.363** | **2.1672E-33** |
|  | **rs10421750** | **-0.015** | **0.002** | **-0.010** | **47.676** | **5.728E-12** |
|  | **rs16989695** | **-0.014** | **0.002** | **-0.011** | **53.521** | **1.9302E-13** |
|  | **rs12150907** | **-0.022** | **0.002** | **-0.014** | **83.266** | **7.2194E-20** |
|  | **rs10948** | **-0.025** | **0.002** | **-0.019** | **158.760** | **3.4277E-36** |
|  | **rs2607234** | **-0.030** | **0.004** | **-0.010** | **49.326** | **1.9962E-12** |
|  | **rs117203652** | **-0.035** | **0.006** | **-0.009** | **39.576** | **4.316E-10** |
|  | **rs4807472** | **-0.016** | **0.002** | **-0.012** | **62.410** | **8.1828E-15** |
|  | **rs12461874** | **-0.018** | **0.002** | **-0.013** | **74.288** | **1.2639E-17** |
|  | **rs116919274** | **0.027** | **0.005** | **0.009** | **34.707** | **3.523E-09** |
|  | **rs111901094** | **-0.025** | **0.003** | **-0.015** | **102.414** | **4.042E-24** |
|  | **rs75702986** | **-0.016** | **0.003** | **-0.010** | **42.510** | **3.142E-11** |
|  | **rs4252548** | **-0.075** | **0.007** | **-0.017** | **134.203** | **2.956E-31** |
|  | **rs8112948** | **-0.030** | **0.002** | **-0.020** | **182.250** | **4.2413E-42** |
|  | **rs350832** | **-0.017** | **0.002** | **-0.011** | **51.465** | **3.4411E-13** |
|  | **rs11260035** | **0.015** | **0.002** | **0.011** | **51.020** | **1.8561E-12** |
|  | **rs11672848** | **-0.017** | **0.002** | **-0.013** | **81.000** | **7.7339E-19** |
|  | **rs2287821** | **-0.015** | **0.002** | **-0.012** | **64.845** | **6.9534E-16** |
|  | **rs147110934** | **-0.072** | **0.006** | **-0.017** | **135.610** | **9.3886E-32** |
|  | **rs6054390** | **-0.019** | **0.002** | **-0.014** | **88.360** | **1.4451E-21** |
|  | **rs73125634** | **-0.020** | **0.002** | **-0.014** | **86.224** | **5.1145E-20** |
|  | **rs12185775** | **-0.017** | **0.003** | **-0.008** | **30.988** | **3.661E-08** |
|  | **rs112021215** | **-0.015** | **0.003** | **-0.009** | **34.574** | **5.992E-09** |
|  | **rs4815952** | **-0.016** | **0.002** | **-0.013** | **71.803** | **1.236E-16** |
|  | **rs35963161** | **-0.016** | **0.002** | **-0.012** | **68.280** | **7.4576E-16** |
|  | **rs77447813** | **0.022** | **0.003** | **0.010** | **43.405** | **3.2078E-11** |
|  | **rs2236096** | **0.018** | **0.002** | **0.012** | **61.248** | **1.288E-15** |
|  | **rs6142059** | **0.012** | **0.002** | **0.009** | **37.274** | **1.19E-09** |
|  | **rs143384** | **0.073** | **0.002** | **0.057** | **1456.025** | **1E-200** |
|  | **rs80132799** | **0.023** | **0.004** | **0.009** | **36.954** | **1.312E-09** |
|  | **rs6054491** | **-0.014** | **0.002** | **-0.010** | **41.661** | **2.152E-10** |
|  | **rs684905** | **-0.012** | **0.002** | **-0.009** | **38.571** | **7.1E-10** |
|  | **rs6082354** | **-0.024** | **0.002** | **-0.018** | **144.000** | **1.1899E-32** |
|  | **rs4287835** | **0.015** | **0.002** | **0.012** | **59.859** | **9.9449E-15** |
|  | **rs1291114** | **0.017** | **0.003** | **0.008** | **31.144** | **1.946E-08** |
|  | **rs57696574** | **0.017** | **0.002** | **0.013** | **74.823** | **4.5019E-18** |
|  | **rs34879158** | **-0.036** | **0.002** | **-0.025** | **272.250** | **1.5492E-63** |
|  | **rs6028716** | **-0.021** | **0.002** | **-0.014** | **91.116** | **4.5825E-22** |
|  | **rs6066122** | **0.013** | **0.002** | **0.008** | **30.490** | **1.755E-08** |
|  | **rs13037813** | **0.029** | **0.002** | **0.020** | **176.165** | **1.712E-39** |
|  | **rs73197345** | **0.021** | **0.003** | **0.011** | **56.787** | **3.5457E-14** |
|  | **rs12483401** | **-0.039** | **0.007** | **-0.009** | **33.364** | **9.221E-09** |
|  | **rs2230033** | **-0.027** | **0.002** | **-0.021** | **194.529** | **3.4882E-43** |
|  | **rs112153300** | **0.026** | **0.003** | **0.011** | **58.928** | **7.0534E-15** |
|  | **rs35631698** | **0.015** | **0.002** | **0.011** | **52.563** | **2.5912E-13** |
|  | **rs2212926** | **-0.022** | **0.002** | **-0.014** | **91.493** | **7.7482E-21** |
|  | **rs4818280** | **-0.012** | **0.002** | **-0.009** | **38.440** | **2.843E-10** |
|  | **rs9610447** | **0.015** | **0.002** | **0.010** | **47.736** | **5.2857E-12** |
|  | **rs5753518** | **0.024** | **0.003** | **0.011** | **53.778** | **5.0385E-13** |
|  | **rs7286917** | **0.017** | **0.002** | **0.011** | **55.276** | **5.1844E-14** |
|  | **rs41311445** | **-0.033** | **0.003** | **-0.015** | **105.063** | **4.7283E-24** |
|  | **rs5763821** | **0.019** | **0.002** | **0.014** | **91.203** | **1.0551E-21** |
|  | **rs6000886** | **0.013** | **0.002** | **0.010** | **42.903** | **5.8817E-11** |
|  | **rs8136517** | **0.027** | **0.004** | **0.010** | **46.870** | **6.5826E-12** |
|  | **rs165849** | **0.016** | **0.002** | **0.011** | **55.893** | **4.5698E-14** |
|  | **rs10453441** | **-0.014** | **0.002** | **-0.010** | **48.303** | **9.1012E-13** |
|  | **rs28379706** | **0.011** | **0.002** | **0.008** | **32.490** | **4.49E-09** |
| Whole body fat-free mass |  |  |  |  |  |  |
|  | **rs4648626** | **0.008** | **0.001** | **0.010** | **46.506** | **9.0991E-12** |
|  | **rs6665399** | **0.008** | **0.001** | **0.010** | **43.206** | **4.9E-11** |
|  | **rs67408364** | **0.014** | **0.002** | **0.014** | **88.159** | **6.0007E-21** |
|  | **rs2885697** | **-0.019** | **0.001** | **-0.021** | **207.375** | **5.1004E-47** |
|  | **rs892511** | **0.026** | **0.004** | **0.010** | **48.295** | **3.7E-12** |
|  | **rs7513326** | **-0.007** | **0.001** | **-0.008** | **31.048** | **2.5E-08** |
|  | **rs12729817** | **-0.008** | **0.001** | **-0.010** | **44.762** | **2.1999E-11** |
|  | **rs2281175** | **0.011** | **0.001** | **0.013** | **74.417** | **6.2994E-18** |
|  | **rs161799** | **-0.008** | **0.001** | **-0.009** | **37.522** | **9E-10** |
|  | **rs12041740** | **-0.015** | **0.001** | **-0.015** | **108.449** | **2.0999E-25** |
|  | **rs926436** | **0.010** | **0.002** | **0.008** | **30.388** | **3.5E-08** |
|  | **rs55800172** | **0.018** | **0.003** | **0.011** | **51.962** | **5.7003E-13** |
|  | **rs2568958** | **0.009** | **0.001** | **0.011** | **56.546** | **5.5005E-14** |
|  | **rs3845344** | **0.008** | **0.001** | **0.010** | **44.107** | **3.1003E-11** |
|  | **rs17363646** | **0.012** | **0.002** | **0.010** | **47.503** | **5.5005E-12** |
|  | **rs12047986** | **-0.008** | **0.001** | **-0.009** | **38.641** | **5.1E-10** |
|  | **rs116817990** | **-0.020** | **0.003** | **-0.009** | **33.943** | **5.6999E-09** |
|  | **rs12070699** | **-0.009** | **0.001** | **-0.011** | **54.531** | **1.5E-13** |
|  | **rs6693481** | **-0.008** | **0.001** | **-0.009** | **37.617** | **8.6E-10** |
|  | **rs12072845** | **-0.014** | **0.001** | **-0.016** | **121.647** | **2.8003E-28** |
|  | **rs2789366** | **-0.009** | **0.001** | **-0.010** | **49.981** | **1.5999E-12** |
|  | **rs284315** | **-0.008** | **0.001** | **-0.009** | **38.715** | **4.9E-10** |
|  | **rs2092322** | **-0.010** | **0.001** | **-0.011** | **59.128** | **1.5E-14** |
|  | **rs11578046** | **-0.013** | **0.001** | **-0.015** | **96.106** | **1.1E-22** |
|  | **rs4360494** | **-0.010** | **0.001** | **-0.012** | **62.939** | **2.0999E-15** |
|  | **rs2104449** | **0.011** | **0.001** | **0.011** | **60.011** | **9.3994E-15** |
|  | **rs527248** | **0.026** | **0.002** | **0.025** | **282.171** | **2.4998E-63** |
|  | **rs12731187** | **-0.008** | **0.001** | **-0.009** | **35.517** | **2.5E-09** |
|  | **rs2970592** | **0.009** | **0.001** | **0.011** | **53.233** | **2.9999E-13** |
|  | **rs35492502** | **0.010** | **0.001** | **0.011** | **53.935** | **2.0999E-13** |
|  | **rs75786059** | **0.014** | **0.002** | **0.013** | **77.004** | **1.6998E-18** |
|  | **rs11263853** | **0.009** | **0.002** | **0.009** | **34.253** | **4.8E-09** |
|  | **rs4926542** | **-0.010** | **0.001** | **-0.011** | **53.675** | **2.3999E-13** |
|  | **rs12095997** | **0.023** | **0.002** | **0.015** | **107.985** | **2.7002E-25** |
|  | **rs12140153** | **-0.017** | **0.002** | **-0.012** | **61.800** | **3.8001E-15** |
|  | **rs17277008** | **0.014** | **0.001** | **0.016** | **116.081** | **4.6005E-27** |
|  | **rs2678204** | **0.012** | **0.001** | **0.014** | **85.435** | **2.3999E-20** |
|  | **rs11240565** | **0.013** | **0.001** | **0.015** | **105.645** | **8.8004E-25** |
|  | **rs148662000** | **-0.015** | **0.003** | **-0.009** | **35.175** | **3E-09** |
|  | **rs6681795** | **0.015** | **0.001** | **0.015** | **104.480** | **1.5999E-24** |
|  | **rs34517439** | **0.037** | **0.002** | **0.029** | **378.083** | **3.2999E-84** |
|  | **rs77848106** | **-0.009** | **0.001** | **-0.010** | **43.674** | **3.9003E-11** |
|  | **rs60804050** | **-0.010** | **0.001** | **-0.010** | **50.112** | **1.5E-12** |
|  | **rs11205354** | **-0.008** | **0.001** | **-0.009** | **39.255** | **3.7E-10** |
|  | **rs76798800** | **0.023** | **0.001** | **0.024** | **263.995** | **2.2999E-59** |
|  | **rs77159542** | **0.014** | **0.002** | **0.009** | **33.142** | **8.6E-09** |
|  | **rs1040457** | **0.015** | **0.001** | **0.017** | **132.125** | **1.3999E-30** |
|  | **rs2615075** | **0.010** | **0.001** | **0.011** | **58.530** | **1.9999E-14** |
|  | **rs6743060** | **0.034** | **0.002** | **0.030** | **421.543** | **1.1E-93** |
|  | **rs6721191** | **-0.008** | **0.001** | **-0.009** | **36.001** | **2E-09** |
|  | **rs1374370** | **0.010** | **0.001** | **0.011** | **57.445** | **3.5003E-14** |
|  | **rs2140046** | **-0.012** | **0.001** | **-0.014** | **83.014** | **8.1997E-20** |
|  | **rs116337081** | **0.014** | **0.002** | **0.009** | **34.830** | **3.6E-09** |
|  | **rs17443541** | **-0.011** | **0.002** | **-0.010** | **47.388** | **5.7996E-12** |
|  | **rs11684531** | **-0.011** | **0.002** | **-0.009** | **38.143** | **6.5999E-10** |
|  | **rs3116201** | **-0.018** | **0.002** | **-0.013** | **74.198** | **7.1007E-18** |
|  | **rs3772051** | **-0.009** | **0.001** | **-0.009** | **33.851** | **5.9E-09** |
|  | **rs4676442** | **0.007** | **0.001** | **0.009** | **33.019** | **9.1E-09** |
|  | **rs1260326** | **0.019** | **0.001** | **0.022** | **224.188** | **1.1E-50** |
|  | **rs10172196** | **0.011** | **0.001** | **0.012** | **63.847** | **1.2999E-15** |
|  | **rs1805165** | **-0.010** | **0.001** | **-0.011** | **53.604** | **2.4998E-13** |
|  | **rs71423263** | **0.012** | **0.002** | **0.010** | **45.532** | **1.5E-11** |
|  | **rs13392079** | **-0.008** | **0.001** | **-0.009** | **34.724** | **3.8E-09** |
|  | **rs72885917** | **-0.020** | **0.001** | **-0.021** | **204.451** | **2.1999E-46** |
|  | **rs17400325** | **0.022** | **0.003** | **0.010** | **48.245** | **3.8001E-12** |
|  | **rs13430869** | **0.014** | **0.001** | **0.014** | **91.084** | **1.3999E-21** |
|  | **rs1478575** | **0.018** | **0.001** | **0.019** | **172.898** | **1.6998E-39** |
|  | **rs1542224** | **0.013** | **0.001** | **0.014** | **85.795** | **1.9999E-20** |
|  | **rs10202845** | **-0.018** | **0.002** | **-0.013** | **79.723** | **4.3003E-19** |
|  | **rs2920974** | **0.007** | **0.001** | **0.008** | **32.681** | **1.1E-08** |
|  | **rs116211567** | **-0.018** | **0.002** | **-0.012** | **61.165** | **5.2E-15** |
|  | **rs11545482** | **-0.032** | **0.004** | **-0.011** | **52.453** | **4.4005E-13** |
|  | **rs3771382** | **-0.012** | **0.001** | **-0.014** | **90.462** | **1.9002E-21** |
|  | **rs6719296** | **0.009** | **0.001** | **0.011** | **50.571** | **1.1E-12** |
|  | **rs6747657** | **0.008** | **0.001** | **0.009** | **34.326** | **4.7E-09** |
|  | **rs10803955** | **-0.011** | **0.001** | **-0.013** | **78.945** | **6.4003E-19** |
|  | **rs12694042** | **-0.008** | **0.001** | **-0.010** | **43.713** | **3.8001E-11** |
|  | **rs17246129** | **0.013** | **0.001** | **0.014** | **86.499** | **1.3999E-20** |
|  | **rs2197563** | **0.010** | **0.001** | **0.012** | **66.312** | **3.8001E-16** |
|  | **rs10170971** | **-0.009** | **0.001** | **-0.011** | **58.009** | **2.6002E-14** |
|  | **rs77165542** | **-0.058** | **0.003** | **-0.026** | **298.600** | **6.5993E-67** |
|  | **rs112544217** | **-0.027** | **0.004** | **-0.009** | **38.766** | **4.8E-10** |
|  | **rs12713004** | **0.017** | **0.001** | **0.018** | **152.982** | **3.9003E-35** |
|  | **rs41458449** | **-0.011** | **0.002** | **-0.009** | **36.079** | **1.9E-09** |
|  | **rs6743107** | **0.008** | **0.001** | **0.008** | **32.287** | **1.3E-08** |
|  | **rs35651070** | **-0.010** | **0.002** | **-0.008** | **32.855** | **9.9001E-09** |
|  | **rs10188231** | **-0.010** | **0.002** | **-0.009** | **39.876** | **2.7E-10** |
|  | **rs1064213** | **0.011** | **0.001** | **0.014** | **85.965** | **1.8001E-20** |
|  | **rs1047891** | **0.017** | **0.001** | **0.019** | **161.256** | **6.0007E-37** |
|  | **rs11689727** | **-0.011** | **0.001** | **-0.012** | **69.661** | **7E-17** |
|  | **rs115179432** | **-0.022** | **0.002** | **-0.014** | **84.201** | **4.4999E-20** |
|  | **rs59985551** | **-0.018** | **0.001** | **-0.019** | **155.962** | **8.6E-36** |
|  | **rs752070** | **0.011** | **0.002** | **0.009** | **37.708** | **8.1999E-10** |
|  | **rs4504126** | **0.025** | **0.004** | **0.010** | **43.452** | **4.3003E-11** |
|  | **rs6779752** | **-0.013** | **0.001** | **-0.015** | **96.590** | **8.4996E-23** |
|  | **rs843374** | **-0.011** | **0.001** | **-0.013** | **79.268** | **5.4001E-19** |
|  | **rs62246311** | **0.013** | **0.002** | **0.009** | **40.312** | **2.2E-10** |
|  | **rs4677153** | **-0.007** | **0.001** | **-0.008** | **32.511** | **1.2E-08** |
|  | **rs4858940** | **0.016** | **0.002** | **0.012** | **68.613** | **1.2001E-16** |
|  | **rs34693680** | **0.014** | **0.002** | **0.012** | **62.105** | **3.2999E-15** |
|  | **rs11709402** | **0.010** | **0.001** | **0.011** | **51.530** | **7.1007E-13** |
|  | **rs4635681** | **0.011** | **0.002** | **0.010** | **41.570** | **1.1E-10** |
|  | **rs357486** | **0.010** | **0.001** | **0.012** | **65.222** | **6.7004E-16** |
|  | **rs13085472** | **-0.008** | **0.001** | **-0.009** | **39.083** | **4.1E-10** |
|  | **rs9882731** | **-0.012** | **0.001** | **-0.014** | **83.576** | **6.0996E-20** |
|  | **rs73052033** | **-0.015** | **0.002** | **-0.014** | **92.992** | **5.2E-22** |
|  | **rs6772164** | **0.008** | **0.001** | **0.009** | **39.603** | **3.1E-10** |
|  | **rs2270894** | **-0.017** | **0.002** | **-0.016** | **119.031** | **1E-27** |
|  | **rs6781248** | **0.008** | **0.001** | **0.009** | **36.810** | **1.3E-09** |
|  | **rs7647657** | **-0.011** | **0.001** | **-0.013** | **78.252** | **9.0991E-19** |
|  | **rs11925245** | **-0.010** | **0.002** | **-0.009** | **37.318** | **1E-09** |
|  | **rs1910466** | **-0.008** | **0.001** | **-0.009** | **37.453** | **9.4001E-10** |
|  | **rs34345690** | **0.008** | **0.001** | **0.008** | **30.401** | **3.5E-08** |
|  | **rs434072** | **-0.008** | **0.001** | **-0.008** | **30.120** | **4.1E-08** |
|  | **rs2365363** | **0.007** | **0.001** | **0.008** | **29.961** | **4.4E-08** |
|  | **rs11712872** | **0.017** | **0.002** | **0.013** | **75.168** | **4.3003E-18** |
|  | **rs77344209** | **0.010** | **0.001** | **0.010** | **46.004** | **1.2001E-11** |
|  | **rs4974223** | **0.016** | **0.002** | **0.011** | **59.856** | **1E-14** |
|  | **rs485554** | **0.017** | **0.001** | **0.019** | **163.702** | **1.8001E-37** |
|  | **rs73175572** | **0.028** | **0.002** | **0.021** | **197.872** | **6.0996E-45** |
|  | **rs7619139** | **0.012** | **0.001** | **0.014** | **89.295** | **3.4002E-21** |
|  | **rs6800021** | **0.014** | **0.001** | **0.016** | **118.121** | **1.5999E-27** |
|  | **rs60385590** | **0.009** | **0.001** | **0.010** | **41.766** | **1E-10** |
|  | **rs10511111** | **0.009** | **0.001** | **0.010** | **42.284** | **7.8995E-11** |
|  | **rs6762578** | **0.016** | **0.001** | **0.016** | **110.197** | **8.9002E-26** |
|  | **rs9853018** | **0.030** | **0.001** | **0.036** | **578.967** | **6.295E-128** |
|  | **rs111391498** | **-0.026** | **0.003** | **-0.013** | **78.841** | **6.7004E-19** |
|  | **rs4132132** | **0.009** | **0.001** | **0.010** | **47.795** | **4.7E-12** |
|  | **rs10020631** | **-0.008** | **0.001** | **-0.009** | **33.239** | **8.1999E-09** |
|  | **rs17011108** | **0.008** | **0.001** | **0.008** | **31.188** | **2.3E-08** |
|  | **rs1296328** | **-0.009** | **0.001** | **-0.010** | **47.875** | **4.4999E-12** |
|  | **rs3990738** | **-0.008** | **0.001** | **-0.009** | **38.144** | **6.5999E-10** |
|  | **rs981002** | **-0.011** | **0.001** | **-0.011** | **56.624** | **5.3003E-14** |
|  | **rs1841738** | **-0.011** | **0.001** | **-0.013** | **80.313** | **3.1996E-19** |
|  | **rs2101975** | **-0.016** | **0.001** | **-0.019** | **170.000** | **7.3995E-39** |
|  | **rs1028197** | **0.008** | **0.001** | **0.008** | **30.091** | **4.1E-08** |
|  | **rs9985795** | **-0.007** | **0.001** | **-0.009** | **34.167** | **5.1E-09** |
|  | **rs34227797** | **0.008** | **0.001** | **0.009** | **37.469** | **9.2999E-10** |
|  | **rs7683836** | **-0.007** | **0.001** | **-0.008** | **30.460** | **3.4E-08** |
|  | **rs798759** | **-0.010** | **0.001** | **-0.012** | **60.676** | **6.7004E-15** |
|  | **rs2968669** | **0.007** | **0.001** | **0.009** | **33.125** | **8.6E-09** |
|  | **rs7671110** | **-0.032** | **0.002** | **-0.028** | **350.803** | **2.8003E-78** |
|  | **rs10939792** | **0.008** | **0.001** | **0.009** | **35.077** | **3.2E-09** |
|  | **rs17556750** | **0.012** | **0.001** | **0.013** | **75.495** | **3.7E-18** |
|  | **rs6821305** | **0.013** | **0.001** | **0.015** | **107.583** | **3.2999E-25** |
|  | **rs34848742** | **-0.015** | **0.002** | **-0.014** | **94.656** | **2.2999E-22** |
|  | **rs16844418** | **-0.012** | **0.002** | **-0.010** | **49.610** | **1.9002E-12** |
|  | **rs73213484** | **-0.012** | **0.002** | **-0.010** | **45.473** | **1.5E-11** |
|  | **rs10938397** | **0.012** | **0.001** | **0.014** | **90.615** | **1.6998E-21** |
|  | **rs10434434** | **-0.014** | **0.002** | **-0.012** | **62.734** | **2.3999E-15** |
|  | **rs13125082** | **-0.008** | **0.001** | **-0.008** | **30.478** | **3.4E-08** |
|  | **rs11937249** | **-0.008** | **0.001** | **-0.009** | **38.653** | **5.1E-10** |
|  | **rs4240326** | **-0.021** | **0.001** | **-0.025** | **290.596** | **3.7E-65** |
|  | **rs72703409** | **-0.015** | **0.003** | **-0.009** | **35.943** | **2E-09** |
|  | **rs10222924** | **-0.010** | **0.001** | **-0.010** | **49.498** | **1.9999E-12** |
|  | **rs140493137** | **0.019** | **0.003** | **0.011** | **55.164** | **1.1E-13** |
|  | **rs111598585** | **-0.009** | **0.002** | **-0.008** | **31.468** | **0.00000002** |
|  | **rs7706886** | **0.013** | **0.001** | **0.013** | **79.008** | **6.2001E-19** |
|  | **rs12657771** | **-0.013** | **0.001** | **-0.016** | **114.654** | **9.3994E-27** |
|  | **rs62372052** | **0.027** | **0.002** | **0.020** | **179.313** | **6.7999E-41** |
|  | **rs4865956** | **-0.012** | **0.001** | **-0.013** | **78.695** | **7.2996E-19** |
|  | **rs182224** | **0.013** | **0.002** | **0.009** | **34.351** | **4.6E-09** |
|  | **rs9800418** | **0.008** | **0.001** | **0.009** | **33.303** | **7.9001E-09** |
|  | **rs7728690** | **-0.011** | **0.001** | **-0.013** | **74.862** | **5.0004E-18** |
|  | **rs36695** | **-0.009** | **0.001** | **-0.011** | **53.735** | **2.2999E-13** |
|  | **rs13185520** | **-0.007** | **0.001** | **-0.009** | **34.057** | **5.4E-09** |
|  | **rs3822742** | **0.013** | **0.001** | **0.015** | **109.014** | **1.5999E-25** |
|  | **rs3853474** | **-0.008** | **0.001** | **-0.009** | **36.813** | **1.3E-09** |
|  | **rs6874142** | **0.018** | **0.002** | **0.013** | **77.459** | **1.3999E-18** |
|  | **rs12188627** | **-0.010** | **0.001** | **-0.012** | **69.877** | **6.2994E-17** |
|  | **rs141729694** | **0.019** | **0.002** | **0.012** | **65.919** | **4.7E-16** |
|  | **rs9327336** | **0.009** | **0.001** | **0.010** | **44.060** | **3.1996E-11** |
|  | **rs4912650** | **-0.009** | **0.001** | **-0.010** | **43.715** | **3.8001E-11** |
|  | **rs111365325** | **-0.014** | **0.001** | **-0.015** | **96.346** | **9.6006E-23** |
|  | **rs2578557** | **-0.009** | **0.001** | **-0.011** | **53.304** | **2.9E-13** |
|  | **rs2307111** | **-0.016** | **0.001** | **-0.019** | **167.930** | **2.0999E-38** |
|  | **rs6235** | **0.016** | **0.001** | **0.017** | **128.588** | **8.3004E-30** |
|  | **rs2611732** | **0.008** | **0.001** | **0.009** | **37.461** | **9.2999E-10** |
|  | **rs2952615** | **-0.012** | **0.001** | **-0.014** | **87.393** | **8.9002E-21** |
|  | **rs1582931** | **-0.019** | **0.001** | **-0.022** | **224.655** | **8.6996E-51** |
|  | **rs247008** | **0.013** | **0.001** | **0.014** | **89.170** | **3.6E-21** |
|  | **rs17115481** | **-0.009** | **0.001** | **-0.010** | **41.374** | **1.3E-10** |
|  | **rs254963** | **-0.009** | **0.001** | **-0.010** | **47.136** | **6.5993E-12** |
|  | **rs7731023** | **0.009** | **0.001** | **0.010** | **46.819** | **7.8001E-12** |
|  | **rs9291926** | **-0.012** | **0.001** | **-0.014** | **92.703** | **6.0996E-22** |
|  | **rs365352** | **-0.015** | **0.001** | **-0.015** | **105.545** | **9.3004E-25** |
|  | **rs244711** | **0.017** | **0.001** | **0.017** | **136.952** | **1.2001E-31** |
|  | **rs9654451** | **0.011** | **0.002** | **0.009** | **36.710** | **1.4E-09** |
|  | **rs72754922** | **-0.016** | **0.003** | **-0.009** | **37.505** | **9.1E-10** |
|  | **rs695922** | **-0.010** | **0.002** | **-0.009** | **36.346** | **1.7E-09** |
|  | **rs505575** | **-0.009** | **0.001** | **-0.010** | **44.436** | **2.6002E-11** |
|  | **rs31211** | **-0.013** | **0.001** | **-0.014** | **83.250** | **7.1995E-20** |
|  | **rs2422054** | **0.008** | **0.001** | **0.009** | **38.629** | **5.1E-10** |
|  | **rs4282339** | **-0.019** | **0.002** | **-0.018** | **153.912** | **2.3999E-35** |
|  | **rs55758152** | **0.010** | **0.001** | **0.011** | **53.311** | **2.8003E-13** |
|  | **rs10498672** | **0.010** | **0.002** | **0.009** | **40.860** | **1.6E-10** |
|  | **rs115461836** | **0.021** | **0.003** | **0.010** | **48.032** | **4.1995E-12** |
|  | **rs41271299** | **0.042** | **0.003** | **0.022** | **222.716** | **2.2999E-50** |
|  | **rs35612982** | **-0.009** | **0.002** | **-0.008** | **32.667** | **1.1E-08** |
|  | **rs578366** | **-0.010** | **0.001** | **-0.012** | **66.484** | **3.5003E-16** |
|  | **rs1179905** | **0.010** | **0.002** | **0.010** | **42.724** | **6.2994E-11** |
|  | **rs6902789** | **0.011** | **0.001** | **0.012** | **68.809** | **1.1E-16** |
|  | **rs9491201** | **0.010** | **0.002** | **0.009** | **32.943** | **9.4999E-09** |
|  | **rs9398171** | **0.021** | **0.001** | **0.023** | **232.396** | **1.8001E-52** |
|  | **rs12213409** | **-0.007** | **0.001** | **-0.008** | **29.820** | **4.7E-08** |
|  | **rs6570509** | **-0.014** | **0.001** | **-0.015** | **107.262** | **3.9003E-25** |
|  | **rs73013411** | **0.013** | **0.002** | **0.011** | **51.066** | **8.9002E-13** |
|  | **rs76307059** | **-0.019** | **0.003** | **-0.009** | **39.601** | **3.1E-10** |
|  | **rs11243202** | **0.018** | **0.001** | **0.022** | **216.152** | **6.2994E-49** |
|  | **rs9461246** | **-0.018** | **0.002** | **-0.014** | **86.311** | **1.5E-20** |
|  | **rs2524137** | **-0.021** | **0.001** | **-0.023** | **231.870** | **2.2999E-52** |
|  | **rs2744956** | **0.038** | **0.002** | **0.031** | **447.209** | **2.9E-99** |
|  | **rs12193797** | **-0.019** | **0.002** | **-0.015** | **103.305** | **2.9E-24** |
|  | **rs1418433** | **-0.009** | **0.001** | **-0.010** | **45.769** | **1.2999E-11** |
|  | **rs240113** | **-0.008** | **0.001** | **-0.010** | **41.661** | **1.1E-10** |
|  | **rs9372837** | **0.015** | **0.001** | **0.018** | **147.299** | **6.7999E-34** |
|  | **rs7740107** | **-0.029** | **0.001** | **-0.031** | **430.706** | **1.1E-95** |
|  | **rs7755185** | **0.009** | **0.001** | **0.010** | **41.829** | **1E-10** |
|  | **rs9380508** | **0.012** | **0.001** | **0.012** | **68.291** | **1.3999E-16** |
|  | **rs72892910** | **0.021** | **0.002** | **0.019** | **158.391** | **2.4998E-36** |
|  | **rs1266874** | **0.008** | **0.001** | **0.010** | **41.624** | **1.1E-10** |
|  | **rs9480933** | **-0.013** | **0.001** | **-0.015** | **100.974** | **9.3004E-24** |
|  | **rs603321** | **-0.012** | **0.001** | **-0.012** | **63.528** | **1.5999E-15** |
|  | **rs10945540** | **-0.009** | **0.001** | **-0.011** | **50.606** | **1.1E-12** |
|  | **rs9365939** | **-0.007** | **0.001** | **-0.009** | **35.519** | **2.5E-09** |
|  | **rs62439025** | **0.011** | **0.002** | **0.009** | **40.642** | **1.8E-10** |
|  | **rs9379084** | **-0.015** | **0.002** | **-0.011** | **57.915** | **2.7002E-14** |
|  | **rs2071286** | **-0.021** | **0.002** | **-0.019** | **171.121** | **4.1995E-39** |
|  | **rs9394166** | **0.009** | **0.001** | **0.010** | **48.238** | **3.8001E-12** |
|  | **rs12209223** | **0.015** | **0.002** | **0.011** | **53.526** | **2.6002E-13** |
|  | **rs9350850** | **0.023** | **0.002** | **0.015** | **104.560** | **1.5E-24** |
|  | **rs12200061** | **-0.007** | **0.001** | **-0.009** | **34.895** | **3.5E-09** |
|  | **rs6900690** | **0.008** | **0.001** | **0.010** | **41.601** | **1.1E-10** |
|  | **rs3853252** | **0.015** | **0.001** | **0.018** | **149.983** | **1.6998E-34** |
|  | **rs34776209** | **-0.017** | **0.001** | **-0.018** | **141.113** | **1.5E-32** |
|  | **rs4729085** | **-0.016** | **0.002** | **-0.013** | **76.142** | **2.6002E-18** |
|  | **rs7780752** | **0.012** | **0.001** | **0.014** | **92.930** | **5.4001E-22** |
|  | **rs12375196** | **0.009** | **0.001** | **0.011** | **56.215** | **6.4998E-14** |
|  | **rs73158212** | **-0.008** | **0.002** | **-0.008** | **31.064** | **2.5E-08** |
|  | **rs114949263** | **-0.013** | **0.002** | **-0.010** | **44.535** | **2.4998E-11** |
|  | **rs10282707** | **0.008** | **0.001** | **0.009** | **39.302** | **3.6E-10** |
|  | **rs2122823** | **0.010** | **0.002** | **0.010** | **44.962** | **1.9999E-11** |
|  | **rs508347** | **-0.012** | **0.001** | **-0.013** | **74.000** | **7.8001E-18** |
|  | **rs10950207** | **0.008** | **0.001** | **0.010** | **42.887** | **5.7996E-11** |
|  | **rs13240065** | **0.018** | **0.002** | **0.014** | **94.164** | **2.9E-22** |
|  | **rs62621812** | **0.043** | **0.005** | **0.014** | **88.204** | **5.9007E-21** |
|  | **rs12533548** | **0.008** | **0.001** | **0.009** | **38.827** | **4.6E-10** |
|  | **rs2533879** | **-0.021** | **0.001** | **-0.023** | **231.143** | **3.4002E-52** |
|  | **rs836511** | **0.010** | **0.002** | **0.010** | **45.014** | **1.9999E-11** |
|  | **rs37964** | **0.008** | **0.001** | **0.010** | **45.786** | **1.2999E-11** |
|  | **rs77760034** | **-0.019** | **0.002** | **-0.012** | **63.825** | **1.3999E-15** |
|  | **rs10269774** | **0.026** | **0.001** | **0.029** | **391.147** | **4.7E-87** |
|  | **rs62466118** | **-0.025** | **0.004** | **-0.010** | **41.118** | **1.4E-10** |
|  | **rs62460525** | **0.014** | **0.002** | **0.008** | **31.820** | **1.7E-08** |
|  | **rs2740761** | **0.009** | **0.002** | **0.008** | **31.832** | **1.7E-08** |
|  | **rs6972291** | **-0.011** | **0.002** | **-0.010** | **46.845** | **7.7002E-12** |
|  | **rs2293177** | **0.007** | **0.001** | **0.008** | **30.789** | **2.9E-08** |
|  | **rs6946415** | **0.019** | **0.001** | **0.022** | **216.130** | **6.2994E-49** |
|  | **rs6946419** | **-0.008** | **0.001** | **-0.009** | **37.974** | **7.2E-10** |
|  | **rs2881198** | **-0.012** | **0.001** | **-0.015** | **96.757** | **7.8001E-23** |
|  | **rs10899736** | **-0.010** | **0.001** | **-0.012** | **66.066** | **4.4005E-16** |
|  | **rs236628** | **0.009** | **0.002** | **0.009** | **38.081** | **6.8E-10** |
|  | **rs10248298** | **0.011** | **0.001** | **0.013** | **78.377** | **8.4996E-19** |
|  | **rs822549** | **0.012** | **0.001** | **0.013** | **72.891** | **1.3999E-17** |
|  | **rs76364830** | **-0.022** | **0.003** | **-0.013** | **76.359** | **2.3999E-18** |
|  | **rs4439140** | **-0.008** | **0.001** | **-0.010** | **43.304** | **4.7E-11** |
|  | **rs11997525** | **0.015** | **0.002** | **0.014** | **84.450** | **3.9003E-20** |
|  | **rs72656010** | **-0.037** | **0.002** | **-0.030** | **396.737** | **2.8003E-88** |
|  | **rs61729527** | **-0.024** | **0.003** | **-0.013** | **72.281** | **1.9002E-17** |
|  | **rs7842996** | **0.019** | **0.001** | **0.021** | **192.827** | **7.7002E-44** |
|  | **rs894360** | **-0.019** | **0.001** | **-0.022** | **216.335** | **5.7003E-49** |
|  | **rs7843128** | **-0.008** | **0.001** | **-0.009** | **40.498** | **2E-10** |
|  | **rs2979649** | **0.010** | **0.002** | **0.009** | **34.808** | **3.6E-09** |
|  | **rs13264909** | **-0.009** | **0.001** | **-0.010** | **46.429** | **9.4995E-12** |
|  | **rs16916881** | **-0.010** | **0.001** | **-0.011** | **51.017** | **9.2003E-13** |
|  | **rs11782103** | **-0.013** | **0.001** | **-0.013** | **82.768** | **9.2003E-20** |
|  | **rs62515437** | **0.019** | **0.001** | **0.019** | **168.683** | **1.3999E-38** |
|  | **rs55674305** | **-0.011** | **0.001** | **-0.012** | **66.752** | **3.1003E-16** |
|  | **rs2721938** | **-0.012** | **0.001** | **-0.014** | **91.237** | **1.2999E-21** |
|  | **rs12156265** | **0.008** | **0.001** | **0.010** | **41.647** | **1.1E-10** |
|  | **rs13272451** | **0.009** | **0.001** | **0.011** | **55.617** | **8.8004E-14** |
|  | **rs11785562** | **0.012** | **0.002** | **0.011** | **57.945** | **2.7002E-14** |
|  | **rs3925** | **-0.011** | **0.001** | **-0.011** | **55.035** | **1.2001E-13** |
|  | **rs17828687** | **-0.009** | **0.001** | **-0.011** | **51.589** | **6.7999E-13** |
|  | **rs10283100** | **0.028** | **0.003** | **0.015** | **105.662** | **8.6996E-25** |
|  | **rs13271368** | **-0.015** | **0.001** | **-0.015** | **99.436** | **1.9999E-23** |
|  | **rs7815955** | **-0.015** | **0.002** | **-0.015** | **96.778** | **7.8001E-23** |
|  | **rs6984820** | **-0.010** | **0.001** | **-0.012** | **64.744** | **8.4996E-16** |
|  | **rs1412234** | **0.011** | **0.001** | **0.012** | **67.049** | **2.6002E-16** |
|  | **rs10780905** | **-0.010** | **0.001** | **-0.012** | **60.242** | **8.4004E-15** |
|  | **rs663344** | **-0.015** | **0.002** | **-0.014** | **84.577** | **3.7E-20** |
|  | **rs10119967** | **0.016** | **0.002** | **0.015** | **101.884** | **5.9007E-24** |
|  | **rs7033487** | **-0.023** | **0.002** | **-0.022** | **225.989** | **4.4999E-51** |
|  | **rs56141370** | **0.013** | **0.002** | **0.010** | **41.743** | **1E-10** |
|  | **rs13299559** | **-0.010** | **0.001** | **-0.012** | **65.720** | **5.2E-16** |
|  | **rs11794152** | **0.010** | **0.001** | **0.012** | **67.258** | **2.3999E-16** |
|  | **rs12553221** | **0.007** | **0.001** | **0.008** | **31.311** | **2.2E-08** |
|  | **rs11142700** | **-0.008** | **0.001** | **-0.009** | **39.487** | **3.3E-10** |
|  | **rs28620532** | **0.018** | **0.001** | **0.021** | **193.190** | **6.4003E-44** |
|  | **rs1056747** | **-0.008** | **0.001** | **-0.010** | **43.927** | **3.4002E-11** |
|  | **rs10881583** | **-0.009** | **0.001** | **-0.009** | **39.433** | **3.4E-10** |
|  | **rs2274116** | **-0.008** | **0.001** | **-0.009** | **37.689** | **8.3E-10** |
|  | **rs1927635** | **0.009** | **0.001** | **0.010** | **47.687** | **5.0004E-12** |
|  | **rs1928850** | **0.014** | **0.002** | **0.009** | **37.323** | **1E-09** |
|  | **rs968821** | **-0.011** | **0.001** | **-0.013** | **72.063** | **2.0999E-17** |
|  | **rs10979612** | **0.019** | **0.002** | **0.012** | **61.284** | **4.9E-15** |
|  | **rs1008158** | **0.008** | **0.001** | **0.010** | **41.585** | **1.1E-10** |
|  | **rs7095768** | **0.008** | **0.001** | **0.008** | **30.889** | **2.7E-08** |
|  | **rs3740591** | **0.011** | **0.001** | **0.013** | **81.518** | **1.6998E-19** |
|  | **rs1268947** | **0.012** | **0.002** | **0.010** | **42.060** | **8.9002E-11** |
|  | **rs117543413** | **-0.038** | **0.005** | **-0.012** | **64.031** | **1.2001E-15** |
|  | **rs11245450** | **-0.010** | **0.001** | **-0.012** | **65.147** | **7E-16** |
|  | **rs11014285** | **0.019** | **0.002** | **0.017** | **128.071** | **1.1E-29** |
|  | **rs572474** | **0.010** | **0.002** | **0.009** | **35.951** | **2E-09** |
|  | **rs7080472** | **0.013** | **0.001** | **0.015** | **106.702** | **5.2E-25** |
|  | **rs2265309** | **-0.012** | **0.001** | **-0.015** | **99.971** | **1.5E-23** |
|  | **rs11593630** | **-0.009** | **0.001** | **-0.010** | **45.962** | **1.2001E-11** |
|  | **rs6585827** | **0.011** | **0.001** | **0.014** | **84.512** | **3.8001E-20** |
|  | **rs1421035** | **0.008** | **0.001** | **0.009** | **33.277** | **8E-09** |
|  | **rs1846221** | **-0.010** | **0.002** | **-0.009** | **32.906** | **9.7E-09** |
|  | **rs12764498** | **-0.015** | **0.002** | **-0.012** | **63.563** | **1.5999E-15** |
|  | **rs2807742** | **-0.009** | **0.001** | **-0.009** | **37.618** | **8.6E-10** |
|  | **rs10995366** | **-0.010** | **0.001** | **-0.010** | **46.076** | **1.1E-11** |
|  | **rs11001399** | **-0.011** | **0.001** | **-0.013** | **79.360** | **5.2E-19** |
|  | **rs4980067** | **-0.011** | **0.001** | **-0.013** | **81.405** | **1.8001E-19** |
|  | **rs7912286** | **-0.009** | **0.001** | **-0.010** | **45.812** | **1.2999E-11** |
|  | **rs73601548** | **0.014** | **0.002** | **0.011** | **52.287** | **4.7995E-13** |
|  | **rs12146350** | **-0.008** | **0.001** | **-0.009** | **40.223** | **2.3E-10** |
|  | **rs1657222** | **0.008** | **0.001** | **0.009** | **40.753** | **1.7E-10** |
|  | **rs224048** | **-0.007** | **0.001** | **-0.009** | **34.720** | **3.8E-09** |
|  | **rs10883560** | **0.017** | **0.001** | **0.020** | **186.871** | **1.5E-42** |
|  | **rs61878760** | **0.015** | **0.002** | **0.010** | **44.292** | **2.8003E-11** |
|  | **rs112758380** | **0.017** | **0.003** | **0.009** | **40.380** | **2.1E-10** |
|  | **rs676105** | **0.014** | **0.001** | **0.015** | **101.338** | **7.8001E-24** |
|  | **rs294382** | **-0.008** | **0.001** | **-0.008** | **30.139** | **0.00000004** |
|  | **rs35251247** | **0.011** | **0.001** | **0.012** | **61.777** | **3.8001E-15** |
|  | **rs7129320** | **-0.023** | **0.002** | **-0.021** | **192.058** | **1.1E-43** |
|  | **rs667515** | **-0.010** | **0.001** | **-0.012** | **65.466** | **5.9007E-16** |
|  | **rs74048171** | **-0.010** | **0.001** | **-0.011** | **53.092** | **3.1996E-13** |
|  | **rs11030112** | **0.017** | **0.001** | **0.019** | **157.721** | **3.6E-36** |
|  | **rs4268495** | **-0.009** | **0.001** | **-0.010** | **44.745** | **2.1999E-11** |
|  | **rs34345560** | **0.009** | **0.002** | **0.008** | **32.433** | **1.2E-08** |
|  | **rs12795042** | **0.007** | **0.001** | **0.008** | **31.262** | **2.3E-08** |
|  | **rs11042725** | **-0.012** | **0.001** | **-0.014** | **89.488** | **3.1003E-21** |
|  | **rs1228024** | **-0.011** | **0.001** | **-0.012** | **67.520** | **2.0999E-16** |
|  | **rs56207600** | **0.012** | **0.002** | **0.009** | **37.414** | **9.6E-10** |
|  | **rs35506085** | **-0.020** | **0.002** | **-0.018** | **151.501** | **8.1003E-35** |
|  | **rs11021307** | **0.007** | **0.001** | **0.008** | **30.360** | **3.6E-08** |
|  | **rs6591** | **0.008** | **0.001** | **0.009** | **40.558** | **1.9E-10** |
|  | **rs7952436** | **-0.034** | **0.002** | **-0.022** | **220.602** | **6.7004E-50** |
|  | **rs744205** | **0.011** | **0.001** | **0.013** | **73.521** | **1E-17** |
|  | **rs1813212** | **-0.009** | **0.001** | **-0.010** | **47.099** | **6.7004E-12** |
|  | **rs76693355** | **-0.014** | **0.002** | **-0.011** | **55.573** | **8.9991E-14** |
|  | **rs2900208** | **0.014** | **0.001** | **0.016** | **115.942** | **4.9E-27** |
|  | **rs10843397** | **0.009** | **0.001** | **0.010** | **42.355** | **7.5998E-11** |
|  | **rs11052457** | **0.023** | **0.003** | **0.010** | **49.358** | **2.0999E-12** |
|  | **rs2897968** | **0.016** | **0.001** | **0.018** | **150.402** | **1.3999E-34** |
|  | **rs3730071** | **-0.023** | **0.004** | **-0.009** | **39.567** | **3.2E-10** |
|  | **rs3782232** | **-0.020** | **0.002** | **-0.012** | **70.594** | **4.4005E-17** |
|  | **rs1351394** | **-0.026** | **0.001** | **-0.031** | **432.221** | **5.3003E-96** |
|  | **rs1168768** | **0.022** | **0.004** | **0.008** | **31.008** | **2.6E-08** |
|  | **rs9634212** | **0.024** | **0.002** | **0.023** | **249.157** | **4.0004E-56** |
|  | **rs7312646** | **-0.011** | **0.001** | **-0.012** | **69.133** | **9.2003E-17** |
|  | **rs34825238** | **0.009** | **0.001** | **0.009** | **37.979** | **7.1E-10** |
|  | **rs2287163** | **-0.010** | **0.001** | **-0.012** | **60.234** | **8.4004E-15** |
|  | **rs10773172** | **0.017** | **0.001** | **0.018** | **151.907** | **6.5993E-35** |
|  | **rs12820906** | **0.012** | **0.001** | **0.012** | **66.293** | **3.9003E-16** |
|  | **rs11613704** | **0.016** | **0.002** | **0.015** | **103.925** | **2.0999E-24** |
|  | **rs67551338** | **0.026** | **0.003** | **0.014** | **95.561** | **1.3999E-22** |
|  | **rs76895963** | **0.095** | **0.005** | **0.029** | **392.183** | **2.8003E-87** |
|  | **rs10770705** | **-0.008** | **0.001** | **-0.009** | **39.780** | **2.8E-10** |
|  | **rs7134283** | **-0.012** | **0.001** | **-0.013** | **73.195** | **1.2001E-17** |
|  | **rs774214** | **-0.009** | **0.001** | **-0.010** | **46.982** | **7.1995E-12** |
|  | **rs310796** | **0.011** | **0.001** | **0.012** | **67.496** | **2.0999E-16** |
|  | **rs2229840** | **0.020** | **0.002** | **0.018** | **145.715** | **1.5E-33** |
|  | **rs10848288** | **-0.008** | **0.001** | **-0.008** | **31.626** | **1.9E-08** |
|  | **rs117451679** | **0.013** | **0.002** | **0.009** | **38.661** | **5E-10** |
|  | **rs7132908** | **0.016** | **0.001** | **0.018** | **153.586** | **2.9E-35** |
|  | **rs703593** | **-0.011** | **0.001** | **-0.014** | **84.382** | **4.1002E-20** |
|  | **rs11065015** | **-0.028** | **0.004** | **-0.011** | **52.593** | **4.1002E-13** |
|  | **rs4980826** | **0.008** | **0.001** | **0.009** | **37.464** | **9.2999E-10** |
|  | **rs73129767** | **-0.010** | **0.002** | **-0.010** | **44.002** | **3.2999E-11** |
|  | **rs7301341** | **-0.010** | **0.001** | **-0.011** | **60.045** | **9.3004E-15** |
|  | **rs11833839** | **0.017** | **0.003** | **0.009** | **38.086** | **6.8E-10** |
|  | **rs35756741** | **-0.016** | **0.002** | **-0.011** | **54.439** | **1.5999E-13** |
|  | **rs11049684** | **-0.011** | **0.001** | **-0.012** | **61.350** | **4.7995E-15** |
|  | **rs1049193** | **-0.013** | **0.002** | **-0.012** | **65.957** | **4.6005E-16** |
|  | **rs10748128** | **0.012** | **0.001** | **0.014** | **89.446** | **3.1996E-21** |
|  | **rs12427047** | **-0.010** | **0.001** | **-0.010** | **44.909** | **2.0999E-11** |
|  | **rs10777860** | **-0.011** | **0.001** | **-0.013** | **73.996** | **7.8001E-18** |
|  | **rs11065979** | **-0.013** | **0.001** | **-0.016** | **116.213** | **4.3003E-27** |
|  | **rs1971955** | **-0.014** | **0.002** | **-0.012** | **62.304** | **2.9E-15** |
|  | **rs11611651** | **0.017** | **0.002** | **0.011** | **57.039** | **4.3003E-14** |
|  | **rs7994814** | **0.011** | **0.001** | **0.013** | **74.609** | **5.7003E-18** |
|  | **rs1218826** | **0.009** | **0.001** | **0.010** | **47.692** | **5.0004E-12** |
|  | **rs9317002** | **0.011** | **0.001** | **0.013** | **71.835** | **2.2999E-17** |
|  | **rs10870597** | **-0.010** | **0.001** | **-0.011** | **50.808** | **1E-12** |
|  | **rs2225226** | **-0.030** | **0.002** | **-0.029** | **387.369** | **3.1003E-86** |
|  | **rs7321045** | **0.009** | **0.001** | **0.010** | **45.828** | **1.2999E-11** |
|  | **rs146851424** | **0.060** | **0.004** | **0.021** | **191.342** | **1.5999E-43** |
|  | **rs9596810** | **-0.009** | **0.001** | **-0.010** | **47.806** | **4.7E-12** |
|  | **rs9540493** | **-0.009** | **0.001** | **-0.010** | **50.095** | **1.5E-12** |
|  | **rs1924936** | **0.015** | **0.001** | **0.015** | **105.881** | **7.8001E-25** |
|  | **rs8002779** | **-0.008** | **0.001** | **-0.009** | **36.490** | **1.5E-09** |
|  | **rs7994783** | **-0.008** | **0.001** | **-0.009** | **38.963** | **4.3E-10** |
|  | **rs1176314** | **-0.007** | **0.001** | **-0.009** | **34.851** | **3.6E-09** |
|  | **rs9513510** | **-0.011** | **0.001** | **-0.012** | **64.415** | **1E-15** |
|  | **rs3783256** | **-0.009** | **0.001** | **-0.011** | **51.642** | **6.7004E-13** |
|  | **rs6563808** | **-0.009** | **0.001** | **-0.009** | **36.333** | **1.7E-09** |
|  | **rs67141907** | **0.011** | **0.002** | **0.009** | **40.820** | **1.7E-10** |
|  | **rs3212260** | **0.013** | **0.001** | **0.014** | **84.638** | **3.6E-20** |
|  | **rs2296316** | **-0.010** | **0.001** | **-0.012** | **68.557** | **1.2001E-16** |
|  | **rs6575340** | **0.008** | **0.001** | **0.009** | **39.296** | **3.6E-10** |
|  | **rs17197114** | **0.011** | **0.002** | **0.010** | **43.928** | **3.4002E-11** |
|  | **rs12889702** | **0.009** | **0.001** | **0.010** | **47.207** | **6.4003E-12** |
|  | **rs2884345** | **-0.008** | **0.001** | **-0.008** | **31.371** | **2.1E-08** |
|  | **rs7141420** | **0.013** | **0.001** | **0.016** | **109.548** | **1.2001E-25** |
|  | **rs12879423** | **0.014** | **0.001** | **0.016** | **112.091** | **3.4002E-26** |
|  | **rs8007644** | **0.008** | **0.001** | **0.009** | **39.410** | **3.4E-10** |
|  | **rs112957890** | **0.010** | **0.001** | **0.010** | **44.835** | **2.0999E-11** |
|  | **rs112560164** | **0.013** | **0.002** | **0.012** | **68.480** | **1.2999E-16** |
|  | **rs11160601** | **0.016** | **0.002** | **0.011** | **51.677** | **6.4998E-13** |
|  | **rs61992671** | **-0.011** | **0.001** | **-0.012** | **68.254** | **1.3999E-16** |
|  | **rs9783665** | **-0.007** | **0.001** | **-0.008** | **32.306** | **1.3E-08** |
|  | **rs36100359** | **-0.012** | **0.002** | **-0.010** | **41.187** | **1.4E-10** |
|  | **rs9788443** | **0.019** | **0.003** | **0.010** | **42.871** | **5.7996E-11** |
|  | **rs4899012** | **-0.021** | **0.001** | **-0.025** | **283.801** | **1.1E-63** |
|  | **rs7150606** | **-0.010** | **0.002** | **-0.008** | **30.456** | **3.4E-08** |
|  | **rs2332175** | **-0.008** | **0.001** | **-0.009** | **37.414** | **9.6E-10** |
|  | **rs4906203** | **-0.009** | **0.001** | **-0.009** | **39.972** | **2.6E-10** |
|  | **rs56130943** | **0.009** | **0.002** | **0.009** | **35.279** | **2.9E-09** |
|  | **rs11855017** | **0.013** | **0.002** | **0.012** | **65.868** | **4.7995E-16** |
|  | **rs113978196** | **-0.008** | **0.001** | **-0.008** | **31.747** | **1.8E-08** |
|  | **rs62621400** | **-0.020** | **0.003** | **-0.011** | **55.411** | **9.7994E-14** |
|  | **rs2663125** | **-0.009** | **0.001** | **-0.010** | **44.022** | **3.1996E-11** |
|  | **rs11071182** | **0.013** | **0.002** | **0.011** | **51.745** | **6.2994E-13** |
|  | **rs338361** | **0.009** | **0.001** | **0.010** | **48.151** | **3.9003E-12** |
|  | **rs12906197** | **-0.011** | **0.001** | **-0.013** | **73.628** | **9.3994E-18** |
|  | **rs76875574** | **-0.016** | **0.003** | **-0.008** | **30.940** | **2.7E-08** |
|  | **rs6493534** | **0.007** | **0.001** | **0.008** | **30.354** | **3.6E-08** |
|  | **rs933807** | **-0.012** | **0.001** | **-0.014** | **87.684** | **7.7002E-21** |
|  | **rs3809569** | **0.014** | **0.001** | **0.014** | **94.788** | **2.0999E-22** |
|  | **rs1521624** | **-0.009** | **0.001** | **-0.011** | **52.655** | **4.0004E-13** |
|  | **rs16942324** | **-0.037** | **0.004** | **-0.014** | **94.672** | **2.1999E-22** |
|  | **rs1573891** | **-0.022** | **0.002** | **-0.019** | **165.405** | **7.5007E-38** |
|  | **rs35874463** | **0.018** | **0.003** | **0.010** | **46.394** | **9.7006E-12** |
|  | **rs5742915** | **0.009** | **0.001** | **0.011** | **57.627** | **3.1996E-14** |
|  | **rs12907384** | **-0.012** | **0.001** | **-0.014** | **93.200** | **4.7E-22** |
|  | **rs11648796** | **0.015** | **0.002** | **0.015** | **100.115** | **1.3999E-23** |
|  | **rs71385734** | **-0.023** | **0.002** | **-0.021** | **191.945** | **1.2001E-43** |
|  | **rs876672** | **-0.013** | **0.002** | **-0.009** | **35.050** | **3.2E-09** |
|  | **rs7188009** | **0.009** | **0.001** | **0.010** | **47.029** | **7E-12** |
|  | **rs2531991** | **0.016** | **0.001** | **0.017** | **131.113** | **2.2999E-30** |
|  | **rs55727637** | **-0.012** | **0.001** | **-0.014** | **83.596** | **6.0996E-20** |
|  | **rs2726036** | **0.012** | **0.001** | **0.014** | **87.614** | **8.0002E-21** |
|  | **rs72801843** | **0.016** | **0.001** | **0.018** | **143.827** | **3.9003E-33** |
|  | **rs71393968** | **0.021** | **0.003** | **0.011** | **51.583** | **6.8992E-13** |
|  | **rs8059189** | **-0.012** | **0.001** | **-0.014** | **84.236** | **4.4005E-20** |
|  | **rs7192870** | **-0.009** | **0.001** | **-0.010** | **49.438** | **1.9999E-12** |
|  | **rs56094641** | **0.036** | **0.001** | **0.042** | **812.490** | **1E-178** |
|  | **rs34017457** | **0.049** | **0.007** | **0.010** | **42.551** | **6.8992E-11** |
|  | **rs76513770** | **-0.016** | **0.002** | **-0.013** | **74.830** | **5.1004E-18** |
|  | **rs4887925** | **-0.008** | **0.001** | **-0.009** | **36.892** | **1.2E-09** |
|  | **rs9925273** | **-0.012** | **0.002** | **-0.011** | **53.392** | **2.7002E-13** |
|  | **rs9938120** | **-0.018** | **0.002** | **-0.015** | **105.528** | **9.3994E-25** |
|  | **rs4788218** | **0.022** | **0.001** | **0.025** | **295.209** | **3.6E-66** |
|  | **rs6500249** | **-0.008** | **0.001** | **-0.009** | **33.714** | **6.4E-09** |
|  | **rs10775348** | **0.013** | **0.001** | **0.014** | **90.385** | **1.9999E-21** |
|  | **rs25849** | **0.017** | **0.001** | **0.018** | **150.511** | **1.2999E-34** |
|  | **rs3751866** | **-0.008** | **0.001** | **-0.008** | **31.637** | **1.9E-08** |
|  | **rs10775406** | **-0.010** | **0.001** | **-0.010** | **43.286** | **4.7E-11** |
|  | **rs7503332** | **0.008** | **0.001** | **0.009** | **37.450** | **9.4001E-10** |
|  | **rs2252909** | **-0.007** | **0.001** | **-0.008** | **31.396** | **2.1E-08** |
|  | **rs2521349** | **0.008** | **0.001** | **0.009** | **36.425** | **1.6E-09** |
|  | **rs114177791** | **0.010** | **0.002** | **0.010** | **44.406** | **2.7002E-11** |
|  | **rs36000545** | **-0.017** | **0.001** | **-0.019** | **165.226** | **8.1997E-38** |
|  | **rs55831773** | **-0.015** | **0.002** | **-0.014** | **84.369** | **4.1002E-20** |
|  | **rs78378222** | **0.082** | **0.006** | **0.021** | **202.053** | **7.3995E-46** |
|  | **rs4795318** | **0.009** | **0.001** | **0.011** | **51.507** | **7.1007E-13** |
|  | **rs6416914** | **-0.009** | **0.001** | **-0.010** | **42.668** | **6.4998E-11** |
|  | **rs67560975** | **0.013** | **0.002** | **0.010** | **43.970** | **3.2999E-11** |
|  | **rs77093479** | **-0.010** | **0.002** | **-0.009** | **38.052** | **6.9E-10** |
|  | **rs9898189** | **-0.008** | **0.001** | **-0.009** | **35.919** | **2.1E-09** |
|  | **rs3110496** | **0.009** | **0.001** | **0.010** | **43.190** | **5.0004E-11** |
|  | **rs7223535** | **-0.025** | **0.001** | **-0.027** | **320.718** | **1E-71** |
|  | **rs2015561** | **-0.012** | **0.001** | **-0.014** | **85.929** | **1.9002E-20** |
|  | **rs8067974** | **-0.014** | **0.001** | **-0.015** | **108.650** | **1.9002E-25** |
|  | **rs34055910** | **-0.008** | **0.001** | **-0.009** | **34.157** | **5.1E-09** |
|  | **rs4968799** | **-0.018** | **0.002** | **-0.014** | **87.780** | **7.2996E-21** |
|  | **rs4525525** | **-0.009** | **0.001** | **-0.010** | **42.862** | **5.9007E-11** |
|  | **rs8074074** | **0.009** | **0.001** | **0.010** | **42.587** | **6.7999E-11** |
|  | **rs9892365** | **-0.016** | **0.001** | **-0.018** | **142.184** | **8.9002E-33** |
|  | **rs2005172** | **0.023** | **0.001** | **0.026** | **297.694** | **1E-66** |
|  | **rs236587** | **-0.008** | **0.001** | **-0.009** | **33.772** | **6.2E-09** |
|  | **rs498685** | **-0.007** | **0.001** | **-0.008** | **31.314** | **2.2E-08** |
|  | **rs8091374** | **-0.010** | **0.002** | **-0.008** | **32.733** | **1.1E-08** |
|  | **rs4800670** | **-0.007** | **0.001** | **-0.008** | **31.257** | **2.3E-08** |
|  | **rs1941697** | **0.008** | **0.001** | **0.009** | **37.305** | **1E-09** |
|  | **rs33973388** | **0.012** | **0.001** | **0.015** | **96.336** | **9.7006E-23** |
|  | **rs9960619** | **0.010** | **0.001** | **0.011** | **55.652** | **8.6996E-14** |
|  | **rs9951619** | **0.012** | **0.001** | **0.012** | **70.778** | **4.0004E-17** |
|  | **rs12967135** | **0.043** | **0.001** | **0.044** | **866.083** | **2.301E-190** |
|  | **rs8088739** | **0.008** | **0.001** | **0.008** | **32.290** | **1.3E-08** |
|  | **rs74494415** | **-0.025** | **0.003** | **-0.012** | **61.431** | **4.6005E-15** |
|  | **rs11663903** | **0.008** | **0.001** | **0.009** | **35.994** | **2E-09** |
|  | **rs7229520** | **-0.010** | **0.001** | **-0.012** | **63.017** | **1.9999E-15** |
|  | **rs57126421** | **-0.011** | **0.001** | **-0.011** | **52.769** | **3.8001E-13** |
|  | **rs4369779** | **0.026** | **0.002** | **0.025** | **291.330** | **2.6002E-65** |
|  | **rs55854145** | **-0.015** | **0.003** | **-0.008** | **31.135** | **2.4E-08** |
|  | **rs17066856** | **-0.021** | **0.002** | **-0.014** | **92.875** | **5.6002E-22** |
|  | **rs2602713** | **0.011** | **0.001** | **0.013** | **77.641** | **1.2001E-18** |
|  | **rs284662** | **-0.009** | **0.001** | **-0.011** | **53.829** | **2.1999E-13** |
|  | **rs12981554** | **-0.009** | **0.001** | **-0.010** | **46.556** | **8.9002E-12** |
|  | **rs7245985** | **-0.011** | **0.002** | **-0.010** | **50.103** | **1.5E-12** |
|  | **rs29946** | **0.007** | **0.001** | **0.009** | **34.394** | **4.5E-09** |
|  | **rs281385** | **-0.012** | **0.002** | **-0.009** | **40.786** | **1.7E-10** |
|  | **rs147110934** | **-0.032** | **0.004** | **-0.012** | **61.034** | **5.6002E-15** |
|  | **rs10432304** | **0.007** | **0.001** | **0.009** | **34.259** | **4.8E-09** |
|  | **rs58857770** | **-0.010** | **0.001** | **-0.011** | **55.901** | **7.5998E-14** |
|  | **rs34831515** | **-0.008** | **0.001** | **-0.008** | **32.337** | **1.3E-08** |
|  | **rs111640872** | **0.014** | **0.001** | **0.016** | **118.903** | **1.1E-27** |
|  | **rs3810291** | **0.017** | **0.001** | **0.019** | **163.081** | **2.3999E-37** |
|  | **rs11880992** | **0.014** | **0.001** | **0.016** | **120.611** | **4.6005E-28** |
|  | **rs62621197** | **-0.033** | **0.003** | **-0.014** | **91.011** | **1.3999E-21** |
|  | **rs73004967** | **-0.017** | **0.002** | **-0.010** | **45.792** | **1.2999E-11** |
|  | **rs35050648** | **0.008** | **0.001** | **0.008** | **31.979** | **1.6E-08** |
|  | **rs2163832** | **-0.013** | **0.001** | **-0.015** | **101.725** | **6.4003E-24** |
|  | **rs1407031** | **-0.008** | **0.001** | **-0.010** | **41.419** | **1.2E-10** |
|  | **rs6514066** | **0.007** | **0.001** | **0.008** | **31.075** | **2.5E-08** |
|  | **rs6026578** | **-0.010** | **0.001** | **-0.011** | **56.114** | **6.7999E-14** |
|  | **rs2427320** | **-0.008** | **0.001** | **-0.008** | **31.096** | **2.5E-08** |
|  | **rs116165844** | **-0.011** | **0.002** | **-0.009** | **34.475** | **4.3E-09** |
|  | **rs6085658** | **-0.009** | **0.001** | **-0.010** | **45.354** | **1.5999E-11** |
|  | **rs143384** | **0.035** | **0.001** | **0.041** | **779.566** | **1.5E-171** |
|  | **rs34879158** | **-0.022** | **0.001** | **-0.023** | **231.976** | **2.1999E-52** |
|  | **rs237738** | **0.009** | **0.002** | **0.009** | **33.456** | **7.3E-09** |
|  | **rs6081869** | **-0.010** | **0.001** | **-0.012** | **61.502** | **4.4005E-15** |
|  | **rs2252720** | **-0.014** | **0.001** | **-0.015** | **102.389** | **4.6005E-24** |
|  | **rs6142059** | **0.010** | **0.001** | **0.011** | **59.756** | **1.1E-14** |
|  | **rs13043303** | **-0.019** | **0.002** | **-0.017** | **133.735** | **6.2001E-31** |
|  | **rs73619441** | **-0.013** | **0.002** | **-0.010** | **50.075** | **1.5E-12** |
|  | **rs1780672** | **0.013** | **0.002** | **0.012** | **62.852** | **2.1999E-15** |
|  | **rs4819021** | **-0.008** | **0.001** | **-0.009** | **38.200** | **6.4E-10** |
|  | **rs9976812** | **-0.014** | **0.001** | **-0.016** | **115.499** | **6.0996E-27** |
|  | **rs7280982** | **-0.009** | **0.002** | **-0.009** | **34.521** | **4.2E-09** |
|  | **rs1043801** | **0.015** | **0.003** | **0.008** | **31.702** | **1.8E-08** |
|  | **rs73189390** | **-0.009** | **0.002** | **-0.008** | **31.258** | **2.3E-08** |
|  | **rs35665085** | **-0.017** | **0.003** | **-0.009** | **37.479** | **9.2001E-10** |
|  | **rs4821940** | **-0.007** | **0.001** | **-0.008** | **31.081** | **2.5E-08** |
|  | **rs41311445** | **-0.024** | **0.002** | **-0.017** | **131.916** | **1.5999E-30** |
|  | **rs5771118** | **0.009** | **0.001** | **0.009** | **36.218** | **1.8E-09** |
|  | **rs165656** | **0.008** | **0.001** | **0.010** | **42.233** | **8.1003E-11** |
|  | **rs5753630** | **0.007** | **0.001** | **0.009** | **33.634** | **6.6999E-09** |
|  | **rs118173451** | **-0.029** | **0.005** | **-0.009** | **33.739** | **6.3E-09** |
|  | **rs113619763** | **0.017** | **0.003** | **0.010** | **42.257** | **8.0002E-11** |
|  | **rs5752989** | **-0.010** | **0.001** | **-0.012** | **62.318** | **2.9E-15** |
| Usual walking pace |  |  |  |  |  |  |
|  | **rs12739999** | **-0.014** | **0.002** | **-0.012** | **46.919** | **7.4114E-12** |
|  | **rs75854315** | **-0.017** | **0.003** | **-0.010** | **32.440** | **1.2305E-08** |
|  | **rs7560257** | **-0.011** | **0.002** | **-0.010** | **30.957** | **2.6408E-08** |
|  | **rs61134960** | **0.010** | **0.002** | **0.010** | **31.687** | **1.8123E-08** |
|  | **rs5026760** | **0.011** | **0.002** | **0.010** | **30.904** | **2.7127E-08** |
|  | **rs2054079** | **0.010** | **0.002** | **0.011** | **37.932** | **7.3347E-10** |
|  | **rs10865958** | **-0.010** | **0.001** | **-0.012** | **44.691** | **2.3105E-11** |
|  | **rs9844666** | **-0.010** | **0.002** | **-0.010** | **30.805** | **2.8558E-08** |
|  | **rs13107325** | **-0.026** | **0.003** | **-0.016** | **84.471** | **3.9201E-20** |
|  | **rs9637592** | **-0.009** | **0.002** | **-0.010** | **32.318** | **1.3097E-08** |
|  | **rs9379843** | **0.009** | **0.001** | **0.010** | **34.331** | **4.6533E-09** |
|  | **rs4839898** | **0.014** | **0.002** | **0.010** | **34.001** | **5.5146E-09** |
|  | **rs9471333** | **0.009** | **0.001** | **0.010** | **33.326** | **7.799E-09** |
|  | **rs11152989** | **-0.010** | **0.002** | **-0.010** | **35.590** | **2.4372E-09** |
|  | **rs4509216** | **-0.008** | **0.001** | **-0.010** | **32.385** | **1.2655E-08** |
|  | **rs9791848** | **-0.009** | **0.002** | **-0.009** | **30.023** | **4.2734E-08** |
|  | **rs13238384** | **0.010** | **0.002** | **0.011** | **43.927** | **3.4135E-11** |
|  | **rs7896518** | **0.009** | **0.002** | **0.010** | **35.325** | **2.7927E-09** |
|  | **rs10828258** | **-0.010** | **0.002** | **-0.011** | **38.044** | **6.9236E-10** |
|  | **rs7124681** | **-0.010** | **0.002** | **-0.012** | **44.833** | **2.1498E-11** |
|  | **rs10750025** | **-0.009** | **0.002** | **-0.010** | **31.217** | **2.3095E-08** |
|  | **rs10862220** | **0.010** | **0.002** | **0.011** | **42.030** | **9.0012E-11** |
|  | **rs10149134** | **-0.009** | **0.002** | **-0.011** | **38.348** | **5.9261E-10** |
|  | **rs7140836** | **-0.009** | **0.002** | **-0.010** | **33.676** | **6.5145E-09** |
|  | **rs4780421** | **0.009** | **0.002** | **0.010** | **33.765** | **6.2247E-09** |
|  | **rs34898535** | **0.008** | **0.002** | **0.009** | **30.220** | **3.86E-08** |
|  | **rs9972653** | **-0.010** | **0.002** | **-0.012** | **45.915** | **1.2371E-11** |
|  | **rs1652376** | **0.009** | **0.001** | **0.010** | **36.087** | **1.8886E-09** |
|  | **rs784257** | **-0.014** | **0.002** | **-0.013** | **53.730** | **2.3057E-13** |
|  | **rs273505** | **-0.010** | **0.002** | **-0.011** | **44.169** | **3.0165E-11** |
