# Supplementary figures and images for: Causal associations between the insulin-like growth factor family and sarcopenia: a bidirectional Mendelian randomization study
Source: Front Endocrinol (Lausanne). 2024 Oct 23;15:1422472. doi: 10.3389/fendo.2024.1422472 (PMC11537870; doi:10.3389/fendo.2024.1422472)

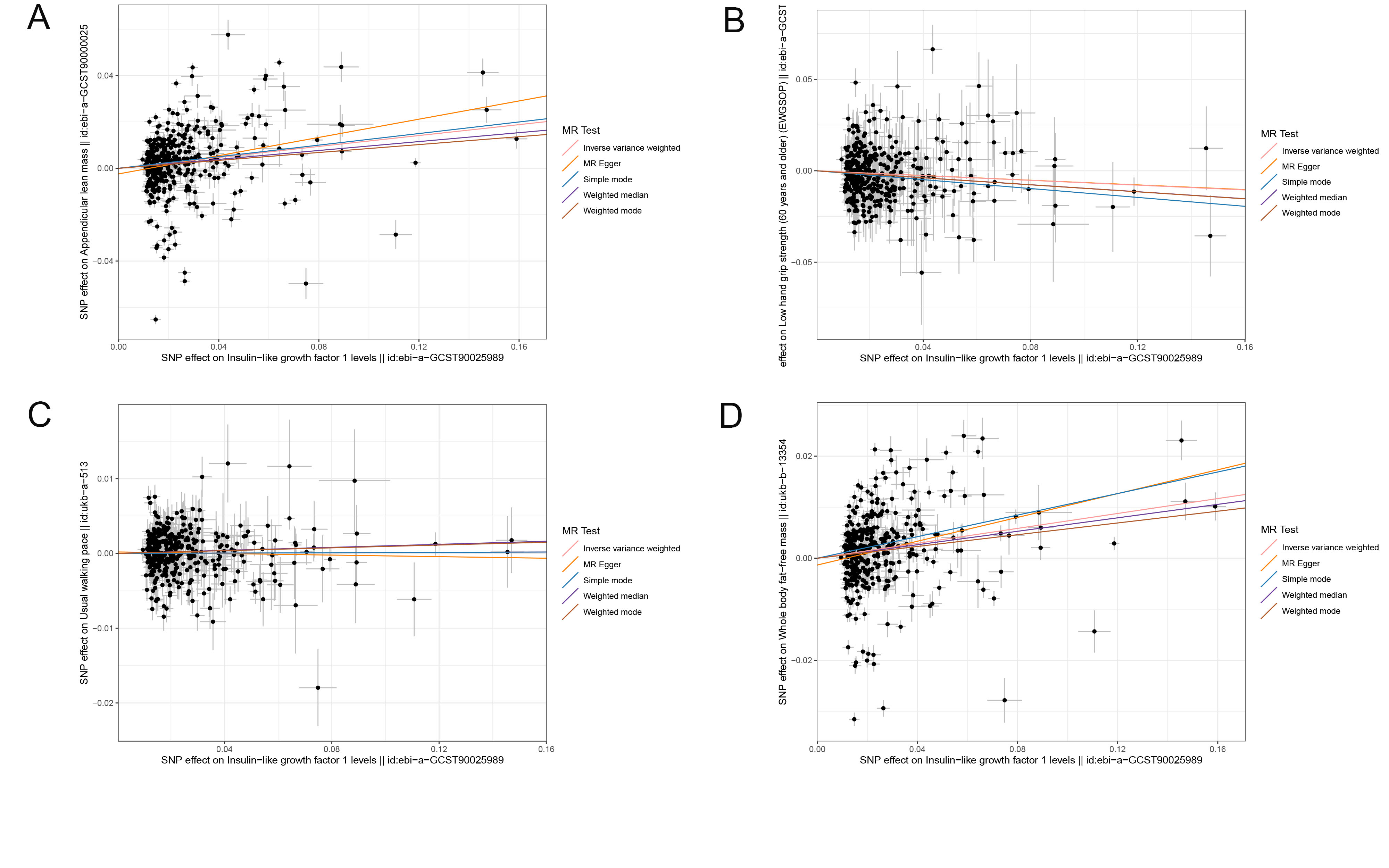

Supplement: Supplementary Figure 1 — The SNPs effect estimate based on the forward MR analysis. (A) IGF-1 on appendicular lean mass; (B) IGF-1 on low hand grip strength; (C) IGF-1 on usual walking pace; (D) IGF-1 on whole body fat-free mass. SNPs, single nucleotide polymorphisms. [file Image1.jpeg]

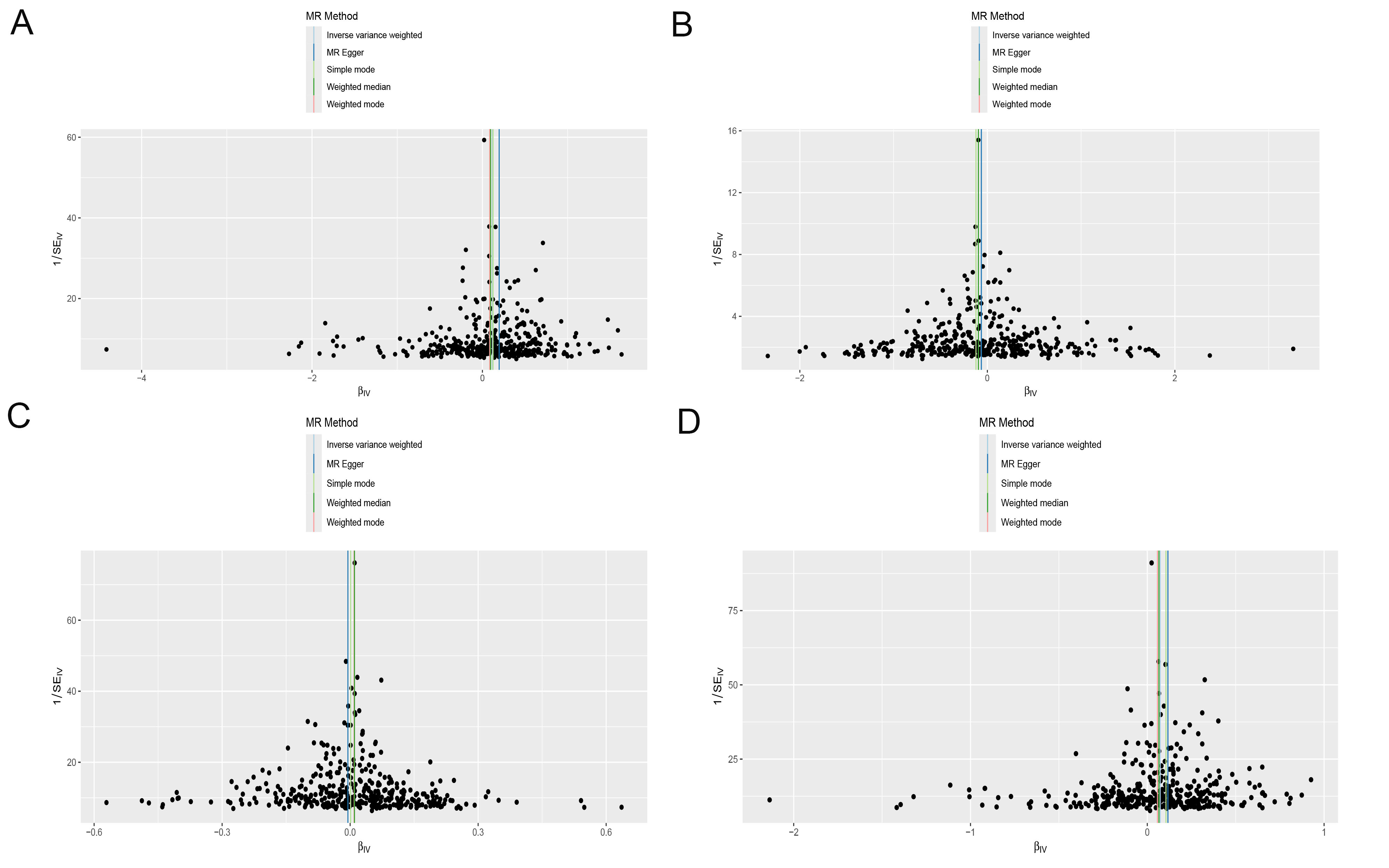

Supplement: Supplementary Figure 2 — The funnel plot performed by the forward MR analysis. (A) IGF-1 on appendicular lean mass; (B) IGF-1 on low hand grip strength; (C) IGF-1 on usual walking pace; (D) IGF-1 on whole body fat-free mass. [file Image2.jpeg]

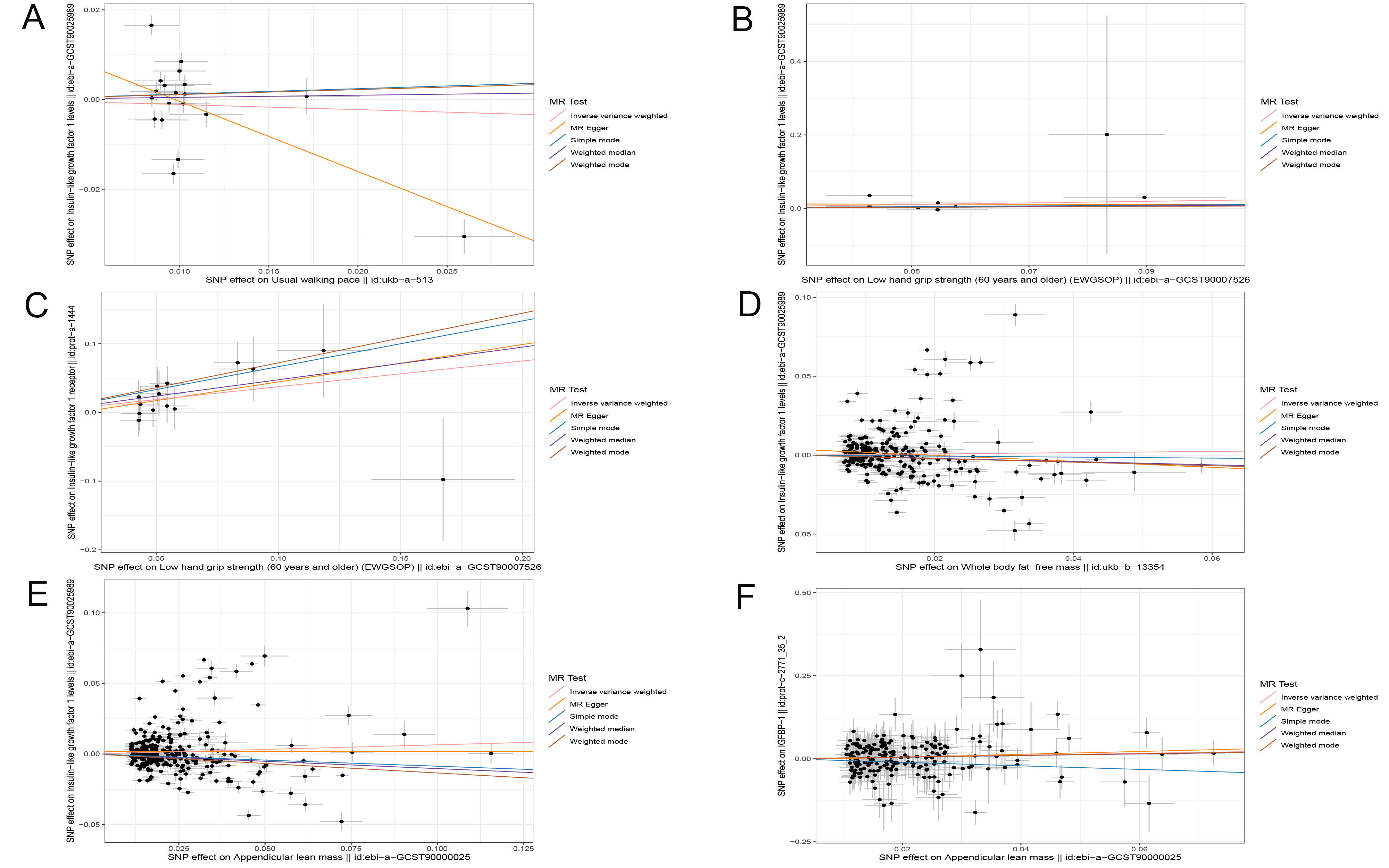

Supplement: Supplementary Figure 3 — The SNPs effect estimate based on the reverse MR analysis. (A) usual walking pace on IGF-1; (B) low hand grip strength on IGF-1; (C) low hand grip strength on IGF-1R; (D) whole body fat-free mass on IGF-1; (E) appendicular lean mass on IGF-1; (F) appendicular lean mass on IGFBP-1. SNPs, single nucleotide polymorphisms. [file Image3.jpeg]

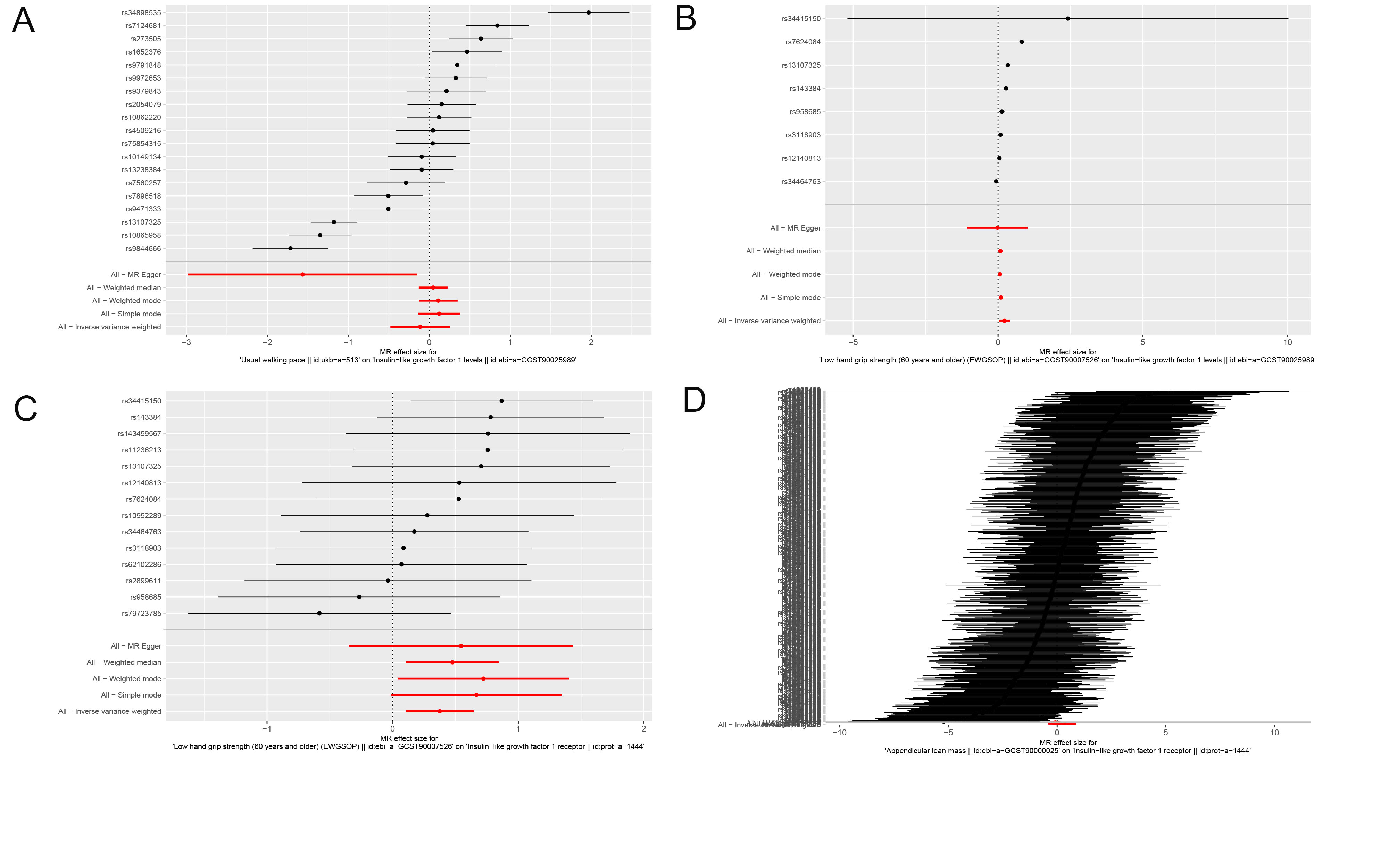

Supplement: Supplementary Figure 4 — The forest plot performed by the reverse MR analysis. (A) usual walking pace on IGF-1; (B) low hand grip strength on IGF-1; (C) low hand grip strength on IGF-1R; (D) appendicular lean mass on IGF-1. [file Image4.jpeg]

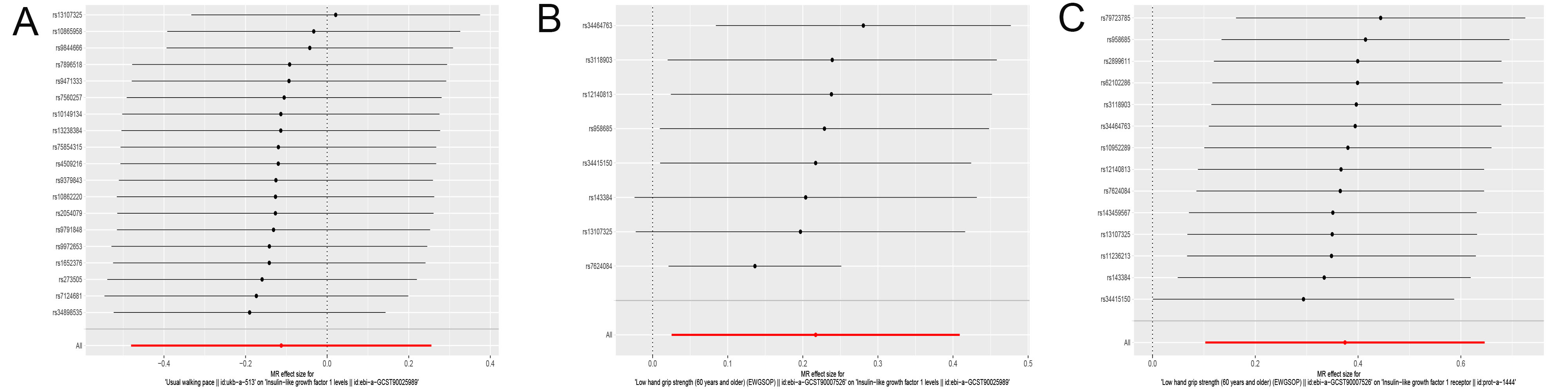

Supplement: Supplementary Figure 5 — The sensitivity analyses performed by the reverse MR analysis. (A) usual walking pace on IGF-1; (B) low hand grip strength on IGF-1; (C) low hand grip strength on IGF-1R. [file Image5.jpeg]

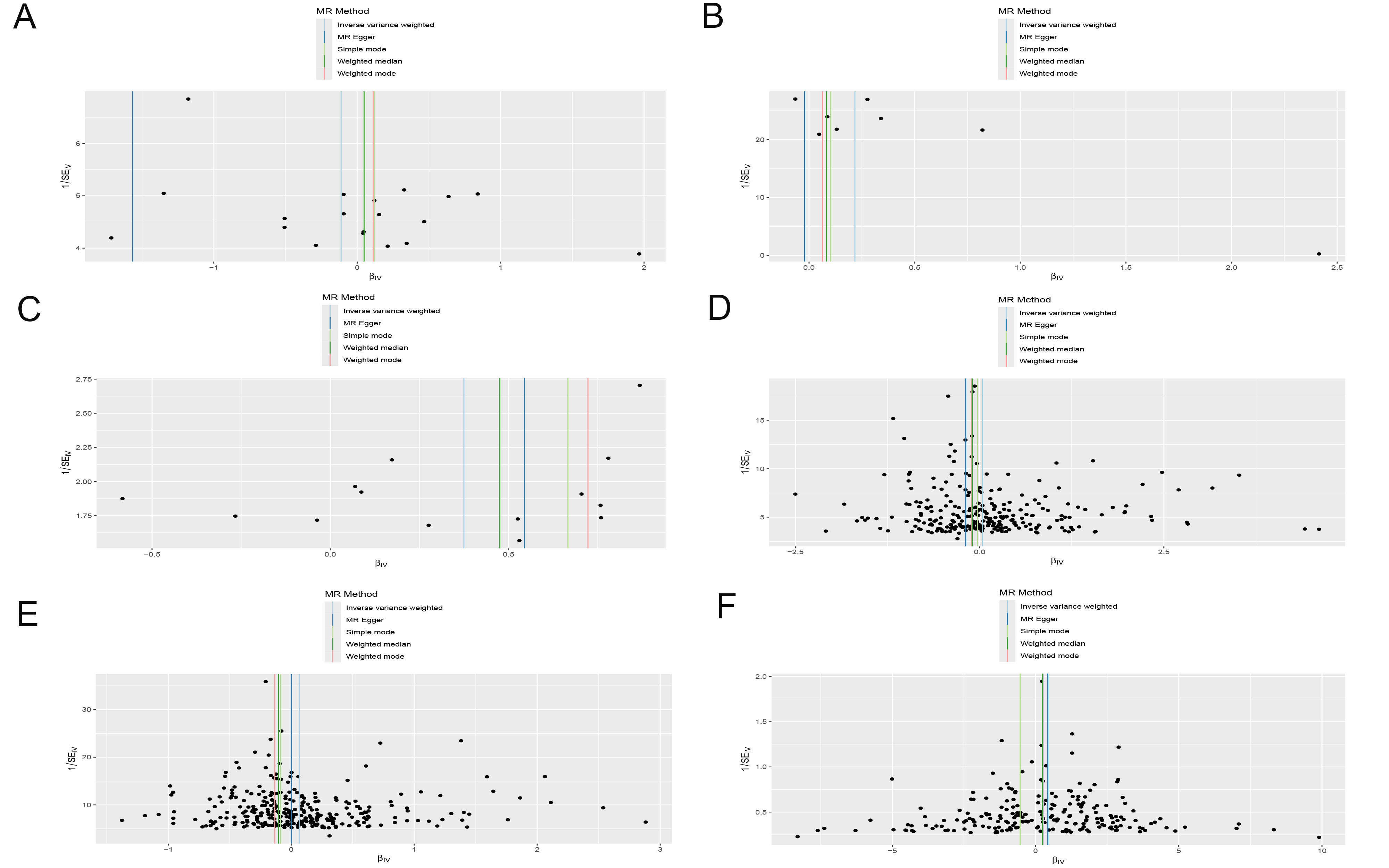

Supplement: Supplementary Figure 6 — The funnel plot performed by the reverse MR analysis. (A) usual walking pace on IGF-1; (B) low hand grip strength on IGF-1; (C) low hand grip strength on IGF-1R; (D) whole body fat-free mass on IGF-1; (E) appendicular lean mass on IGF-1; (F) appendicular lean mass on IGFBP-1. [file Image6.jpeg]
